# Supplementary material for: Myeloid cells coordinately induce glioma cell-intrinsic and cell-extrinsic pathways for chemoresistance via GP130 signaling
Source: Cell Rep Med. 2024 Jul 24;5(8):101658. doi: 10.1016/j.xcrm.2024.101658 (PMC11384956; doi:10.1016/j.xcrm.2024.101658)
Supplement: Document S1. Figures S1–S20 [file mmc1.pdf]

Cell Reports Medicine, Volume *5*

Supplemental information

Myeloid cells coordinately induce glioma cell-intrinsic and cell-extrinsic pathways for chemoresistance via GP130 signaling

Jiying Cheng, Min Li, Edyta Motta, Deivi Barci, Wangyang Song, Ding Zhou, Gen Li, Sihan Zhu, Anru Yang, Brian D. Vaillant, Axel Imhof, Ignasi Forné, Sabine Spiegl-Kreinecker, Nu Zhang, Hiroshi Katayama, Krishna P.L. Bhat, Charlotte Flüh, Roland E. Kälin, and Rainer Glass

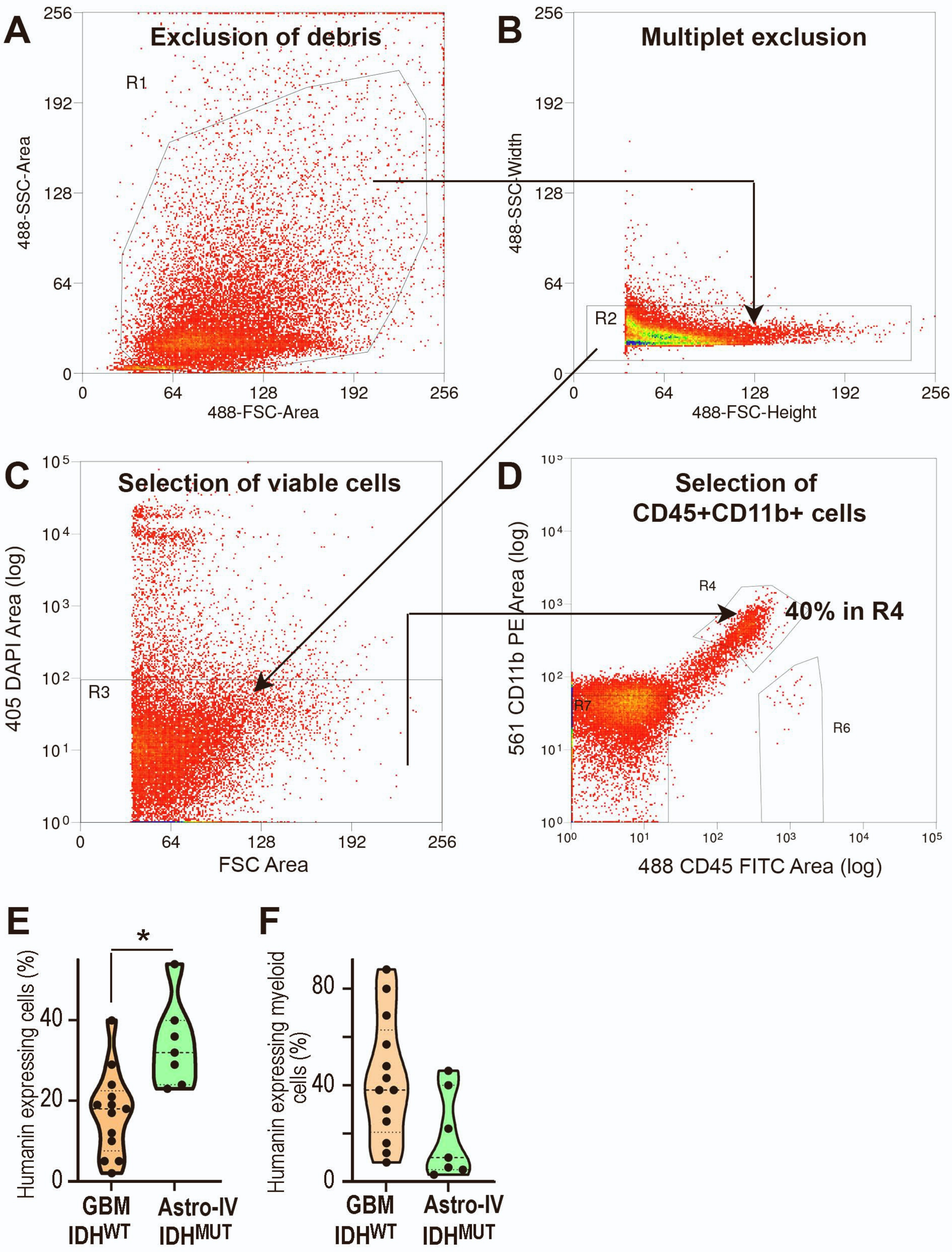

A
Exclusion of debris
R1
488-SSC-Area
488-FSC-Area
B
Multiplet exclusion
R2
488-SSC-Width
488-FSC-Height
C
Selection of viable cells
R3
405 DAPI Area (log)
FSC Area
D
Selection of CD45+CD11b+ cells
R4
40% in R4
R6
R7
561 CD11b PE Area (log)
488 CD45 FITC Area (log)
E
Humanin expressing cells (%)
GBM IDHWT
Astro-IV IDHMUT
F
Humanin expressing myeloid cells (%)
GBM IDHWT
Astro-IV IDHMUT

Figure S1: Flow cytometric gating strategy for purification of GBM associated myeloid cells (GAM). Related to Figure 1. Brain specimen (from GBM resection or epilepsy surgery) were dispersed into single cell suspensions, which were immunostained (for CD11b, CD45) and analyzed by flow cytometry. The strategy for the acquisition of FACS data is outlined: Debris was excluded **(A)** and analysis was restricted to single **(B)**, viable cells **(C)** with sound immunofluorescence signals (**D**; as compared to negative controls); the plot provides representative data of 13 independent FACS experiments and shows that viable myeloid cells (R4 gate) were readily purified from human specimen. **(E)** Quantification of Humanin-positive cells in IDH^{MUT} vs. IDH^{WT} tumors. **(F)** Preponderance of Humanin in GAMs of IDH^{WT} tumors is subject to inter-patient heterogeneity. Statistical significance is shown by ttest (*p < 0.05) in (**E**).

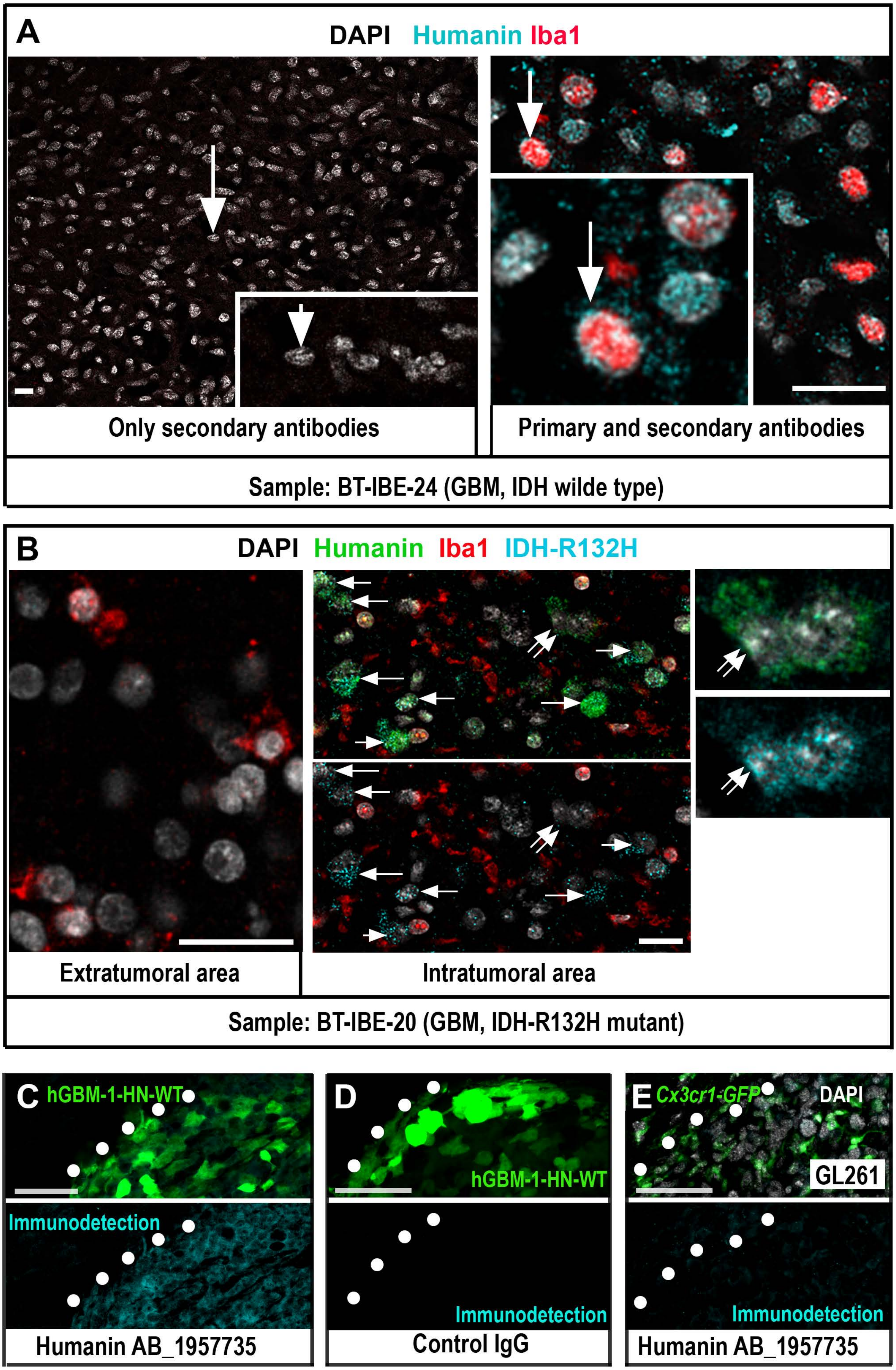
A
DAPI Humanin Iba1
Only secondary antibodies
Primary and secondary antibodies
Sample: BT-IBE-24 (GBM, IDH wilde type)
B
DAPI Humanin Iba1 IDH-R132H
Extratumoral area
Intratumoral area
Sample: BT-IBE-20 (GBM, IDH-R132H mutant)
C hGBM-1-HN-WT
Immunodetection
Humanin AB_1957735
D
hGBM-1-HN-WT
Immunodetection
Control IgG
E Cx3cr1-GFP
DAPI
GL261
Immunodetection
Humanin AB_1957735

Figure S2: Humanin is specifically detected in human GBM or astrocytoma cells and in GBM associated myeloid cells (GAM). Related to Figure 1. Samples from GBM patients **(A-B)** or mouse glioma models **(C-E)** were immunostained with or without Humanin antibody and the signal was analyzed by confocal microscopy. (**A)** A GBM specimen underwent the immunolabeling procedure (plus DAPI staining) with or without application of (primary) antibodies for Humanin and the myeloid marker Iba1. Incubation with seconday antibodies alone resulted in absence of immunostaining (while the DNA intercalating dye DAPI still labeled nuclei; arrow). Sequential application of primary and secondary antibodies resulted in immunolabeling for Iba1 or Humanin; partly cells were co-stained for both markers (arrow); the insets show enlargements of the cells marked by an arrow. (**B)** In IDH1-mutant (IDH1^{R132H}), grade-IV astrocytomas extra- and intra-tumoral areas were identified by IDH^{R132H} immunostaining. Colabeling for Humanin Iba1 and IDH^{R132H} was performed. To improve visibility of markers the central panel gives two views on the same samples: The upper part depicts labeling for all three markers; the lower part hides the Humanin channel. The arrows indicate IDH^{R132H}-positive GBM cells expressing Humanin (a magnification of the tumor cell marked by the double arrow is shown on the right). (**C, D**) Immunodetection for Humanin in an orthotopic mouse xenograft model with hGBM-1-HN-WT cells (expressing GFP). (C) Immunolabeling for Humanin (by the Humanin specific antibody AB_1957735) is confined to the tumor area in (dotted line) and was absent (D), in the same model, when using a non-immune IgG (control IgG, of the same isotype as the Humanin directed antibody). **(E)** Incubating samples from orthotopic murine gliomas (GL261) in *Cx3CR1-GFP* transgenic mice with AB_1957735 resulted in absence of immunolabeling. Scales bars indicate 30 μm (**A, B**), and 50 μm (**C-E**).

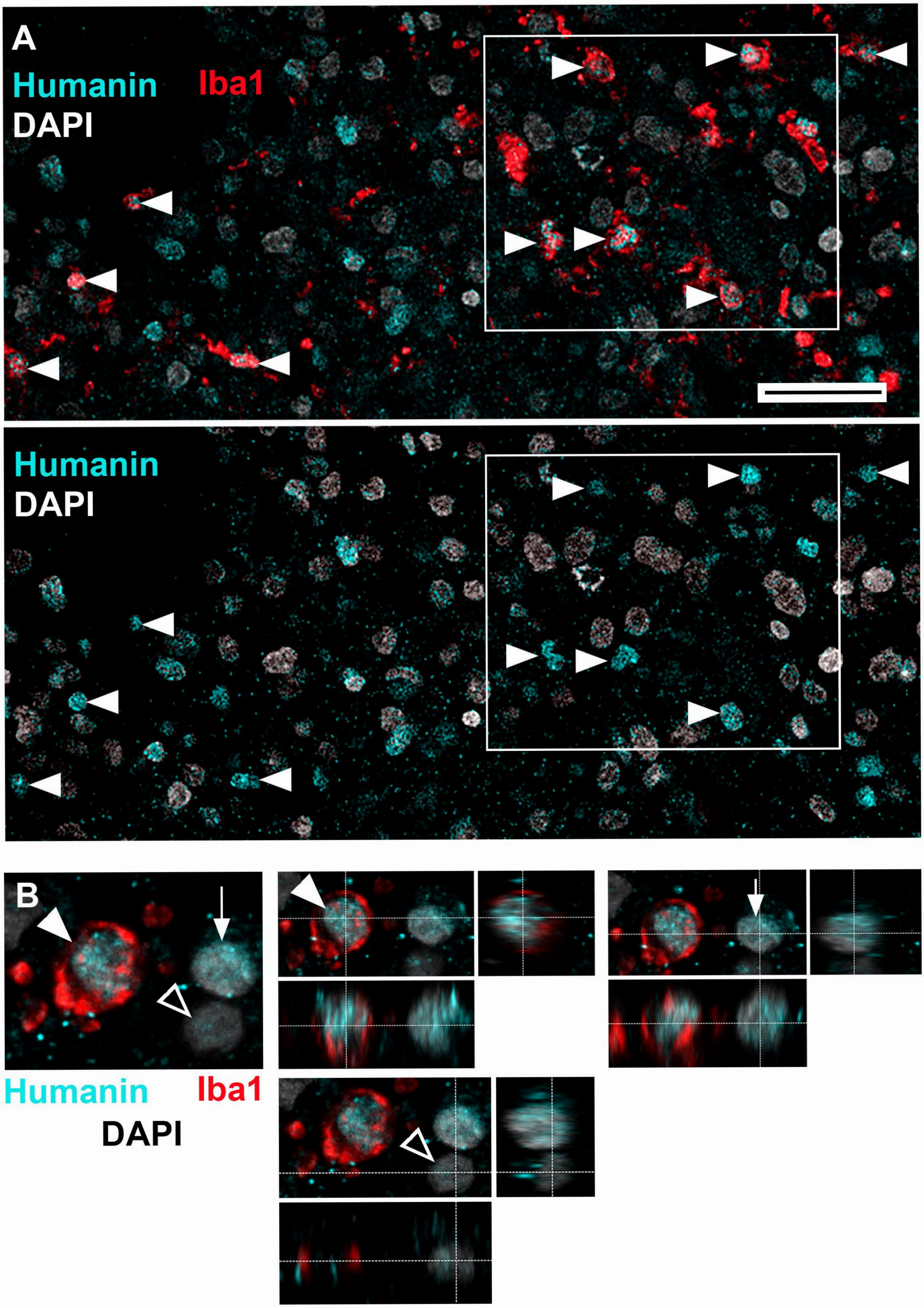

A
Humanin
Iba1
DAPI
Humanin
DAPI
B
Humanin
Iba1
DAPI

Figure S3: Humanin is broadly expressed in GBM associated myeloid cells (GAM) of human GBM specimen. Related to Figure 1. (**A)** Confocal micrograph from a patient specimen (nuclei in gray; size is representative for a quarter counting frame in our immunoquantification procedure, relating to Figure S2) co-immunostained for Humanin (cyan) and Iba1 (red). Cells double-positive for Humanin and Iba1 are marked by arrowheads; the insets correspond with Figure 1C. (**B)** This single optical section shows a representative example of an individual GAM (white arrowhead) co-stained for Humanin (cyan) and Iba1 (red) and an intra-tumoral cell (arrow) positive for Humanin only; furthermore, a cell (nucleus in grey) that is negative for Humanin or Iba1 (black arrowhead) is presented. Confocal cross hair inspection of optical Z-stacks confirmed Humanin expression in Iba1-positive GAM (arrowhead), Humanin single positive cells (arrow) or absence of Humanin/Iba1 in the cell marked with a black arrowhead .Scales bars indicate 50 µm (**A**), or 10 µm (**B**).

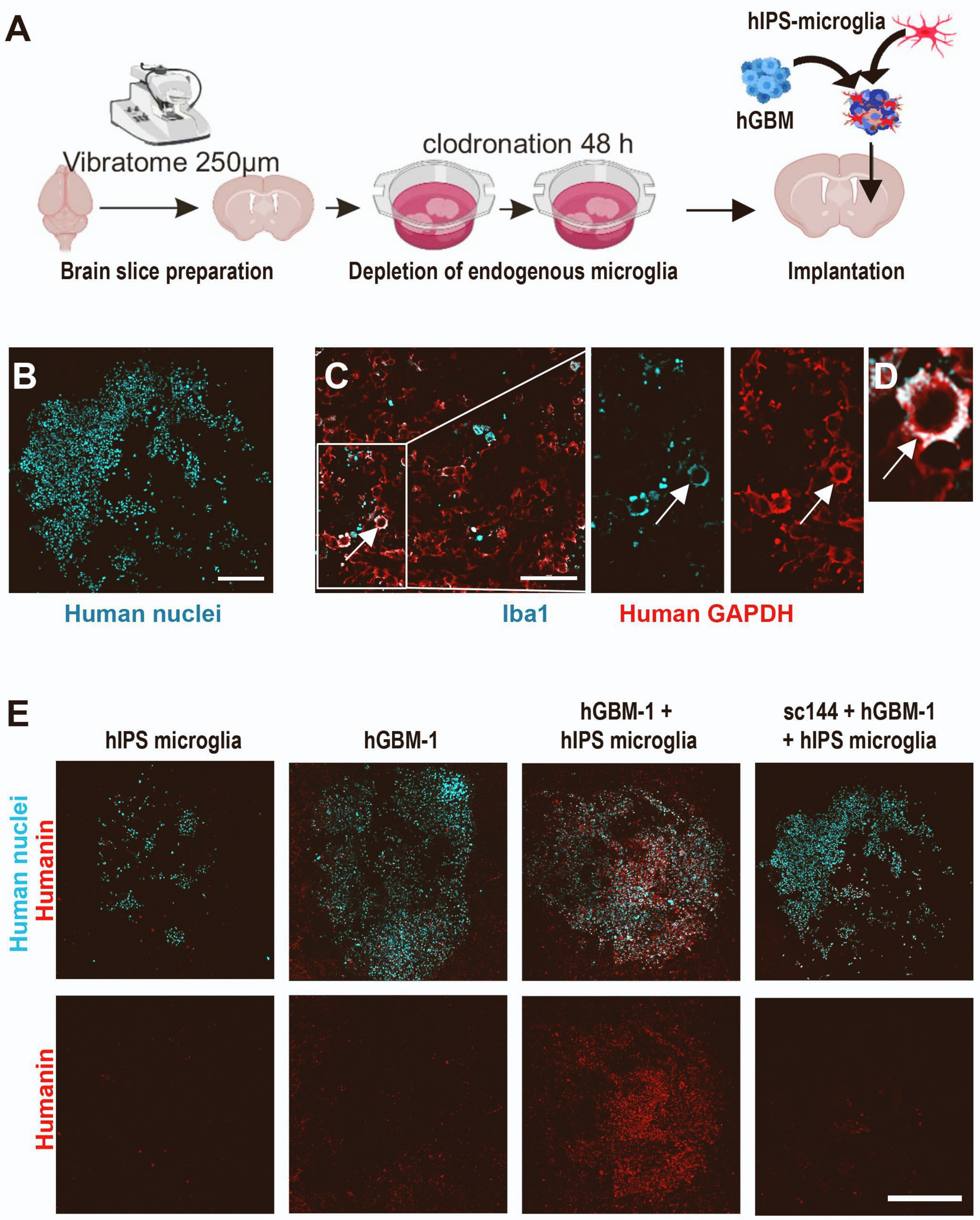
A
hIPS-microglia
hGBM
Vibratome 250µm
clodronation 48 h
Brain slice preparation
Depletion of endogenous microglia
Implantation
B
C
D
Human nuclei
Iba1
Human GAPDH
E
hIPS microglia
hGBM-1
hGBM-1 + hIPS microglia
sc144 + hGBM-1 + hIPS microglia
Human nuclei
Humanin
Humanin

Figure S4: The humanized brain slice model for GBM shows GP130 dependent Humanin expression upon GAM and GBM cell interaction. Related to Figures 2 and 3. (A) Schematic summary for the procedure to establish the humanized microglia ex vivo model. Briefly, brains from P14 mice were cut into 250 µm slices using a vibratome and mounted on inserts containing tissue culture medium. Clodronate filled liposomes were added into the medium for 48 h to deplete intrinsic murine microglia. Subsequently liposomes were washed away with normal medium and slices were cultured for 5 - 7 days. Then hIPS-microglia and glioma cells were mixed (1:1), co-inoculated into the organotypic brain slices and cultured for 5 days. Subsequently slices were fixed and the tumor volume was quantified. (**B)** Human cells in the brain slice culture system were readily detectable by immunofluorescence for human nuclei (cyan) or human-specific GAPDH (red). (**C-D**) The macrophage marker Iba-1 (cyan) was used to label hIPS-microglia on the organotypic brain slices, and differentiate human microglia from hGBM cells (red). The highlighted area in (**C**) was magnified, single channel recordings were provided and a single macrophage (arrow) is shown at high resolution (**D**). (**E**) For the analysis of Humanin expression in organotypic slices immunofluorescence co-staining for Humanin (red) and human nuclei (cyan) was performed and confocal tile scans of the entire tumor area were obtained. Humanin expression was upregulated when hIPS-microglia were present (see quantitative results in main figure 2a). Additional treatment of organotypic brain slices with GP130 antagonist sc144 during tumor growth reduced Humanin expression. Scale bar is 250 µm in (**B**), 100 µm in (**C**) and 500 µm in (**E**).

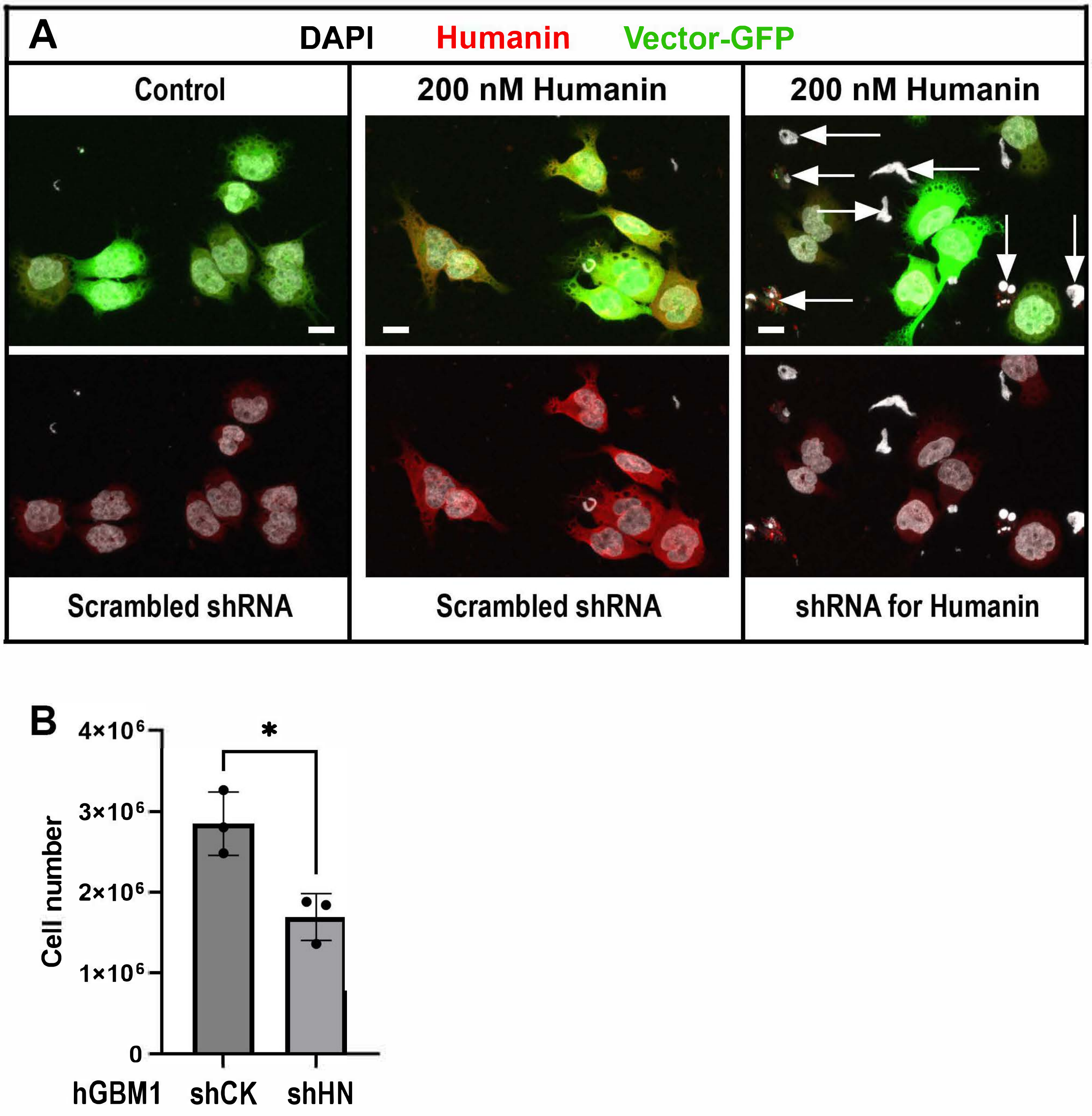
A
DAPI
Humanin
Vector-GFP
Control
200 nM Humanin
200 nM Humanin
Scrambled shRNA
Scrambled shRNA
shRNA for Humanin
B
Cell number
4×10⁶
3×10⁶
2×10⁶
1×10⁶
0
*
hGBM1
shCK
shHN

Figure S5: Expression of a Humanin knockdown constructs deteriorates GBM cell viability. Related to Figures 2 and 3. To investigate the usability of a Humanin knockdown vector we transduced GBM cells with lentiviral particles encoding a Humanin shRNA (hGBM-1_shHN) or a scrambeled shRNA control (hGBM-1_shCK) and analyzed by immunofluorescence against Humanin or tested for cell viability by cell counting. (**A**) Upon stimulation with exogenous 200 nM HN peptide, endogenous HN expression levels (red) increased in hGBM1 cells (DAPI labelled nuclei in grey) transduced with scrambled shRNA (positively labelled in green by Vector-GFP expression). In contrast, when hGBM-1 cells were treated with specific shRNA for Humanin endogenous Humanin expression levels after HN peptide application were reduced. (**B**) Quantification of cell numbers 5 days after shRNA transduction shows a significant decrease upon knockdown of endogenous Humanin (hGBM-1_shCK) as compared to scrambled shRNA treatment (of hGBM-1_shHN). Statistical significance is shown by ttest (*$p < 0.05$); scale bar is 10 µm.

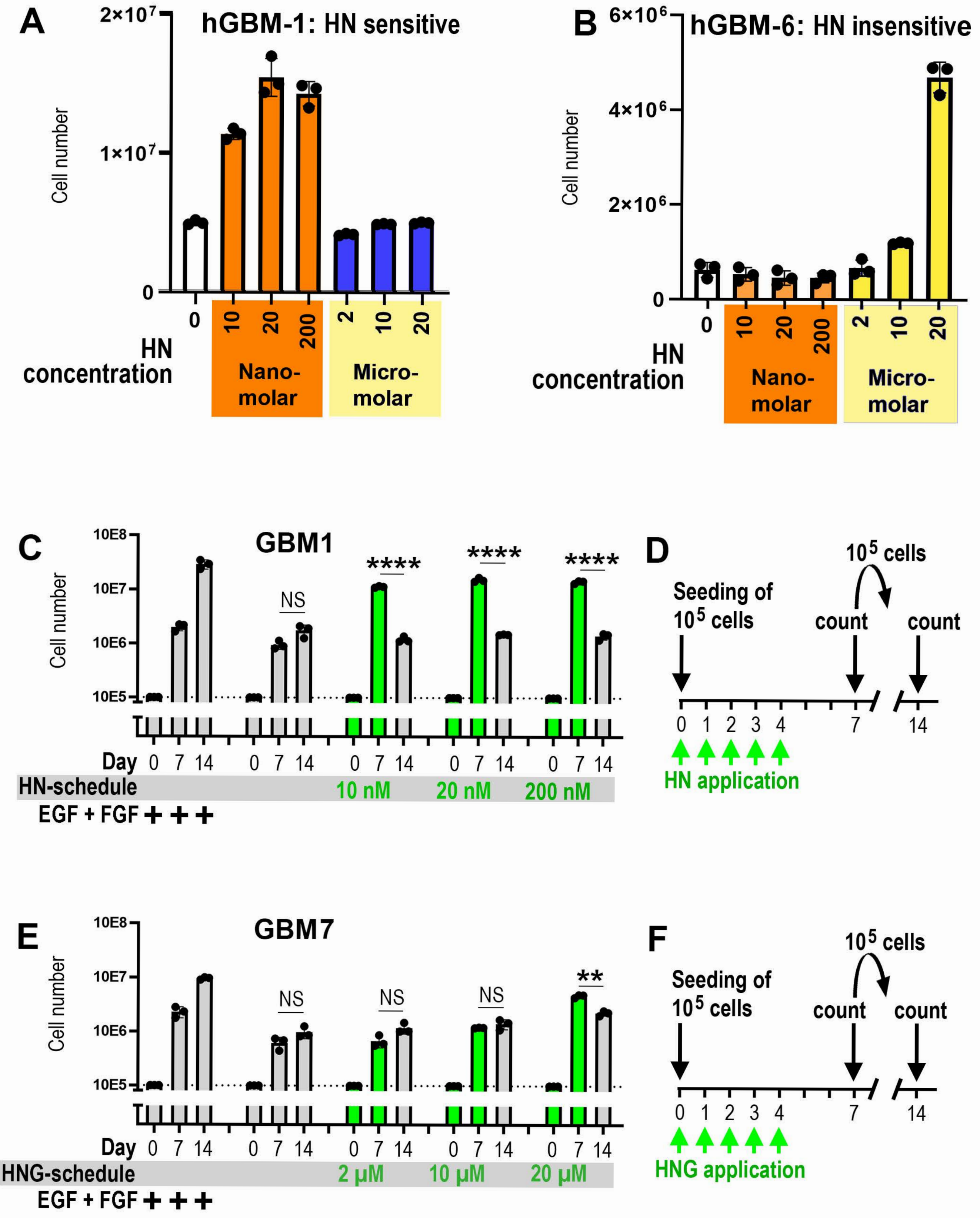
A
hGBM-1: HN sensitive
Cell number
2×10^7
1×10^7
0
0
10
20
200
2
10
20
HN concentration
Nano-molar
Micro-molar
B
hGBM-6: HN insensitive
Cell number
6×10^6
4×10^6
2×10^6
0
0
10
20
200
2
10
20
HN concentration
Nano-molar
Micro-molar
C
GBM1
Cell number
10E8
10E7
10E6
10E5
NS
****
****
****
Day 0 7 14 0 7 14 0 7 14 0 7 14 0 7 14
HN-schedule
10 nM
20 nM
200 nM
EGF + FGF + + +
D
Seeding of 10^5 cells
10^5 cells
count
count
0 1 2 3 4 7 14
HN application
E
GBM7
Cell number
10E8
10E7
10E6
10E5
NS
NS
NS
**
Day 0 7 14 0 7 14 0 7 14 0 7 14 0 7 14
HNG-schedule
2 µM
10 µM
20 µM
EGF + FGF + + +
F
Seeding of 10^5 cells
10^5 cells
count
count
0 1 2 3 4 7 14
HNG application

Figure S6: Humanin is a potent inducer of chemoresistance in Humanin insensitive GBM. Related to Figures 2 and 3. (**A, B**) hGBM cells were treated with nanomolar to micromolar concentrations of Humanin (HN) peptide daily and cells were counted after 14 days. HN-sensitive hGBM-1 cells showed increased expansion after treatment with nanomolar (but not micromolar) HN concentrations (as compared to Humanin-free controls, "0"). In contrast, HN-insensitive hGBM-6 cells showed increased expansion only after addition of 20 µM HN. (**C-F**) hGBM (10^5) cells were seeded, maintained in medium with EGF/FGF (controls) or in growth factor free medium (Ctrl.); cells under growth factor free conditions were stimulated with different concentrations of HN or Humanin-G (HNG) and counted 7 days later, then the same cells were used to repeat the procedure without addition of HN or HNG; note that 20 nM HN is sufficient to promote expansion of Humanin sensitive hGBM1, whereas 20 µM HNG were required to support the growth of the Humanin insensitive hGBM7 cells; in both cases the pharmacological effects were transient (ceased when HN/HNG were omitted after re-plating); statistical significance was assessed by One-Way-ANOVA: $^{**}p < 0.01$, $^{****}p < 0.0001$.

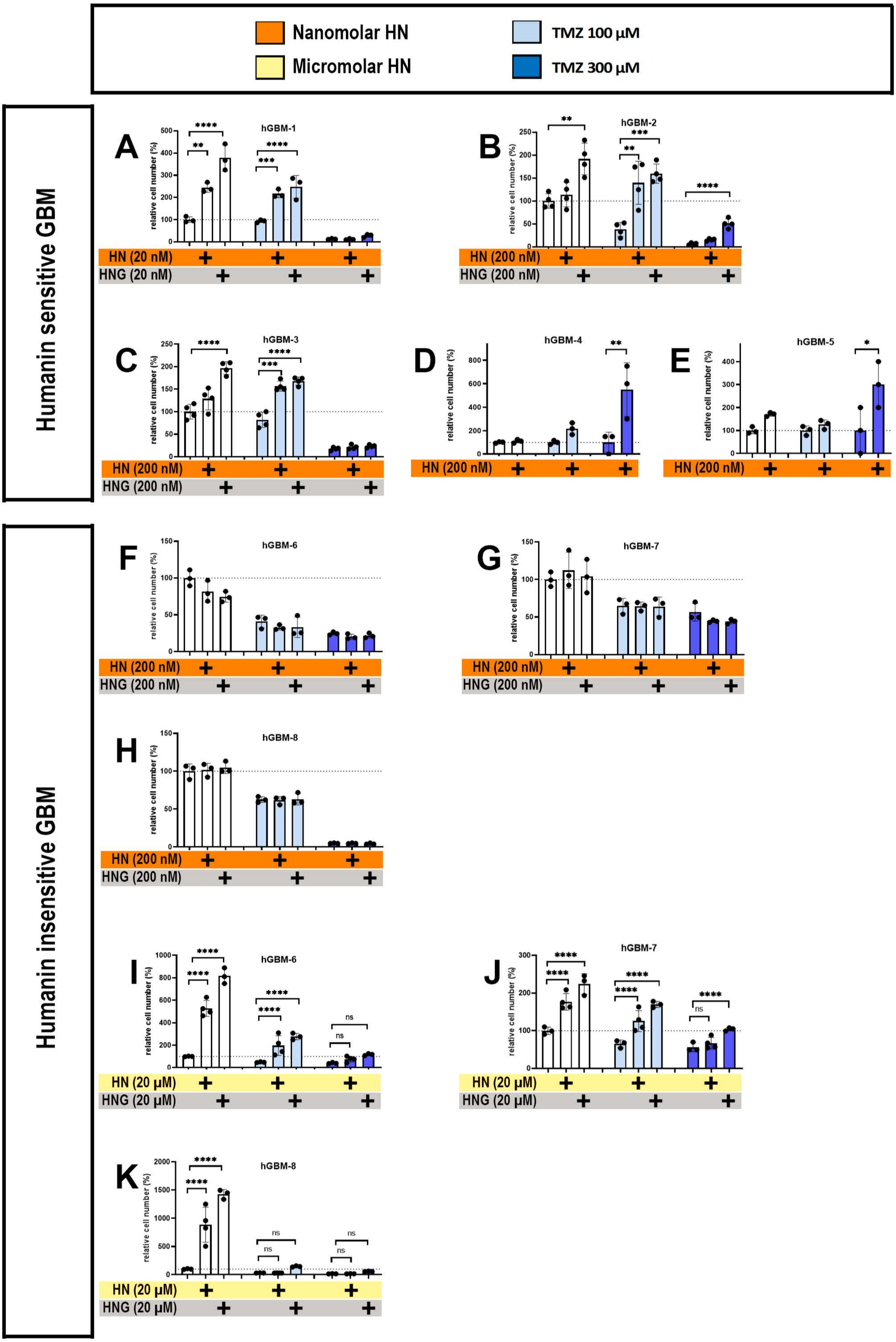

Nanomolar HN
Micromolar HN
TMZ 100 μM
TMZ 300 μM
Humanin sensitive GBM
Humanin insensitive GBM
A
hGBM-1
B
hGBM-2
C
hGBM-3
D
hGBM-4
E
hGBM-5
F
hGBM-6
G
hGBM-7
H
hGBM-8
I
hGBM-6
J
hGBM-7
K
hGBM-8
relative cell number (%)
HN (20 nM)
HNG (20 nM)
HN (200 nM)
HNG (200 nM)
HN (20 μM)
HNG (20 μM)
ns

Figure S7: Humanin promotes chemoresistance at nanomolar concentrations in Humanin-senstive GBM. Related to Figures 2 and 3. To compare cell expanssion and chemoresistance induced by Humanin, hGBM cells were exposed to different concentrations of the chemotherapeutic temozolomide (TMZ) and partially supplemented with nanomolar (**A-H**) or micromolar (**I-K**) concentration of Humanin (HN) or the pharmacologically potent Humanin mutant HNG. Cell numbers were quantified after 14 days of treatment; and were normalized to untreated control samples. (**A-E**) HN-sensitive hGBM1 - hGBM5 resisted treatment with 100 µM (A-C) or 300 µM (D, E) TMZ when nanomolar amounts of HN (or HNG) were coapplied. (**F-H**) HN-insensitive hGBM6 - hGBM8 were not resistant to TMZ when nanomolar HN (or HNG) concentrations were applied. (**I, J**), Chemoresistance of Humanin insensitive hGBM6 or hGBM7 was observed after addition of micromolar concentrations of HN (or HNG). (**K**) In hGBM-8 even high micromolar concentrations of HN or HNG did not induce TMZ resistance; statistical significance was assessed by One-Way-ANOVA: $^{**}p < 0.01$, $^{***}p < 0.001$, $^{****}p < 0.0001$.

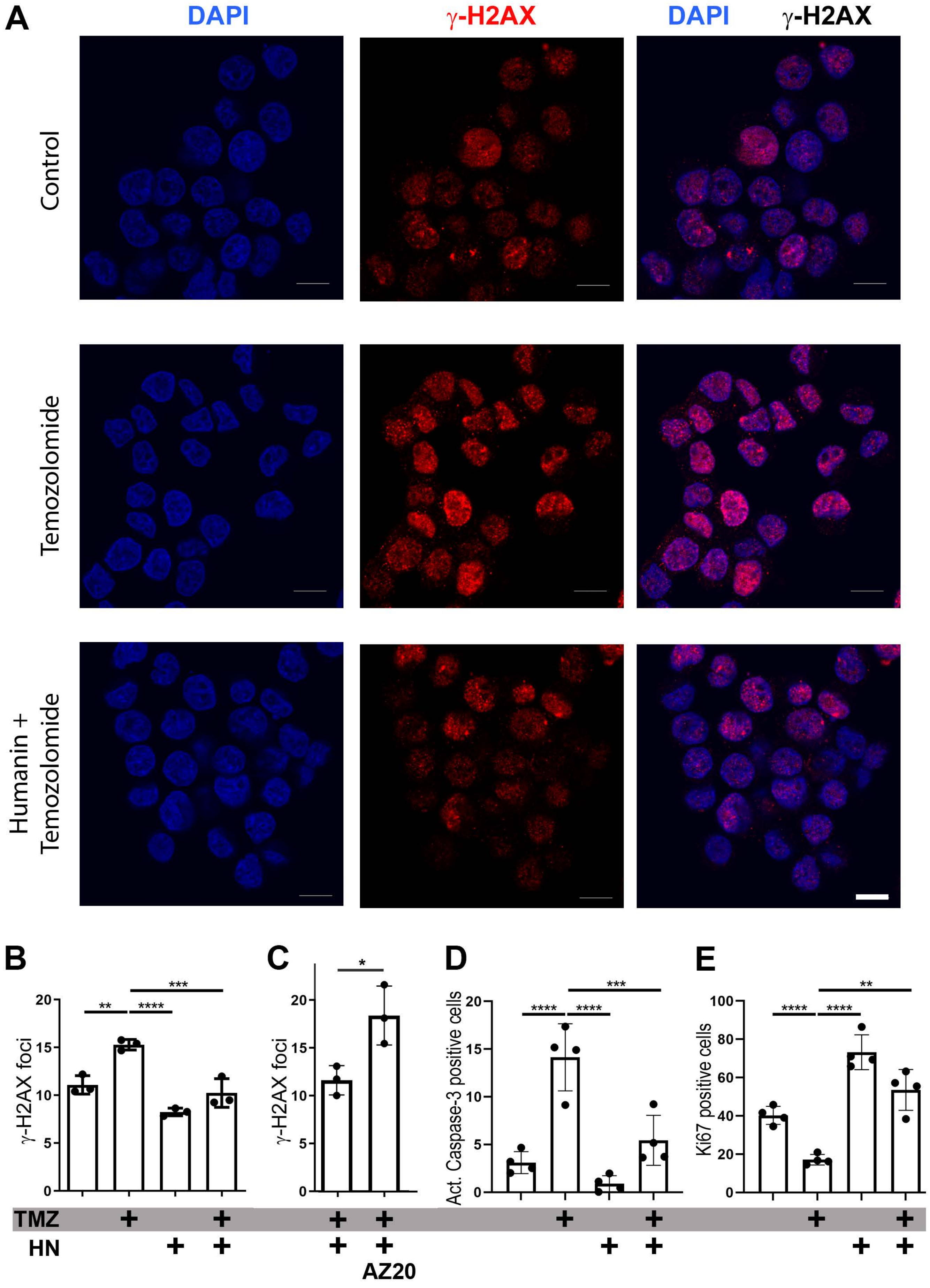
A
DAPI
γ-H2AX
DAPI γ-H2AX
Control
Temozolomide
Humanin + Temozolomide
B
γ-H2AX foci
C
γ-H2AX foci
D
Act. Caspase-3 positive cells
E
Ki67 positive cells
TMZ
HN
AZ20

Figure S8: Humanin promotes chemoresistance through DNA damage repair. Related to Figures 2 and 3. (**A, B**) GBM cells were left untreated (control) or were treated with Humanin (HN, 100 nM) or TMZ (100 µM) alone or in combination. Cells were stained for γH2AX as marker for DNA double-strand breaks (red) and with DAPI for nuclear labeling (grey). **(A)** TMZ treatment increased nuclear labeling for γH2AX (as expected) and this was blunted by coapplication with HN. (**B**) Quantification of the immunofluorescence labeling shown in (A). (**C**) Addition of the ATR inhibitor AZ20 to TMZ and HN treated cells attenuated DNA repair (increased nuclear γH2AX foci). (**D**) Immunostaining for activated-Caspase-3 was performed and apoptotic cells were counted. TMZ increased cell apoptosis significantly, while co-treament with HN led to near complete protection from TMZ-induced apoptosis. (**E**) Counting of Ki67-positive cell showed a significant reduction in cell cycle entry in TMZ treated GBM, compared to untreated controls. HN blunted the anti-proliferative effect of TMZ. Statistical significance was assessed by One-Way-ANOVA: $*p < 0.05$, $**p < 0.01$, $***p < 0.001$, $****p < 0.0001$; scale bar is 10 µm.

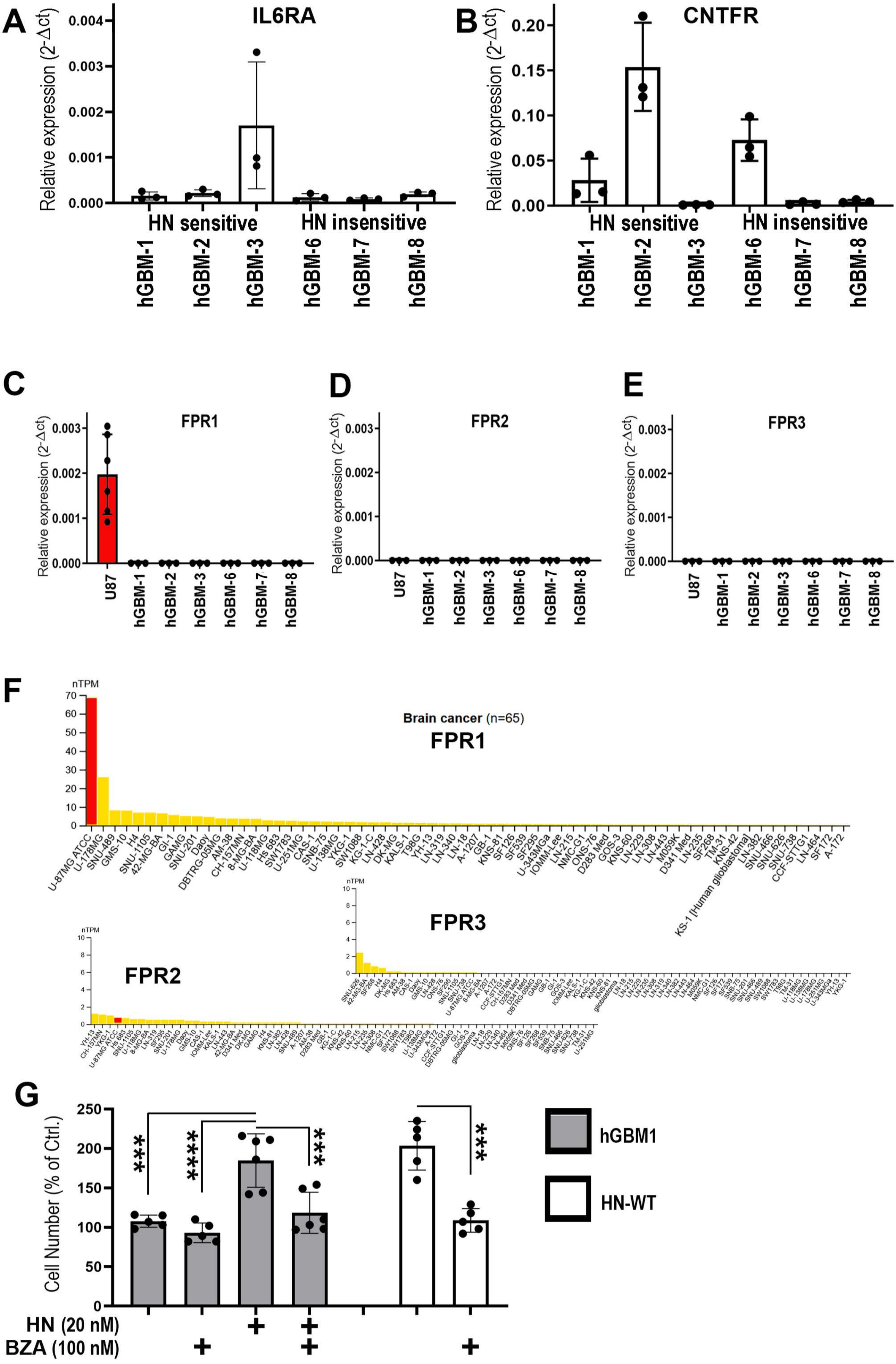

A
IL6RA
Relative expression (2-Δct)
0.004
0.003
0.002
0.001
0.000
HN sensitive
HN insensitive
hGBM-1
hGBM-2
hGBM-3
hGBM-6
hGBM-7
hGBM-8
B
CNTFR
Relative expression (2-Δct)
0.20
0.15
0.10
0.05
0.00
HN sensitive
HN insensitive
hGBM-1
hGBM-2
hGBM-3
hGBM-6
hGBM-7
hGBM-8
C
FPR1
Relative expression (2-Δct)
0.003
0.002
0.001
0.000
U87
hGBM-1
hGBM-2
hGBM-3
hGBM-6
hGBM-7
hGBM-8
D
FPR2
Relative expression (2-Δct)
0.003
0.002
0.001
0.000
U87
hGBM-1
hGBM-2
hGBM-3
hGBM-6
hGBM-7
hGBM-8
E
FPR3
Relative expression (2-Δct)
0.003
0.002
0.001
0.000
U87
hGBM-1
hGBM-2
hGBM-3
hGBM-6
hGBM-7
hGBM-8
F
nTPM
70
60
50
40
30
20
10
0
Brain cancer (n=65)
FPR1
U-87MG ATCC
U-178MG
SNU-489
GMS-10
H4
SNU-1105
42-MG-BA
GI-1
GAMG
SNU-201
Daoy
DBTRG-05MG
AM-38
CH-157MN
8-MG-BA
U-118MG
Hs 683
SW1783
U-251MG
CAS-1
SNB-75
U-138MG
YKG-1
SW1088
KG-1-C
LN-428
DK-MG
KALS-1
T98G
YH-13
LN-319
LN-340
LN-18
A-1207
GB-1
KNS-81
SF126
SF539
SF295
U-343MGa
IOMM-Lee
LN-215
NMC-G1
ONS-76
D283 Med
GOS-3
KNS-60
LN-229
LN-308
LN-443
M059K
D341 Med
LN-235
SF268
TM-31
KNS-42
KS-1 [Human glioblastoma]
LN-382
SNU-466
SNU-626
SNU-738
CCF-STTG1
LN-464
SF172
A-172
nTPM
10
8
6
4
2
0
FPR3
nTPM
10
8
6
4
2
0
FPR2
G
Cell Number (% of Ctrl.)
250
200
150
100
50
0
***
****
***
***
hGBM1
HN-WT
HN (20 nM)
BZA (100 nM)

Figure S9: Expression and effect of GP130 in GBM. Related to Figure 3. (**A-B**) Quantitative RT-PCR of Humanin receptor subunits was performed in Humanin sensitive and insensitive GBM; note that a component of interleukin receptor (Il6RA) was detectable in Humanin sensitive GBM; CNTFR expression levels partly were low in both Humanin sensitive and insensitive hGBM, whereas other putative human receptors (FPR1, FPR2 or FPR3) were not detected in any hGBM (**C-E**). U87 cells (in C; red bar) served as a positive control for FPR1 detection in agreement with data from the Human Protein Atlas (HPR); HPR-derived FPR expression profiles from GBM cell lines are presented in (**F**); congruent with our qPCR data FPR1 (but not FPR2 or FPR3) is detectable in U87 cells (red bar). (**G**) The GBM cell proliferative effect of Humanin is conferred through GP130. hGBM-1, were stimulated by the addition of HN peptide (20 nM) or by HN overexpression (in HN-WT transfected hGBM-1) and part of the cells were challenged with the GP130 inhibitor bazedoxifene-A (BZA; 100 nM), which consistently abrogated the protumorigenic effect of HN as assessed by cell counting. Statistical significance is shown by One-Way-ANOVA in (***p < 0.001; ****p < 0.0001).

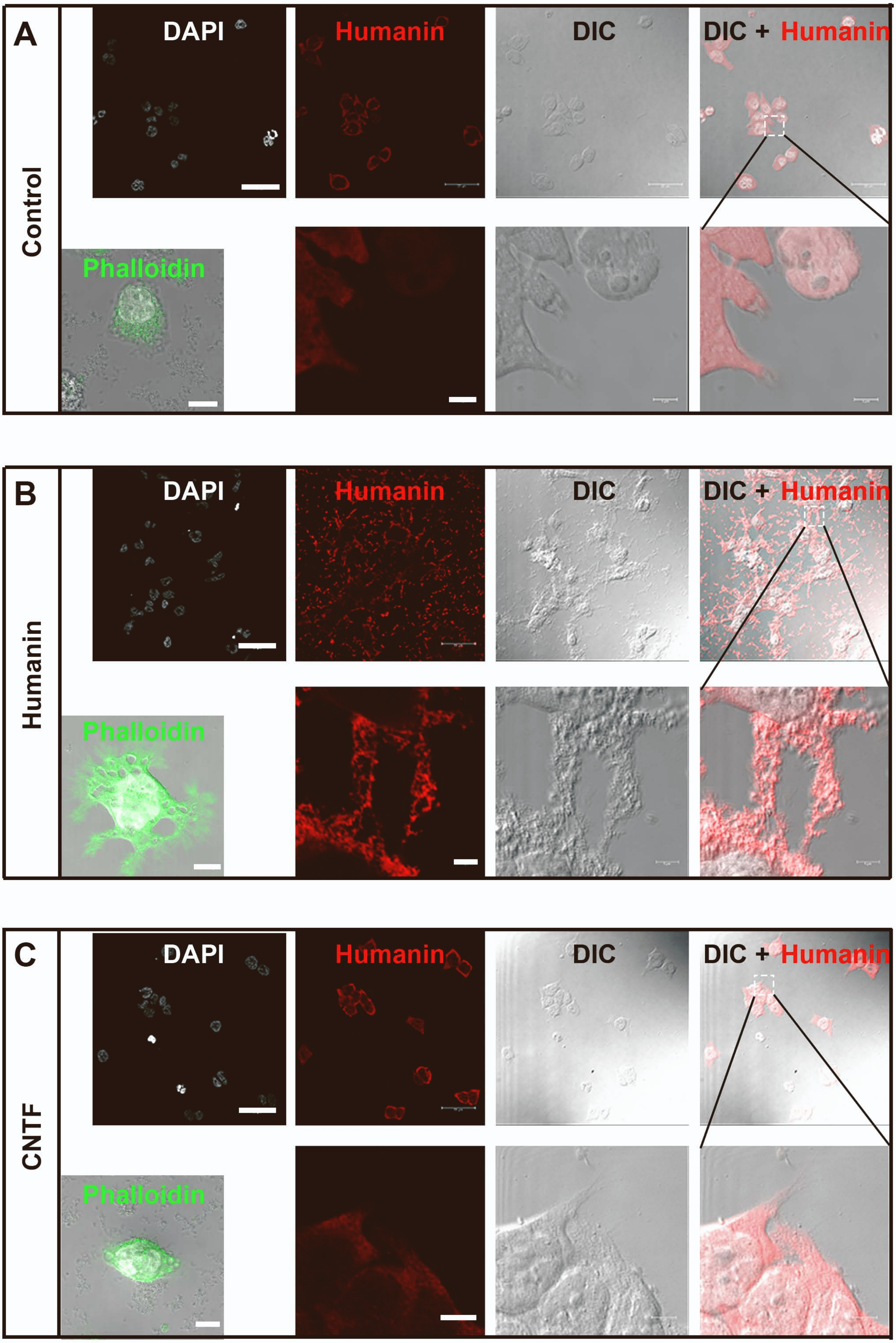
A
B
C
Control
Humanin
CNTF
DAPI
Humanin
DIC
DIC + Humanin
Phalloidin

Figure S10: Exogenous Humanin peptide induces Humanin expression in GBM. Related to Figure 3. GBM cells were treated with GP130 ligands for 20 hours (or were left untreated; control), the cytoskeleton was stained with Phalloidin (green) and nuclei with DAPI (grey) before fixation and immunostaining against Humanin (HN). (**A**-**C**) Confocal single channel recordings for DAPI (nuclei, grey), Humanin (red), F-Actin (green) and Differential Interference Contrast (DIC) are shown next overlay images; image parts are shown at higher magnification. (**A**) Untreated control cells manifest with weak Humanin signal and rounded cell appearance. (**B**) Cells treated with 200 nM HN peptide show a strong increase in intracellular Humanin expression instead, and a strong morphological change is observed (by DIC). In the overlay (DIC + Humanin) the HN-treated cells show large cell protrusions (held by Phalloidin-positive F-Actin filaments) that are full of endogenous Humanin. The GP130 ligand CNTF (**C**) ,100 ng/ml, neither changes Humanin expression levels nor cell morphology in the concentrations applied compared to untreated control (A). Scale bars are 10 µm (overview) or 1 µm (magnified).

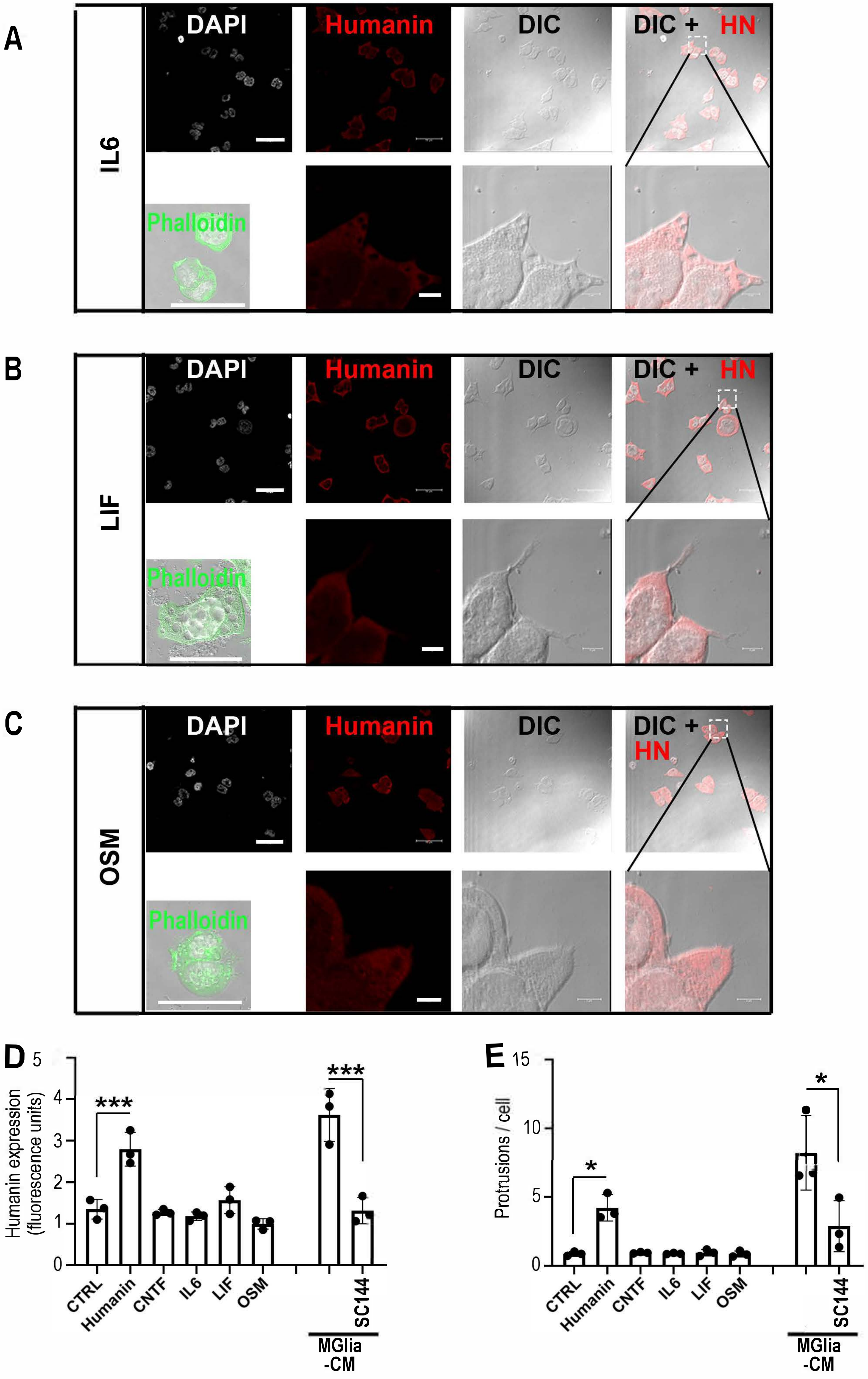

A
IL6
DAPI
Humanin
DIC
DIC + HN
Phalloidin
B
LIF
DAPI
Humanin
DIC
DIC + HN
Phalloidin
C
OSM
DAPI
Humanin
DIC
DIC + HN
Phalloidin
D
Humanin expression (fluorescence units)
0
1
2
3
4
5
***
***
CTRL
Humanin
CNTF
IL6
LIF
OSM
SC144
MGlia -CM
E
Protrusions / cell
0
5
10
15
*
*
CTRL
Humanin
CNTF
IL6
LIF
OSM
SC144
MGlia -CM

Figure S11: Exogenous Humanin peptide induces Humanin expression in GBM. Related to Figure 3. GBM cells were treated with GP130 ligands for 20 hours (or were left untreated; control), the cytoskeleton was stained with Phalloidin (green) and nuclei with DAPI (grey) before fixation and immunostaining against Humanin (HN). Activation of GP130 with 100 ng/ml IL6 (**A**), 10 ng/ml LIF (**B**), or 100 ng/ml OSM (**C**) neither changes Humanin expression levels nor cell morphology in the concentrations applied compared to untreated control (Fig. S10, A). Scale bar are 10 µm (overview) or 1 µm (magnified). (**D**) The integrated fluorescence values of Humanin staining were quantified showing a significant increase only in Humanin treated GBM (and not with other GP130 ligands) compared to negative control. Note that the incubation of hGBM cells with microglia-conditioned medium (MGlia-CM) induces a comparable significant increase in Humanin expression which was fully blocked by GP130 inhibitor sc144. (**E**) Protrusions per cell were counted and show a significantly higher number in Humanin-treated cells compared to controls or the other classical GP130 ligands. Again, this effect was copied by MGlia-CM addition and fully blockable by sc144. Scale bars are 10 µm (overview) or 1 µm (magnified). Experiments were conducted in triplicates and statistical significance was assessed by One-Way-ANOVA: *$p < 0.05$, ****$p < 0.0001$.

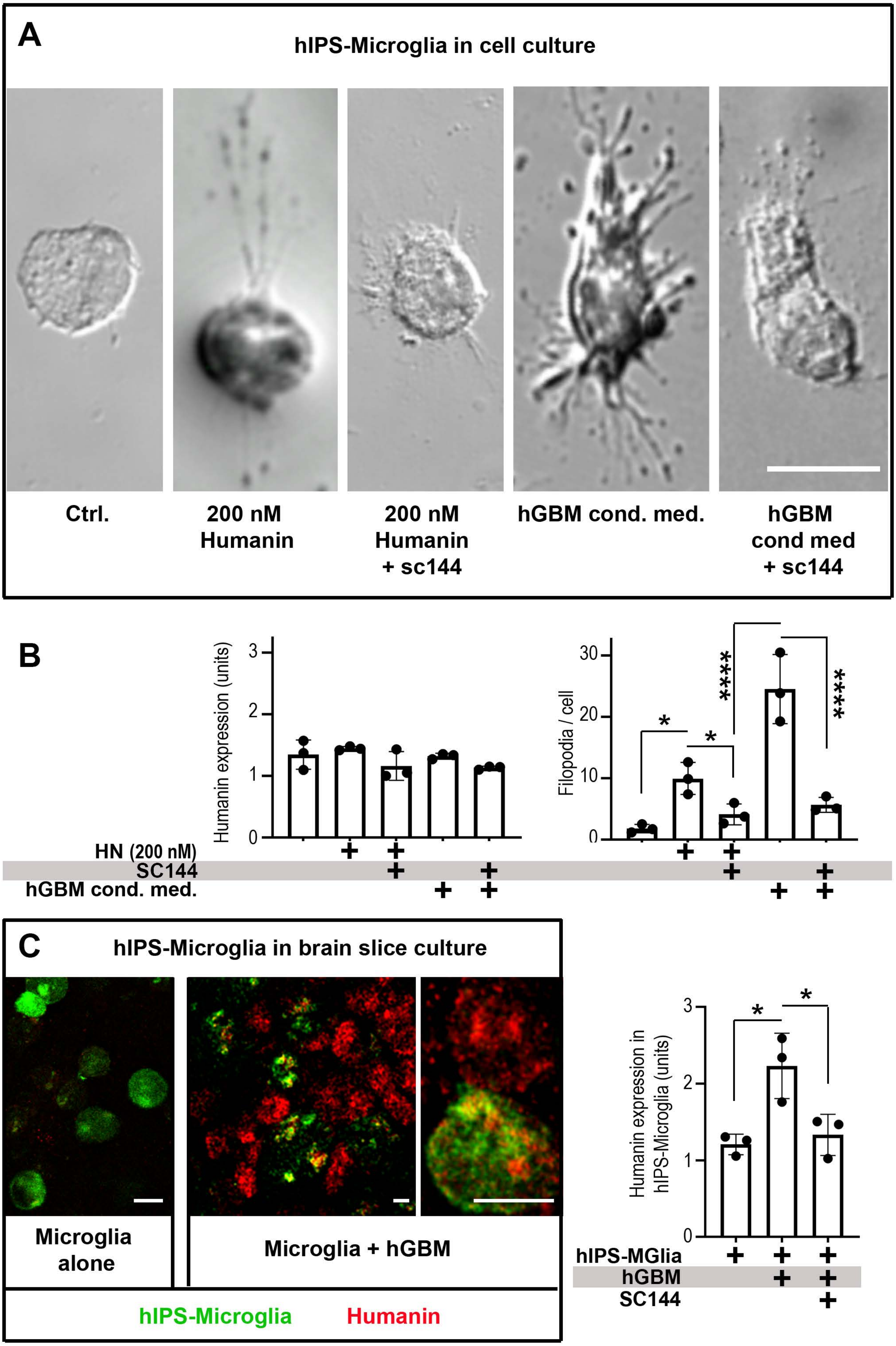
A
hIPS-Microglia in cell culture
Ctrl.
200 nM Humanin
200 nM Humanin + sc144
hGBM cond. med.
hGBM cond med + sc144
B
Humanin expression (units)
Filopodia / cell
HN (200 nM)
SC144
hGBM cond. med.
C
hIPS-Microglia in brain slice culture
Microglia alone
Microglia + hGBM
hIPS-Microglia
Humanin
Humanin expression in hIPS-Microglia (units)
hIPS-MGlia
hGBM
SC144

Figure S12: GBM cell interaction induces Humanin expression in hIPS-microglia in a GP130 dependent manner. Related to Figure 3. (A, B) Human microglia-like cells from induced pluripotent stem cells (hiPS-microglia) were left untreated or were treated with Humanin (200 nM), hGBM conditioned medium alone or in combination with GP130 inhibitor sc144. (**A**) DIC-micrograph of representative cells are shown in high magnifications. Upon Humanin treatment (but not under control conditions) hIPS-mcroglia extend protrusions (note that Humanin treatment increases cell volume and thereby changes opacity in DIC imaging). Application of hGBM- conditioned medium induces a strong morphological change in microglia showing many protrusions, which is blunted by coapplication of sc144. (**B**) Humanin expression does not change in treated versus control cells; the number of filopodia observed per cell (as compared to controls) significantly increases upon Humanin treatment or when hGBM-conditioned medium is applied; both effects are attenuated by sc144. (**C**) In brain slice cultures Humanin expression was analyzed in slices containing Microglia alone or upon interaction of Microglia (GFP+) with hGBM cells. Humanin immunostaining (in red) is increased in slices containing both hIPS-microglia and GBM cells (as compared to slices containing solely microglia). Microglial immunolabeling for Humain was quantified under the indicated experimental conditions. Scale bar are 10 µm. Experiments were conducted in triplicates and statistical significance was assessed by One-Way-ANOVA: *$p < 0.05$, ****$p < 0.0001$.

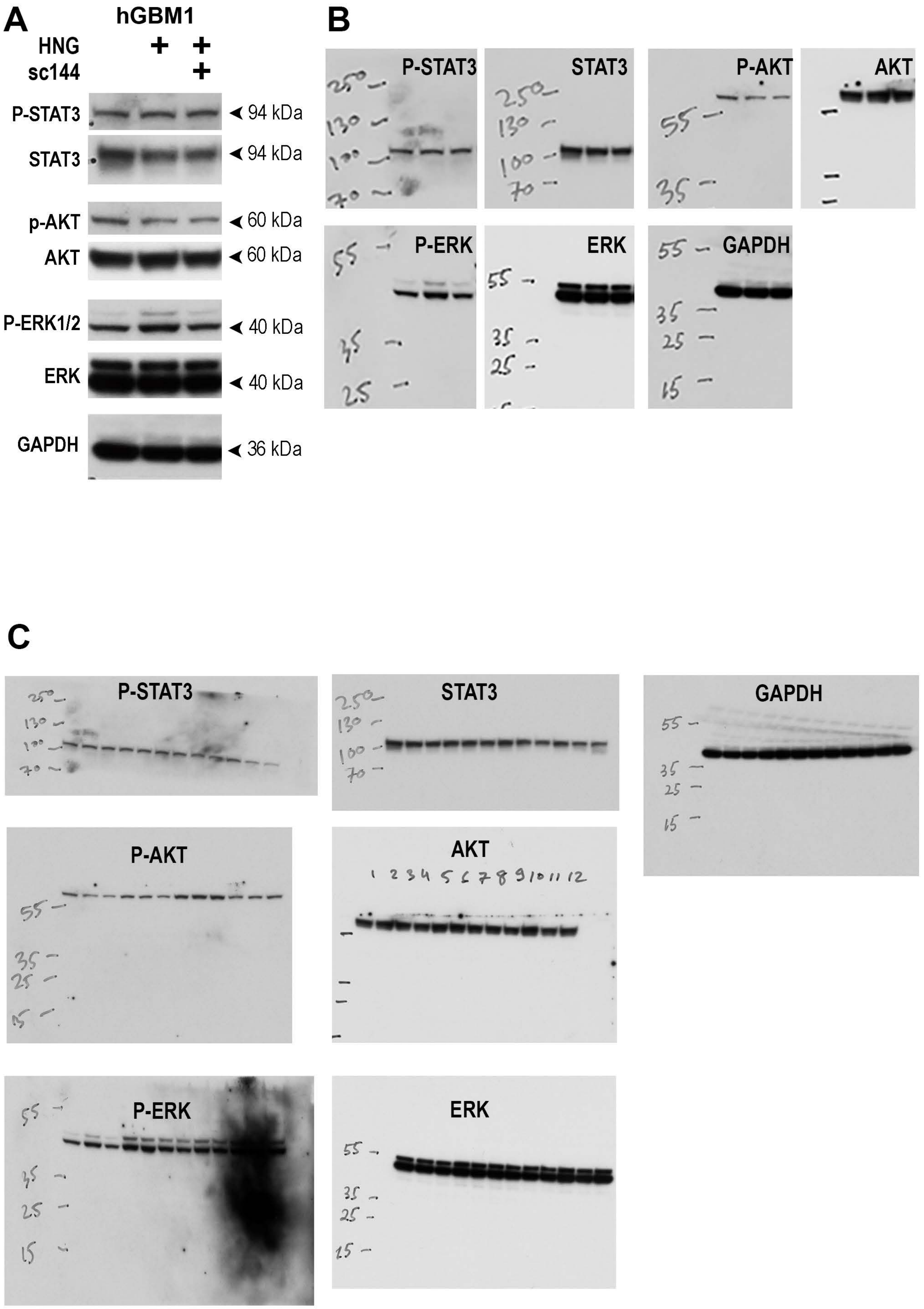
A
hGBM1
HNG
sc144
P-STAT3
STAT3
p-AKT
AKT
P-ERK1/2
ERK
GAPDH
94 kDa
94 kDa
60 kDa
60 kDa
40 kDa
40 kDa
36 kDa
B
P-STAT3
STAT3
P-AKT
AKT
P-ERK
ERK
GAPDH
C
P-STAT3
STAT3
GAPDH
P-AKT
AKT
P-ERK
ERK

Figure S13: HNG stimulates ERK-signaling through GP130 activation. Related to Figures 3 and 4. (A) hGBM1 cells were stimulated with HNG (20 nM for 16h) or were left untreated, cell pellets were harvested and used for Western blotting; note that HNG strongly induced ERK1/2 activation (phosphorylation of ERK1/2), while STAT3 was already active under control conditions and not further activated by HNG. (**B-C**) Western blotting membranes showing the selected lanes (**B**) and the complete membranes (**C**) corresponding with the cropped bands shown in (**A**) are presented.

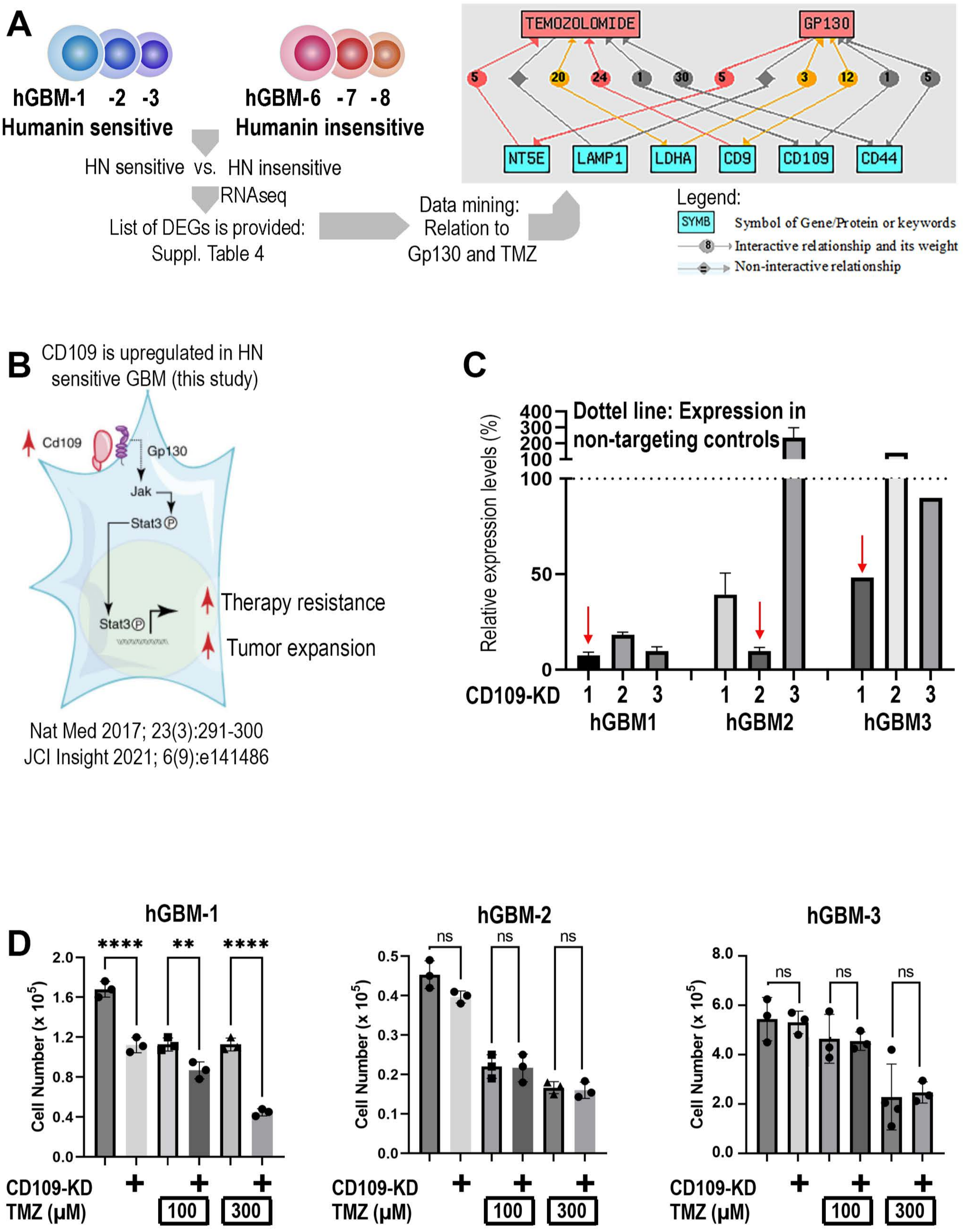
A
hGBM-1 -2 -3
Humanin sensitive
hGBM-6 -7 -8
Humanin insensitive
HN sensitive vs. HN insensitive
RNAseq
List of DEGs is provided:
Suppl. Table 4
Data mining:
Relation to
Gp130 and TMZ
TEMOZOLOMIDE
GP130
NT5E
LAMP1
LDHA
CD9
CD109
CD44
Legend:
SYMB
Symbol of Gene/Protein or keywords
Interactive relationship and its weight
Non-interactive relationship
B
CD109 is upregulated in HN sensitive GBM (this study)
Cd109
Gp130
Jak
Stat3
Therapy resistance
Tumor expansion
Nat Med 2017; 23(3):291-300
JCI Insight 2021; 6(9):e141486
C
Dottel line: Expression in non-targeting controls
Relative expression levels (%)
CD109-KD
hGBM1
hGBM2
hGBM3
D
hGBM-1
hGBM-2
hGBM-3
Cell Number (x 10^5)
CD109-KD
TMZ (μM)
100
300
ns
****
**

Figure S14: Humanin sensitive GBM are characterized by a set of genetic markers. Related to Figure 5. (**A**) Humanin sensitive (hGBM-1, -2 and -3) as well as Humanin insensitive (hGBM-6, -7 and -8) cells were grown under control conditions (72h in EGF-, bFGF-free medium), underwent transcriptomics and differentially expressed genes (DEGs) were analyzed by bioinformatics. We hypothesized that DEGs enriched in Humanin sensitive hGBM should have a role in GP130 signaling and chemoresistance (to TMZ). Hence, we explored if this gene-set (genes upregulated in Humanin sensitive hGBM) had known relations with GP130 signaling and TMZ efficacy (using bioinformatics mining by http://www.chilibot.net/; as described in *Neuro Oncol 2021. 2;23(11): 1898-1910*). This showed that Humanin sensitive GBM are characterized by high expression levels of a range of molecules (NT5E, LAMP1, LDHA, CD9, CD109, CD44) relating to both TMZ-function and GP130 signaling. (**B**) CD109 is an interaction partner for GP130, which induces STAT3-activation (phosphorylation) independently from other ligands. This has pathological relevance for melanoma and GBM (see references). (**C**) Knockdown of CD109 (CD109-KD) in Humanin sensitive GBM (hGBM-1, -2 or-3) was quantified by qPCR (versus non-targeting controls; C) and hGBMs with CD109-KD were selected (arrow) for experiments in (D). (**D**) Controls and CD109-KD cells were maintained under control conditions or exposed to TMZ (100 or 300 µM), cell numbers were counted after 5 days. This showed that CD109-KD affected TMZ-resistance in hGBM1, but not in hGBM2. Hence, Humanin sensitive hGBM express a range of molecules relating to TMZ resistance and GP130 modulation. These molecules serve as markers to identify Humanin sensitive GBM, but do not always play a pivotal functional role, whereas Humanin signaling strongly and consistently promoted chemoresistance in this subset of GBM.

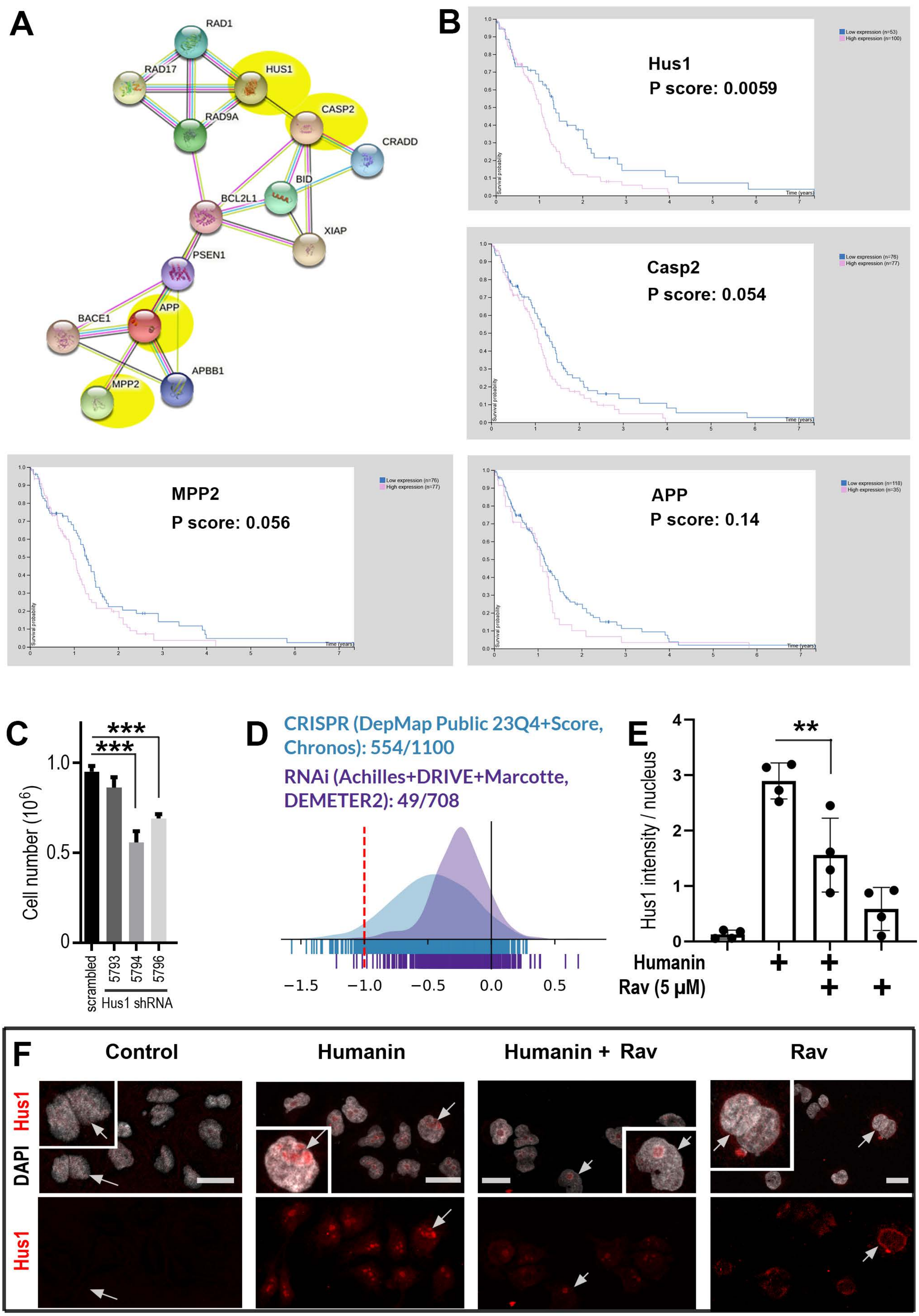
A
RAD1
RAD17
HUS1
RAD9A
CASP2
CRADD
BID
BCL2L1
XIAP
PSEN1
APP
BACE1
APBB1
MPP2
B
Hus1
P score: 0.0059
Casp2
P score: 0.054
MPP2
P score: 0.056
APP
P score: 0.14
Low expression (n=53)
High expression (n=100)
Low expression (n=76)
High expression (n=77)
Low expression (n=118)
High expression (n=35)
Survival probability
Time (years)
C
Cell number (10^6)
scrambled
5793
5794
5796
Hus1 shRNA
D
CRISPR (DepMap Public 23Q4+Score, Chronos): 554/1100
RNAi (Achilles+DRIVE+Marcotte, DEMETER2): 49/708
E
Hus1 intensity / nucleus
Humanin
Rav (5 μM)
F
Control
Humanin
Humanin + Rav
Rav
Hus1
DAPI
Hus1

Figure S15: Hus1 is a pathologically relevant, Humanin induced gene. Related to Figure 5. (**A**) hGBM-1, -2 and -3 cells were stimulated with HN or vehicle (Ctrl.), underwent transcriptomics and differentially expressed genes (DEGs) were analyzed by bioinformatics; note that that HN induced e.g. nuclear signaling pathways providing 12 consistent DEGs (Suppl. Table 5), of which several components (highlighted in yellow) assembled in a network (https://string-db.org/). (**B**) From the gene list obtained in (A) the DNA clamp component HUS1 showed strong association with survival in human GBMs (https://www.proteinatlas.org/), in contrast to other genes from the same cluster (marked in yellow in A). (**C**) hGBM-1 cells were treated by three different shRNAs for Hus1 and viability was assessed by cell counting. Cell viability decreased significantly compared to scrambled control shRNA treated cells. (**D**) Interrogation of the DepMap database suggests that HUS1 knockdown/knockout compromises cell viability. (**E-F**) hGBM-1 cells were stimulated with HN (200 nM) alone or in presence with ERK-inhibitor Ravoxertinib (RAV; 5 µM), with RAV alone or were left untreated. Cells were analyzed by immunofluorescence for HUS1 (red) using confocal microscopy. Cell nuclei are labelled by DAPI (grey). The fluorescent signal intensity was quantified by ImageJ. The increase in HUS1 expression observed after HN treatment is significantly reduced by RAV. Note that HUS1 shows perinuclear localization upon RAV treatment. The insets depict close-up views of cells marked by an arrow. Scale bar is 10 µm in. Statistical significance was assessed by One-Way-ANOVA: $^{**}p < 0.01$, $^{***}p < 0.001$, $^{****}p < 0.0001$.

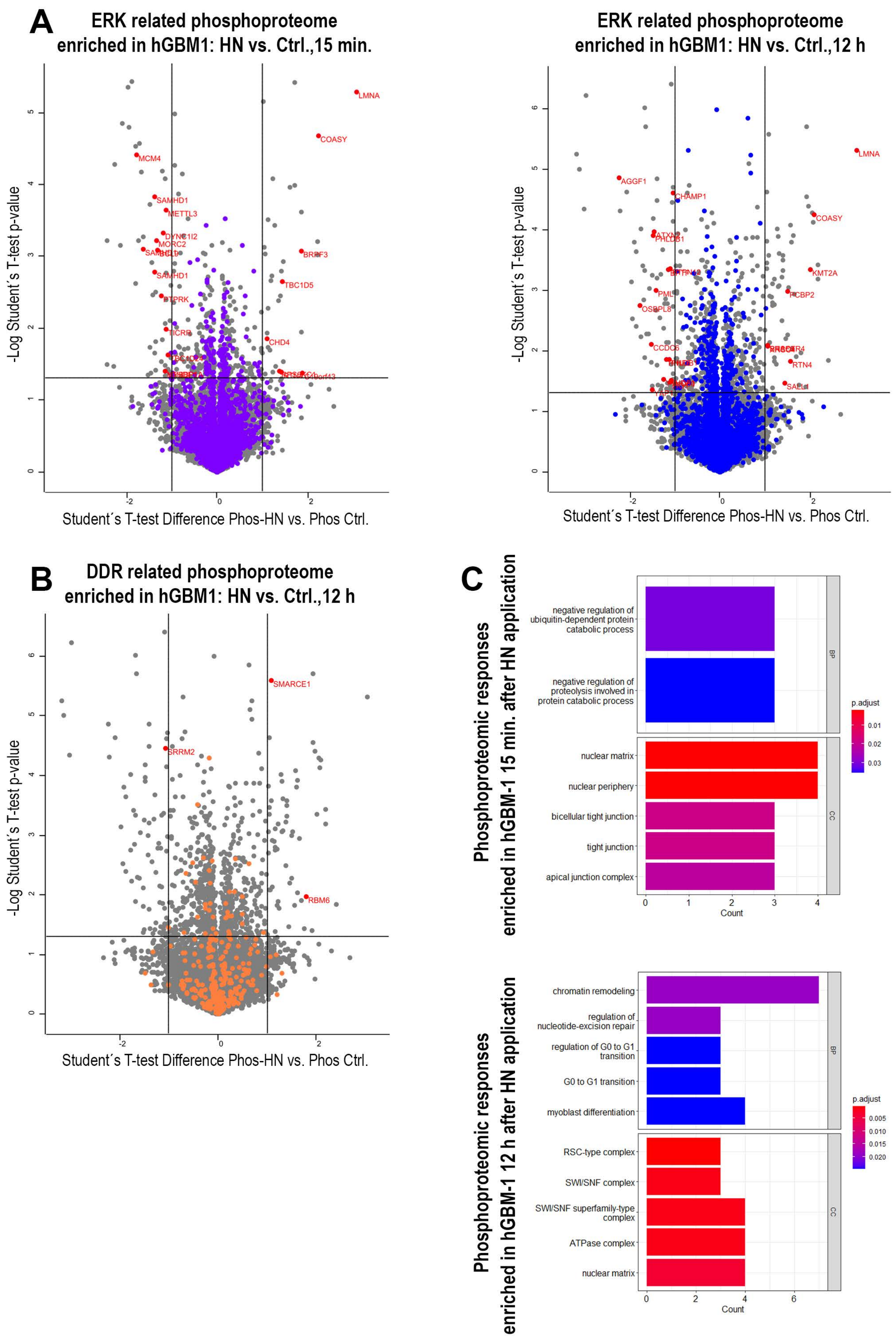

A
ERK related phosphoproteome
enriched in hGBM1: HN vs. Ctrl.,15 min.
-Log Student´s T-test p-value
Student´s T-test Difference Phos-HN vs. Phos Ctrl.
LMNA
COASY
MCM4
SAMHD1
METTL3
BRPF3
SAMHD1
TBC1D5
PTPRK
CHD4
ERK related phosphoproteome
enriched in hGBM1: HN vs. Ctrl.,12 h
-Log Student´s T-test p-value
Student´s T-test Difference Phos-HN vs. Phos Ctrl.
LMNA
AGGF1
CHAMP1
COASY
PHLDB1
KMT2A
PCBP2
PML
OSBPL8
RTN4
SALL1
B
DDR related phosphoproteome
enriched in hGBM1: HN vs. Ctrl.,12 h
-Log Student´s T-test p-value
Student´s T-test Difference Phos-HN vs. Phos Ctrl.
SMARCE1
SRRM2
RBM6
C
Phosphoproteomic responses
enriched in hGBM-1 15 min. after HN application
negative regulation of ubiquitin-dependent protein catabolic process
negative regulation of proteolysis involved in protein catabolic process
nuclear matrix
nuclear periphery
bicellular tight junction
tight junction
apical junction complex
BP
CC
Count
p.adjust
0.01
0.02
0.03
Phosphoproteomic responses
enriched in hGBM-1 12 h after HN application
chromatin remodeling
regulation of nucleotide-excision repair
regulation of G0 to G1 transition
G0 to G1 transition
myoblast differentiation
RSC-type complex
SWI/SNF complex
SWI/SNF superfamily-type complex
ATPase complex
nuclear matrix
BP
CC
Count
p.adjust
0.005
0.010
0.015
0.020

Figure S16: Phosphoproteome analysis confirms activation of ERK and DDR pathways in GBM cells downstream of Humanin-GP130 signaling. Related to Figure 5. To determine the signaling cues downstream of Humanin-induced GP130 activation, we investigated global protein phosphorylation by mass spectrometry. (**A, B**) Volcano plot with phosphopeptides quantified by label-free phosphoproteome analysis. Phosphopeptides are plotted according to p-value and difference caused by treatment of hGBM-1 cells with HN (200 nM) for 15 min or 12 hours in comparison to untreated cells (n=3 independent experiments). Phosphopeptides that change significantly in abundance (ttest p-value < 0,05; |ttest Difference|> 1) and contain phosphosites (blue) matching ERK (A-B) or ATM/ATR consensus motifs for DNA damage response (DDR) are shown as red dots; significantly changed phosphopeptides without consensus motifs are shown in black. (**C**) Phosphoproteomic responses enriched in hGBM-1 cells were obtained by Gene Ontology (GO) enrichment analysis showing involved biological processes (BP) and cellular components (CC). HN treatment protects hGBM-1 cells from proteolysis and ubiquitin-dependent degradation at short incubation time (15 min) while it is involved in chromatin remodeling and cell cycle control.

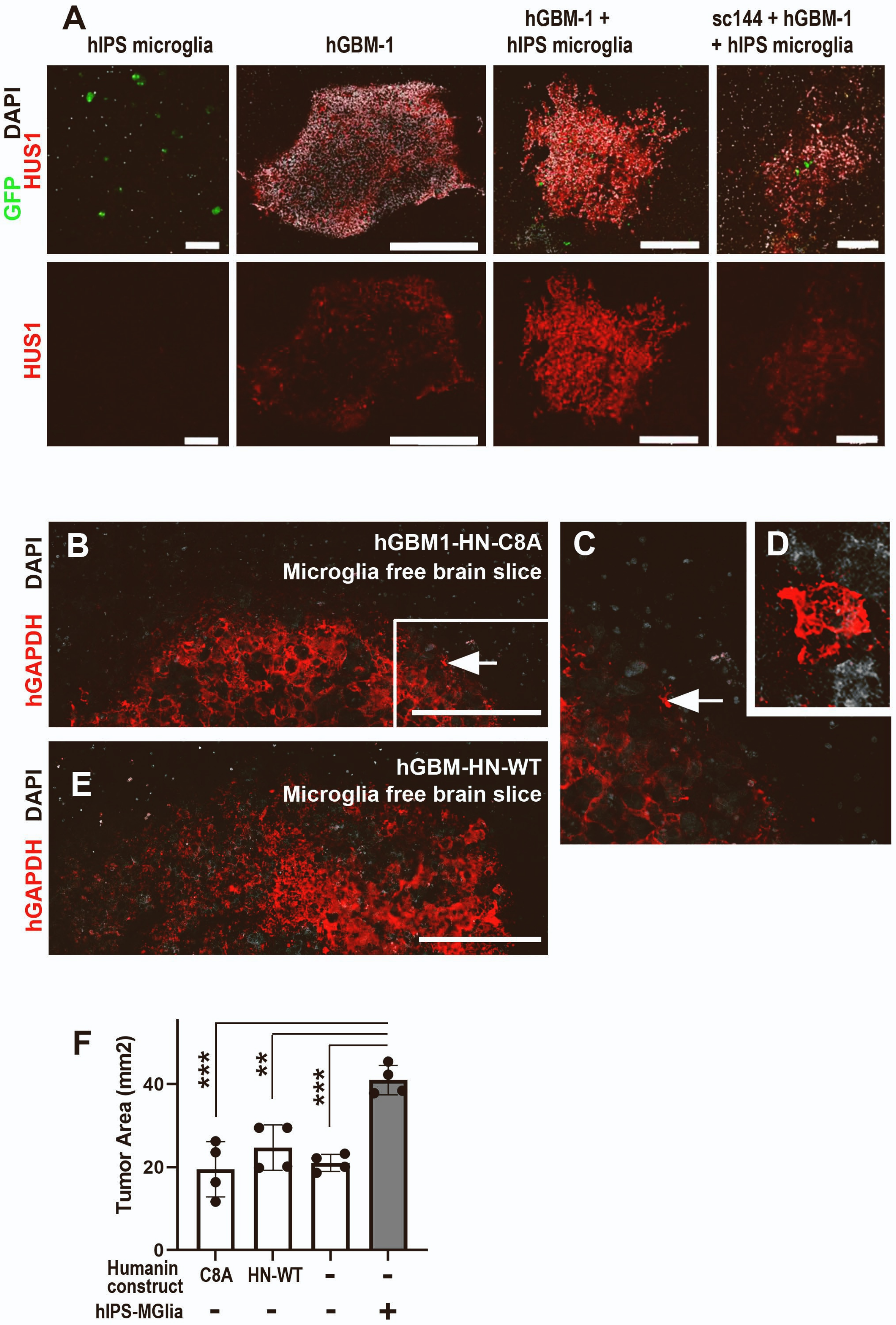

A
hIPS microglia
hGBM-1
hGBM-1 + hIPS microglia
sc144 + hGBM-1 + hIPS microglia
GFP HUS1 DAPI
HUS1
B
hGBM1-HN-C8A
Microglia free brain slice
DAPI
hGAPDH
C
D
E
hGBM-HN-WT
Microglia free brain slice
F
Tumor Area (mm2)
0
20
40
***
**
***
Humanin construct
C8A
HN-WT
-
-
hIPS-MGlia
-
-
-
+

Figure S17: Interaction of GBM with GAM induces Hus1 expression in a GP130 dependent manner. Related to Figures 5 and 6. (A) Confocal scanning in organ cultures under different conditions (see top row in figure) shows immunofluorescence for HUS1 (red); note that hIPS-microglia express GFP. HUS1 is undetectable in slices containing hIPS-microglia alone, detectable at lower level in slices containing hGBM alone and most prominent when hIPS-microglia are co-inoculated with hGBM; in slices containing both hGBM and hIPS-microglia HUS1 expression is blunted by sc144. (**B-F**) hGBM1-HN-C8A or hGBM-HN-WT cells were implanted in microglia depleted brain slices and analyzed by immunofluorescence against human-specific GAPDH (hGAPDH in red). (**B**) In hGBM1-HN-C8A brain slice cultures single invasive cells were scarce (see inset marked by arrow and magnifications of it in **C** and **D**) and this did not change with hGBM-HN-WT tumors (**E**). (**F**) Invasive hGAPDH-positive GBM cells and the main tumor mass was quantified in brain slices with or without coculture of hIPS-microglia (GFP+ cells were excluded when measuring invasiveness). No difference was observed when measuring the overall invasive tumor area upon implantation of hGBM1-HN-C8A, hGBM-HN-WT or parental hGBM-1 cells in microglia-depleted slices. However, when co-implanted with hIPS-microglia the tumor area of hGBM-1 cells was significantly increased (n = 4 organotypic brain slices per experimental group). Scale bars in (**A**) hIPS-microglia, hGBM-1 and hGBM1+ hIPS-microglia samples represent 500 µm, scale bars in hGBM-1+hIPS-MG+SC144 are 250 µm; scales are 500 µm in (**B** and **E**), 10 µm in (**D**). Statistical significance in (**F**) was assessed by One-Way-ANOVA: $^{**}p < 0.01$, $^{***}p < 0.001$.

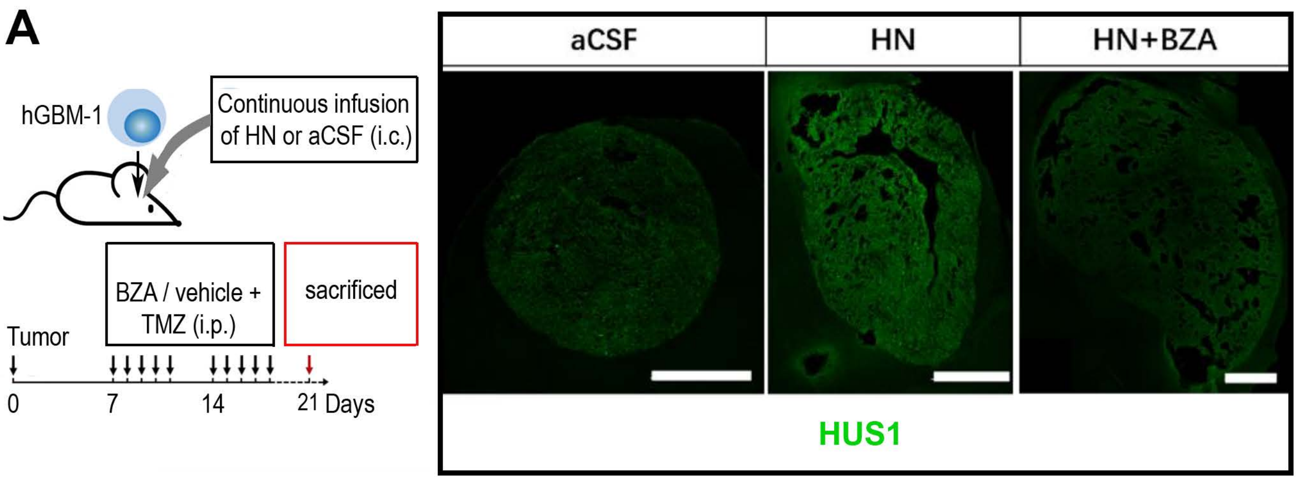
A
hGBM-1
Continuous infusion of HN or aCSF (i.c.)
BZA / vehicle + TMZ (i.p.)
sacrificed
Tumor
0
7
14
21 Days
aCSF
HN
HN+BZA
HUS1

Figure S18: Humanin-GP130 signaling induces HUS1 expression in GBM. Related to Figure 6. Orthotopic GBM were continuously (throughout the entire experimental time course) infused with Humanin or artificial cerebrospinal fluid (aCSF, as a control); 7 days after GBM inoculation (when tumor take was verified) mice were i.p. injected with TMZ and co-treated either with BZA or vehicle (as summarized in the schematic); mouse brains were immunofluorescently labeled for HUS1 after 21 days; note that intratumoral infusion of Humanin but not aCSF infusion promoted HUS1 expression and that the Humanin induced effect was blunted by cotreatment with BZA. Scale bars are 1 mm.

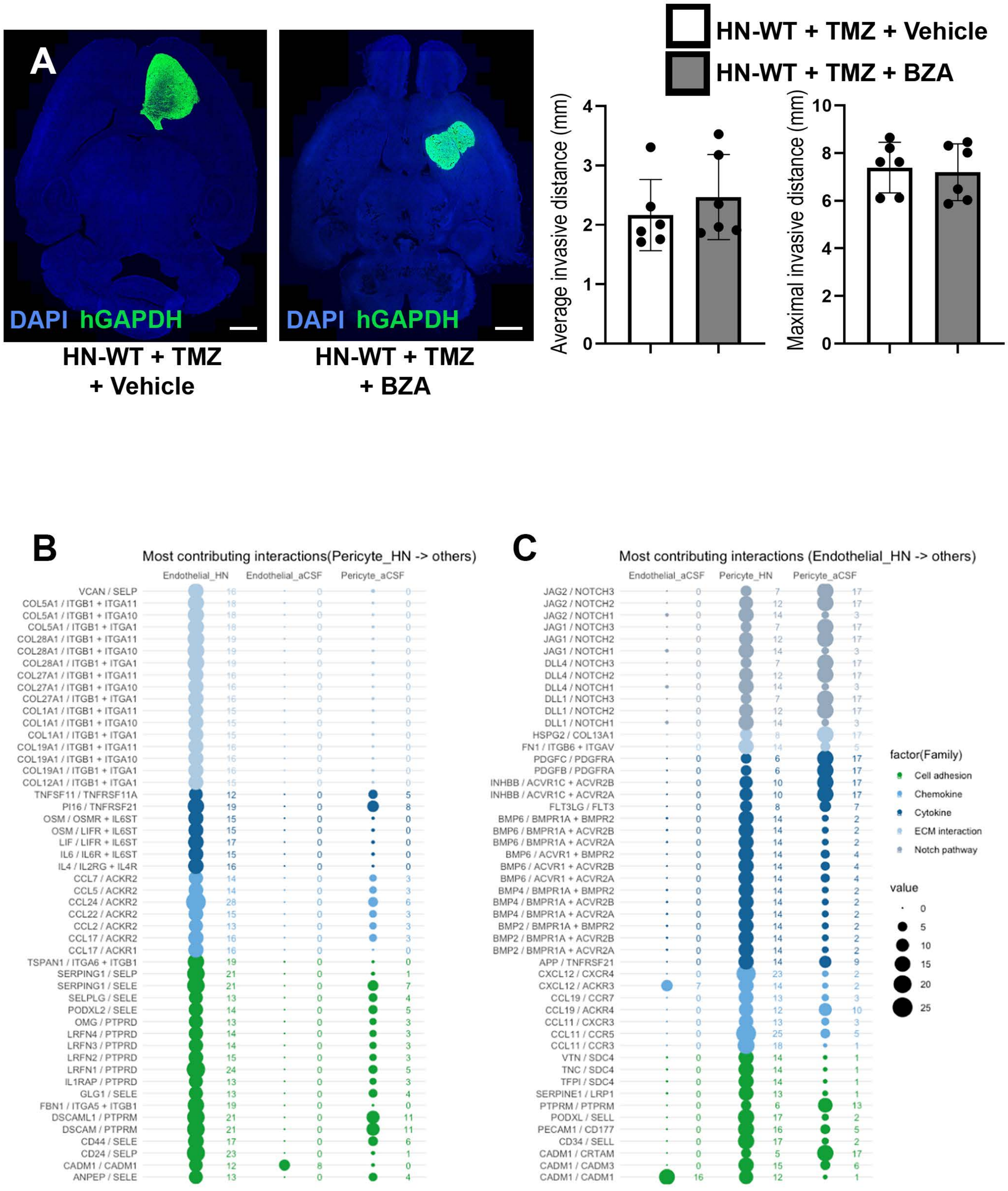
A
DAPI hGAPDH
HN-WT + TMZ + Vehicle
HN-WT + TMZ + BZA
Average invasive distance (mm)
Maximal invasive distance (mm)
B
Most contributing interactions(Pericyte_HN -> others)
Endothelial_HN
Endothelial_aCSF
Pericyte_aCSF
C
Most contributing interactions (Endothelial_HN -> others)
Endothelial_aCSF
Pericyte_HN
Pericyte_aCSF
factor(Family)
Cell adhesion
Chemokine
Cytokine
ECM interaction
Notch pathway
value

Figure S19: HN-induced GP130 signaling does not affect in vivo GBM cell invasion but alters crosstalk of pericyte with endothelial cells. Related to Figure 7. Mice with established, orthotopic HN-WT tumors received TMZ (50 mg/kg) and were either co-treated with BZA or vehicle; after three weeks of tumor growth samples were collected. (**A**) Brain sections were immunostained with an antibody specifically detecting human hGAPDH (highlighting xenografted human GBM). Immunofluorescence pictures of horizontal brain sections are shown with DAPI-positive nuclei (blue). Brain sections were inspected for single invasive tumor cells and the average and the maximal invasive distance was quantified. No significant difference was obtained by students ttest between the experimental groups (n = 6 animals per group). (**B, C**) Mouse GBM were intracerebrally treated with Humanin peptide or vehicle (aCSF). To investigate Humanin-dependent cell-cell communication in the brain tumor vasculature, the cell type specific transcriptome was obtained after FACS sorting of tumor-derived pericytes or endothelial cells. Cell-cell communication of purified cells was analyzed from transcriptomic data using ICELLNET and the murine CellPhoneDB as a reference (data are from n=4 mice for Pericyte_HN and n=3 mice for Percyte_aCSF; n=2 mice for Endothelial_HN or Endothelial_aCSF). Interestingly, upon Humanin treatment pericyte communicate by upregulating several ligands for the GP130 receptor on endothelial cells. Also, many extracellular matrix and cell adhesion interactions between the two cell types get induced. (**B**) HN-treated pericytes (Pericyte_HN) show most interactions with HN-treated endothelial cells (Endothelial_HN), while change of interactions with themselves (Percyte_aCSF) or with endothelial cells (Endothelial_aCSF) without HN-treatment are minimal. (**C**) HN-treated endothelial cells communicate with HN-treated pericytes through BMP, NOTCH and PDGFR -pathways. Plotted bubble bubble sizes indicate communication scores, while bubble color represents the classification (family) of different ligand-receptor pairs. Dotplots displaying the top 50 statistically significant interactions are shown. Scale bars in (A) represent 1.3 mm.

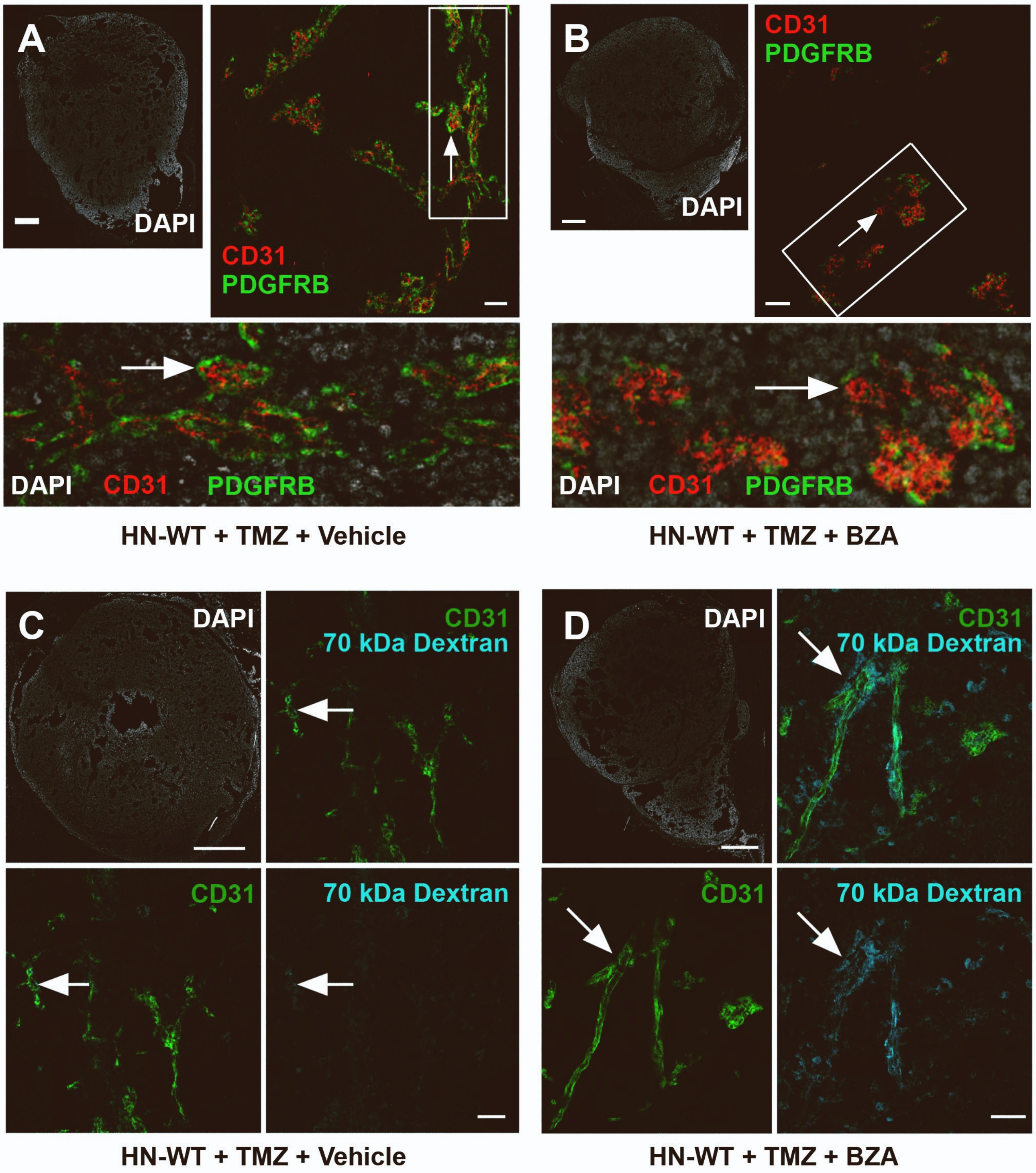
A
DAPI
CD31
PDGFRB
DAPI CD31 PDGFRB
HN-WT + TMZ + Vehicle
B
DAPI
CD31
PDGFRB
DAPI CD31 PDGFRB
HN-WT + TMZ + BZA
C
DAPI
CD31
70 kDa Dextran
CD31
70 kDa Dextran
HN-WT + TMZ + Vehicle
D
DAPI
CD31
70 kDa Dextran
CD31
70 kDa Dextran
HN-WT + TMZ + BZA

Figure S20: HN-induced GP130 signaling in GBM cell affects pericyte coverage and blood-tumor barrier formation. Related to Figure 7. (**A, B**) The tumor vasculature of orthotopically implanted human GBM cells was investigated by immunofluorescence staining against vascular endothelial (CD31) and mural (PDGRB) cells. Blockage of GP130 by BZA in mice (implanted with HN-WT cells and TMZ treated) reduced the coverage (arrows) of endothelia cells (CD31) by pericytes (PDGFRB), this was not observed in vehicle treated controls. **(D-E)** Intravenous administration of 70kDa Dextran (blue) in advanced GBM was used to assess the blood tumor barrier. In BZA-treated tumors fluorescently-labelled 70kDa dextran (blue) can be observed in the tumor parenchyma next to the CD31+vessel area (arrows), this was much less apparent in vehicle-treated mice. Scale bars in the overviews (in A, B) are 500 µm; and 20 µm in all other micrographs.
