# Supplementary material for: Myeloid cells coordinately induce glioma cell-intrinsic and cell-extrinsic pathways for chemoresistance via GP130 signaling
Source: Cell Rep Med. 2024 Jul 24;5(8):101658. doi: 10.1016/j.xcrm.2024.101658 (PMC11384956; doi:10.1016/j.xcrm.2024.101658)
Supplement: Document S2. Article plus supplemental information [file mmc13.pdf]

Cell Reports
Medicine

Article

Myeloid cells coordinately induce glioma cell-intrinsic and cell-extrinsic pathways for chemoresistance via GP130 signaling

Graphical abstract

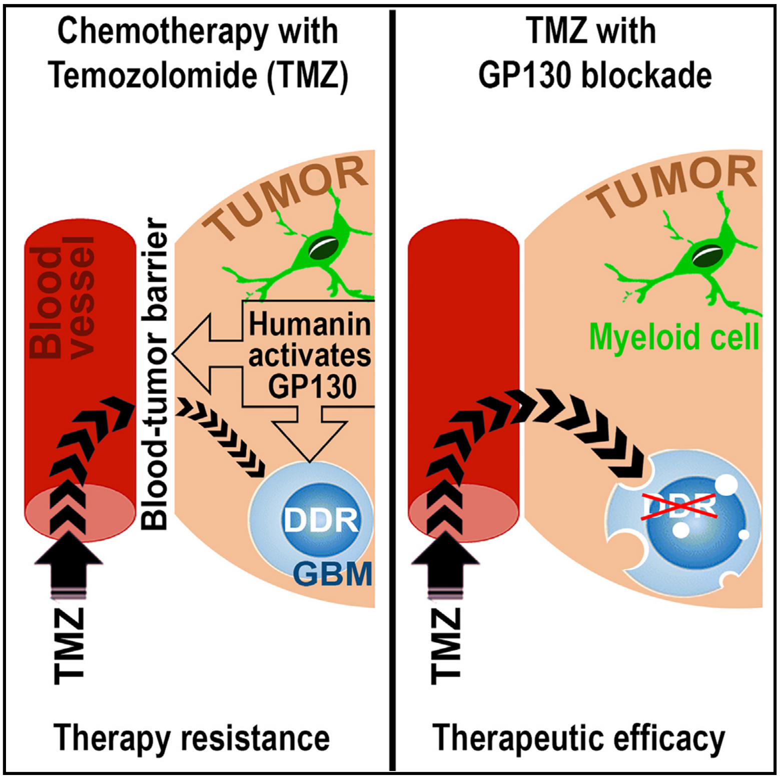

Highlights

- Interaction of myeloid and tumor (GBM) cells induces humanin expression
- GBMs segregate inter-individually into humanin-sensitive or humanin-insensitive tumors
- In sensitive GBMs, humanin induces cell-autonomous and systemic chemoresistance
- Humanin-mediated therapy resistance can be addressed with clinically approved drugs

Authors

Jiying Cheng, Min Li, Edyta Motta, ..., Charlotte Flüh, Roland E. Kälin, Rainer Glass

Correspondence

rainer.glass@med.uni-muenchen.de

In brief

Cheng et al. show that glioblastoma (GBM) and myeloid cell interaction initiates paracrine signaling via the peptide humanin. Humanin activates GP130 receptors on vascular and GBM cells resulting in blood-tumor barrier formation and DNA damage response (DDR). This represents a bifurcated resistance pathway for chemotherapy. Therapeutic efficacy is restored by GP130 blockade.

Cheng et al., 2024, Cell Reports Medicine *5*, 101658
August 20, 2024 © 2024 The Author(s). Published by Elsevier Inc.
https://doi.org/10.1016/j.xcrm.2024.101658

CellPress

Cell Reports Medicine

CellPress
OPEN ACCESS

Article

Myeloid cells coordinately induce glioma cell-intrinsic and cell-extrinsic pathways for chemoresistance via GP130 signaling

Jiying Cheng,[1,2,13] Min Li,[1,13] Edyta Motta,[3] Deivi Barci,[1] Wangyang Song,[1] Ding Zhou,[1] Gen Li,[1] Sihan Zhu,[1] Anru Yang,[1] Brian D. Vaillant,[4] Axel Imhof,[5] Ignasi Forné,[5] Sabine Spiegl-Kreinecker,[6,7] Nu Zhang,[2] Hiroshi Katayama,[8] Krishna P.L. Bhat,[9] Charlotte Flüh,[10] Roland E. Kälin,[1,6,13] and Rainer Glass[1,11,12,13,14,*]

[1]Neurosurgical Research, University Hospital, LMU Munich, Munich, Germany
[2]Department of Neurosurgery, The First Affiliated Hospital of Sun Yat-sen University, Guangzhou, Guangdong, P.R. China
[3]Max Delbrück Center for Molecular Medicine in the Helmholtz Association, Berlin-Buch, Germany
[4]Department of Neurology, Dell Medical School, The University of Texas at Austin, Austin, TX, USA
[5]Protein Analysis Unit, Biomedical Center (BMC), Faculty of Medicine, Ludwig-Maximilians-University (LMU) Munich, Martinsried, Germany
[6]Department of Neurosurgery, Medical Faculty, Johannes Kepler University Linz, Linz, Austria
[7]Clinical Research Institute for Neurosciences, Johannes Kepler University Linz, Linz, Austria
[8]Department of Translational Molecular Pathology, The University of Texas MD Anderson Cancer Center, Houston, TX, USA
[9]Department of Cancer Biology, Mayo Clinic, Scottsdale, AZ, USA
[10]Department of Neurosurgery, University Medical Center Göttingen, Göttingen, Germany
[11]German Cancer Consortium (DKTK), partner site Munich, a partnership between DKFZ and University Hospital Munich, Munich, Germany
[12]Institute of Surgical Research at the Walter Brendel Centre of Experimental Medicine, University Hospital, LMU Munich, Munich, Germany
[13]These authors contributed equally
[14]Lead contact
*Correspondence: rainer.glass@med.uni-muenchen.de
https://doi.org/10.1016/j.xcrm.2024.101658

SUMMARY

The DNA damage response (DDR) and the blood-tumor barrier (BTB) restrict chemotherapeutic success for primary brain tumors like glioblastomas (GBMs). Coherently, GBMs almost invariably relapse with fatal outcomes. Here, we show that the interaction of GBM and myeloid cells simultaneously induces chemoresistance on the genetic and vascular levels by activating GP130 receptor signaling, which can be addressed therapeutically. We provide data from transcriptomic and immunohistochemical screens with human brain material and pharmacological experiments with a humanized organotypic GBM model, proteomics, transcriptomics, and cell-based assays and report that nanomolar concentrations of the signaling peptide humanin promote temozolomide (TMZ) resistance through DDR activation. GBM mouse models recapitulating intratumoral humanin release show accelerated BTB formation. GP130 blockade attenuates both DDR activity and BTB formation, resulting in improved preclinical chemotherapeutic efficacy. Altogether, we describe an overarching mechanism for TMZ resistance and outline a translatable strategy with predictive markers to improve chemotherapy for GBMs.

INTRODUCTION

Gliobastomas (GBMs) are the most frequent malignant brain tumors among adults.[1] Current clinical care for GBMs is largely palliative, and the poor response of GBMs to chemotherapy is a major barrier for successful therapy.[1,2] One key component promoting temozolomide (TMZ, the chemotherapeutic standard of care) resistance is the DNA damage response (DDR) pathway in tumor cells.[3] The repair of TMZ-induced DNA single-strand breaks is controlled by the ataxia telangiectasia and Rad3-related (ATR) kinase,[3] which is recruited to stalled or collapsed replication forks together with the 9-1-1 DNA clamp complex.[4,5] These molecules cooperatively enable a time window for DNA repair and promote recovery from a DNA replication arrest.[4,5]

Therapy resistance of GBMs is not only regulated through cell-intrinsic pathways but also evoked by the tumor microenvironment.[2,6] Myeloid cells (GAMs; comprising both bone-marrow-derived macrophages and microglia) and vascular cells are among the most abundant microenvironmental cell populations in GBMs and exert tumor-supporting effects.[6–8] The extensive vascular network in GBMs enables rapid expansion of the tumor mass.[2,9] At the same time, the GBM vasculature insufficiently supplies blood-borne therapeutics into GBMs, since intratumoral vessels maintain a residual,

This is an open access article under the CC BY license (http://creativecommons.org/licenses/by/4.0/).

locoregionally heterogeneous, barrier function.[9,10] This blood-tumor barrier (BTB) partly recapitulates mechanistic features of the blood-brain barrier, which is required for brain homeostasis.[9–11] However, despite large efforts to tackle the BTB, efficacious translational approaches to pharmacologically improve the transport of therapeutics across the blood vessel wall are scarce.[9]

GAMs are known to have protumorigenic functions by inducing distinct pathological traits in subsets of GBMs.[7,8] For example, a GBM-dependent role of GAMs to promote chemoresistance[12] or accelerate GBM vascularization was previously suggested.[13] However, it remained unclear whether GAMs have multiple, synergistic roles for neoplastic progression in a single tumor. Here, we show that GAMs exert an overarching, pathologically coordinating function in GBMs. Our transcriptomic and immunohistological data from human GBMs, humanin-expressing GBM mouse models, and humanized organotypic cultures indicate that paracrine signaling between GBMs and GAMs induces TMZ resistance in tumor cells and simultaneously promotes BTB formation. Hence, GAMs provide GBMs with a dual protection from chemotherapy: individual GBM cells gain improved ability for DNA repair, and intratumoral delivery of TMZ is attenuated. Remarkably, both mechanisms rely on a single signaling cue and can be synchronously inhibited by blunting GP130 receptor activity. Overall, we report a central, pathologically coordinating function of GAMs and a translatable strategy to augment the standard of care in GBMs.

RESULTS

Humanin is abundantly expressed in GBMs

We purified GAMs from GBM biopsies or microglia from tumor-free human brains (Table S1) by flow cytometry according to established protocols[14] (Figures S1A–S1D). In addition, one part of each sample was excised to determine GBM subtypes (Table S1). Gene ontology analysis of differentially expressed genes between GAMs and microglia from tumor-free brains revealed an enrichment of mitochondrial metabolic pathways in GAMs (Figure 1A). Irrespective of GBM subtypes, GAMs overexpressed the mitochondrial 16S rRNA (*MT-RNR2*; as compared to controls), which contains an open reading frame (ORF) encoding the peptide humanin (Figure 1B).[15] Immunofluorescence inspection[16] of GBM samples (Table S2) revealed that GAMs strongly expressed humanin (Figures 1C and 1D) and showed that humanin was much more abundant in GBMs than in tumor-free human brain specimens (Figure 1E). Humanin expression was not restricted to GAMs (Figure 1D) but was also present in brain tumor cells, as shown in isocitrate dehydrogenase (IDH^{MUT}) astrocytoma cells (Figure 1F). The specificity of the immunolabeling procedure was carefully controlled (Figure S2), and representative data corresponding to Figure 1C are presented (Figure S3). All in all, immunofluorescence of GBM specimens showed that *MT-RNR2* was translated into the humanin signaling peptide in a broad range of tumors. We consistently observed expression of humanin at higher levels in human gliomas than in tumor-free human brain biopsies.

Myeloid and GBM cell interaction induces humanin expression

Next, we inspected humanin expression levels in cultivated mouse brain slices that were depleted from endogenous microglia[17] (Figures S4A–S4D) and replenished with human induced pluripotent stem cell (hiPSC)-derived microglia (since humanin is a human-specific peptide[15]). Some slice preparations also contained human stem-like GBM cells alone[18–20] (hGBMs; Table S3) or hGBMs together with hiPSC microglia (schematic in Figure 2A). Notably, expression levels of humanin were augmented when hiPSC microglia and GBM cells were coexistent (Figures 2A and S4E). Next, we determined if forced expression of humanin can have a pathological impact. Furthermore, we considered that humanin can modulate cell viability through intracellular or extracellular pathways[15] and generated hGBM cells stably expressing wild-type humanin peptide (HN-WT; mediating intra- and extracellular effects) and hGBM cells expressing humanin mutants that cannot be secreted (HN-L9R; retaining intracellular biological activity[21]) or have no function at all (HN-C8A[21]; Figure 2B). Expansion of these genetically manipulated hGBMs was quantified *in vitro* (Figure 2B). At the experimental endpoint, HN-WT cells had grown to much higher cell numbers as compared to hGBMs expressing humanin mutants, while the intracellularly active HN-L9R promoted viability only very moderately (as compared to the inactive HN-C8A controls or wild-type hGBM cells). This pinpointed a strong tumor-promoting effect by secreted humanin, which was evaluated in an immunodepletion experiment (we abstained from experiments with MT-RNR2 knockdowns since this led to deteriorated cell viability; Figure S5). Here, we generated conditioned medium from HN-WT cells that was either immunodepleted with a humanin-specific antibody or left under control conditions (using non-immune IgG), and then hGBM cells were cultured in the resulting media (see schematic in Figure 2C). When quantifying hGBM cell numbers, we observed that the HN-WT-induced protumorigenic effect (Figure 2B) was fully abrogated in hGBM cells exposed to humanin-depleted media but was preserved in controls (Figure 2C). Altogether, this series of experiments indicated that GAM and GBM cell interaction promotes humanin expression and that humanin release supports GBM growth.

Humanin induces chemoresistance in a subset of GBMs

We next addressed the question of whether humanin may have pathological potential in a wider set of GBMs. In a pharmacological assay, we exposed different hGBMs (Table S3) to different concentrations of synthetic humanin peptide (or the pharmacologically more potent mutant humanin-G [HNG][15]; in cell culture media free of standard growth factors, epidermal growth factor [EGF] and basic fibroblast growth factor [bFGF]). We observed that humanin-sensitive GBMs were growth stimulated specifically by nanomolar amounts of humanin (but not larger amounts), whereas insensitive GBMs required micromolar humanin or HNG concentrations (Figures S6A and S6B). The tumor-supportive effect was induced after repetitive humanin or HNG application but returned to control levels after humanin/HNG withdrawal (Figures S6C–S6F). Next, we investigated the pharmacological effects of humanin in more detail by exogenous application of humanin or HNG, which was partly combined with

CellPress
OPEN ACCESS

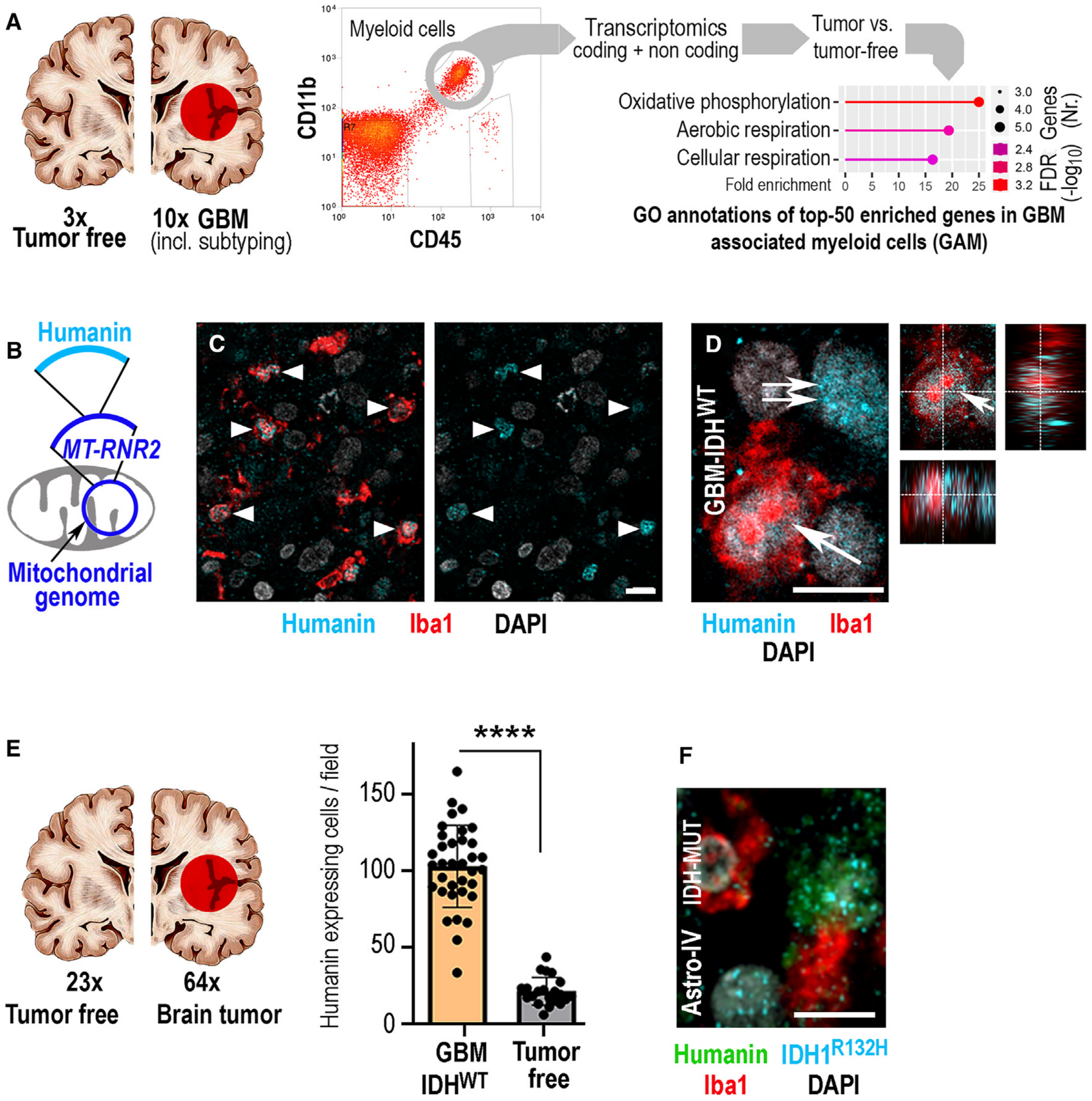

Figure 1. Humanin is strongly expressed in hGBMs

(A) Myeloid cells purified from biopsies of epilepsy surgery (tumor free) or GBMs underwent transcriptomic profiling and bioinformatics analysis.

(B) The mitochondrial ribosomal RNA-encoding gene MT-RNR2 is among the top-5 upregulated genes in GAMs. MT-RNR2 contains an open reading frame for the peptide humanin.

(C) Confocal microscopy of GBMs immunolabeled for humanin and the myeloid cell marker Iba1; GAMs expressing humanin are indicated (arrowheads).

(D) A single optical section of GAMs (arrow) and other intratumoral cells (double arrow) plus confocal cross hair inspection (insert).

(E) Immunofluorescence labeling for humanin in GBMs and controls was quantified (dots indicating individual patient samples).

(F) In IDH1-mutant (IDH1^{R132H}), grade-IV astrocytomas, humanin expression is largely confined to GBM cells. The number of biological replicates is indicated (each dot in the graph indicates average data from one individual sample); error bars are presented as mean ± SDM. Statistical significance is shown as false discovery rate (FDR) in (A) and by t test (****$p < 0.0001$) in (E); scales indicate 30 μm (C), 10 μm (D, F).

TMZ administration. Strikingly, we observed that humanin (with almost equal efficacy as HNG) rescued humanin-sensitive GBMs from the antitumorigenic effects of TMZ (Figures 2D–2F). Overall, we identified five GBM cultures undergoing chemoresistance in response to nanomolar humanin concentrations (here for brevity designated hGBM-1 to hGBM-5; Figure S7) and three GBM cultures requiring micromolar amounts of humanin for the induction of chemoresistance (hGBM-6 to hGBM-8; Figure S7). Flow cytometric analysis showed that TMZ strongly induced DNA fragmentation in hGBMs, which was blunted by coadministration of HN (20 nM) together with TMZ (100 μM; Figures 2G and 2H). Coherently, TMZ induced γ-H2AX foci[22] and caspase-3 activity[22] in GBM cells (Figure S8). Simultaneously, TMZ reduced labeling for the cell cycle marker Ki67[22] (Figure S8). Humanin application reverted these chemotherapeutic effects in GBM cultures (Figure S8). In summary,

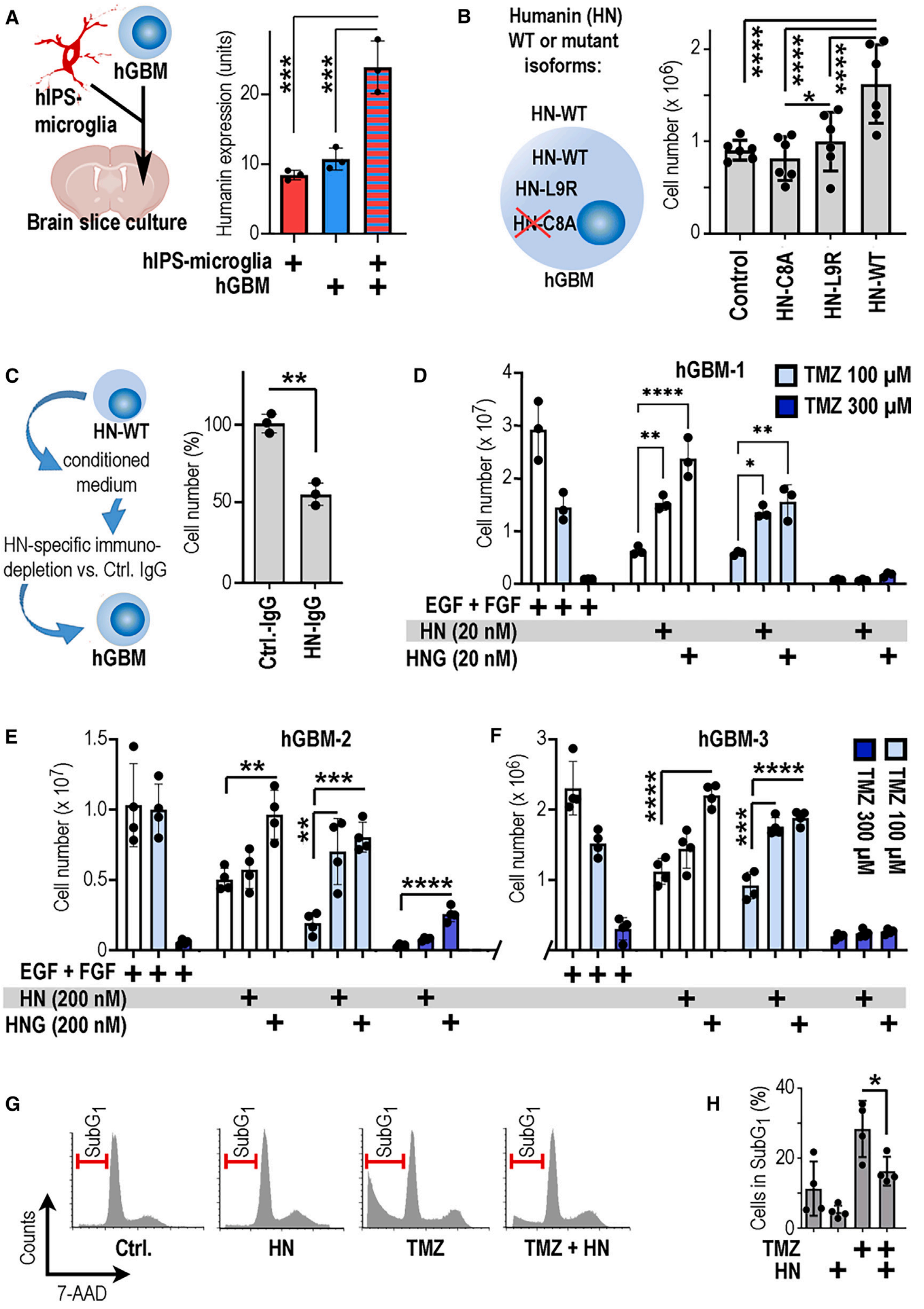

Figure 2. Humanin promotes GBM chemoresistance

(A) hiPSC microglia or hGBMs were implanted (alone or in combination) into organ-cultured mouse brain slices; immunostained for humanin and staining was quantified.

(legend continued on next page)

GBMs segregate into tumors with high or low humanin sensitivity. The interplay of GAMs and GBMs promotes increased humanin expression, which can lead to increased TMZ resistance specifically in humanin-sensitive GBMs.

The protumorigenic effect of humanin requires GP130 and ERK activation

Our experiments showed that tumor-supportive effects were specifically mediated by extracellular humanin. The multimeric interleukin receptors (containing the glycoprotein GP130)[15,23] and the formyl peptide receptors[15] have previously been identified as plasma membrane receptors for humanin. Hence, we quantified the mRNA expression levels of *IL6ST* (GP130) in both humanin-sensitive and humanin-insensitive hGBMs by qPCR (Figures 3A and S9A–S9E). We detected robust *IL6ST* levels in humanin-sensitive hGBM-1, 2, and 3, but not in humanin-insensitive GBMs. Formyl peptide receptors were undetectable (Figures S9C–S9E) in agreement with results from GBM databases (Figure S9F). Next, we quantified tumor cell expansion of hGBM-1, 2, and 3 stimulated with humanin or HNG (using the most efficacious growth-stimulating peptide variant for each hGBM subset; see Figures 2D–2F) in the presence or absence of the GP130 antagonist sc144.[24] We consistently observed that sc144 fully blocked humanin (or HNG)-stimulated hGBM growth (Figure 3B). In addition, we applied sc144 to hGBMs expressing HN-WT, HN-L9R, or HN-C8A. As expected, HN-WT tumor cells (without sc144) out-proliferated all other experimental groups, but application of sc144 fully blocked the growth-promoting effect of secreted humanin (Figure 3C); notably, HN-C8A or HN-L9R cultures remained unaffected by the antagonist (showing that sc144 has no toxic off-target effects). Of note, confirmatory results were obtained with an additional GP130 antagonist named bazedoxifene acetate (BZA; Figure S9G).[25] Altogether, this demonstrated that humanin required GP130 to stimulate the growth of GBMs.

We also investigated the impact of GP130 signaling on GAM and GBM crosstalk *in situ* by applying our organotypic brain slice model (as introduced in Figure 2A). Coinjection of hiPSC microglia with hGBMs induced strong humanin expression specifically in the brain tumor mass, which was significantly reduced by the coadministration of sc144 (Figure 3D; see also Figure S4E). We explored if humanin expression in GBM cells was controlled via established GP130 agonists[26] like ciliary neurotrophic factor, interleukin-6, leukemia inhibitory factor, oncostatin-M, or by humanin. Each cytokine was applied at an established bioactive concentration[27] and humanin was applied at 200 nM; after 16 h, cells were analyzed for humanin expression and cell morphology (Figures S10 and S11). As compared to unstimulated controls (Figure S10A), humanin induced profound morphological changes in GBM cells (by inducing the formation of cell protrusions; Figure S10B) and upregulated the expression levels for humanin, which was not observed with any of the other cytokines (Figures S10C and S11). Conditioned medium from hiPSC microglia cell cultures also induced protrusion formation and humanin expression in GBM cells, which was both blocked by GP130 inhibition (Figures S11D and S11E). Stimulation of hiPSC microglia with recombinant humanin peptide or with conditioned medium from GBM cell cultures also induced protrusion formation (depended on GP130), but did not lead to augmented humanin expression (Figures S12A and S12B). However, when we cocultivated hiPSC microglia with GBM cells, we consistently observed (GP130 dependent) induction of humanin expression in GBMs (Figure S4E) and also in hiPSC microglia (Figure S12C). In summary, this shows that the strong intratumoral humanin expression observed in many GBMs depends on GAM and GBM crosstalk via GP130 (Figure 3E). In brain tumor cells, humanin induces both biological reactions (chemoresistance and morphological alterations) as well as accelerated humanin expression. In microglia, the situation appears to be more complex; here humanin also induces morphological alterations, but humanin expression may also require physical contact with GBM cells.

Previous reports indicated that GP130 mediated therapy resistance through Stat3 activation.[15,28–30] Surprisingly, western blotting experiments of hGBMs exposed to HNG (versus vehicle controls) showed induction of mitogen-activated protein kinase (ERK1/2) signaling, whereas STAT3 or AKT activity was not altered (Figure 4A). Performing this experiment on a different timescale gave similar results (Figure S13). Therefore, we explored if ERK signaling may drive humanin-induced chemoresistance and applied the ERK-selective antagonist ravoxertinib (GDC-0994[31]) to hGBM-1, 2, and 3 cells treated with TMZ and humanin. We found that ravoxertinib fully and consistently abrogated humanin-induced chemoresistance (Figure 4B). To gain additional insight into the humanin-controlled signaling cues promoting TMZ resistance, we compared the transcriptional profile of humanin-sensitive (hGBM-1, 2, and 3) and humanin-insensitive hGBMs (hGBM-6, 7, and 8; Table S4) and noted that hGBM-1, 2, and 3 had elevated expression levels of genes relating to TMZ resistance and GP130 signaling (Figure S14A).

(B) Humanin (HN) exerts intra- and extracellular action, which can be inspected by distinct isoforms. The graph displays numbers of hGBMs expressing HN-WT, HN-C8A, HN-L9R, or unmanipulated controls.

(C) Conditioned media from HN-WT GBMs were immunodepleted for HN (HN-IgG) or not (Ctrl.-IgG), control medium (Ctrl.-IgG) supported GBM cell expansion, but not HN-depleted medium.

(D–F) hGBM-1, 2, or 3 was maintained under standard or under growth factor deprived conditions and partially supplemented with HN or the potent analog HNG. Partially, samples were exposed to temozolomide (TMZ) and cell numbers were quantified; note that nanomolar concentrations of humanin promote GBM chemoresistance.

(G) hGBM1 cells were maintained in growth factor-free medium (Ctrl.), stimulated with 20 nM humanin (HN), 100 μM TMZ, or both HN and TMZ (HN + TMZ). Cell cycle profiles were obtained by flow cytometry; note that TMZ shifts hGBM1 cells into a sub-G1 fraction (indicative of apoptosis), which is rescued by HN (data from one representative experiment).

(H) Quantification of the sub-G1 fraction (from the assay described in G; $n = 4$). The number of biological replicates is indicated (dots in graphs indicate data from individual experiments); all error bars are presented as mean ± SDM. Statistical significance was assessed by one-way ANOVA: *$p < 0.05$, **$p < 0.01$, ***$p < 0.001$, ****$p < 0.0001$.

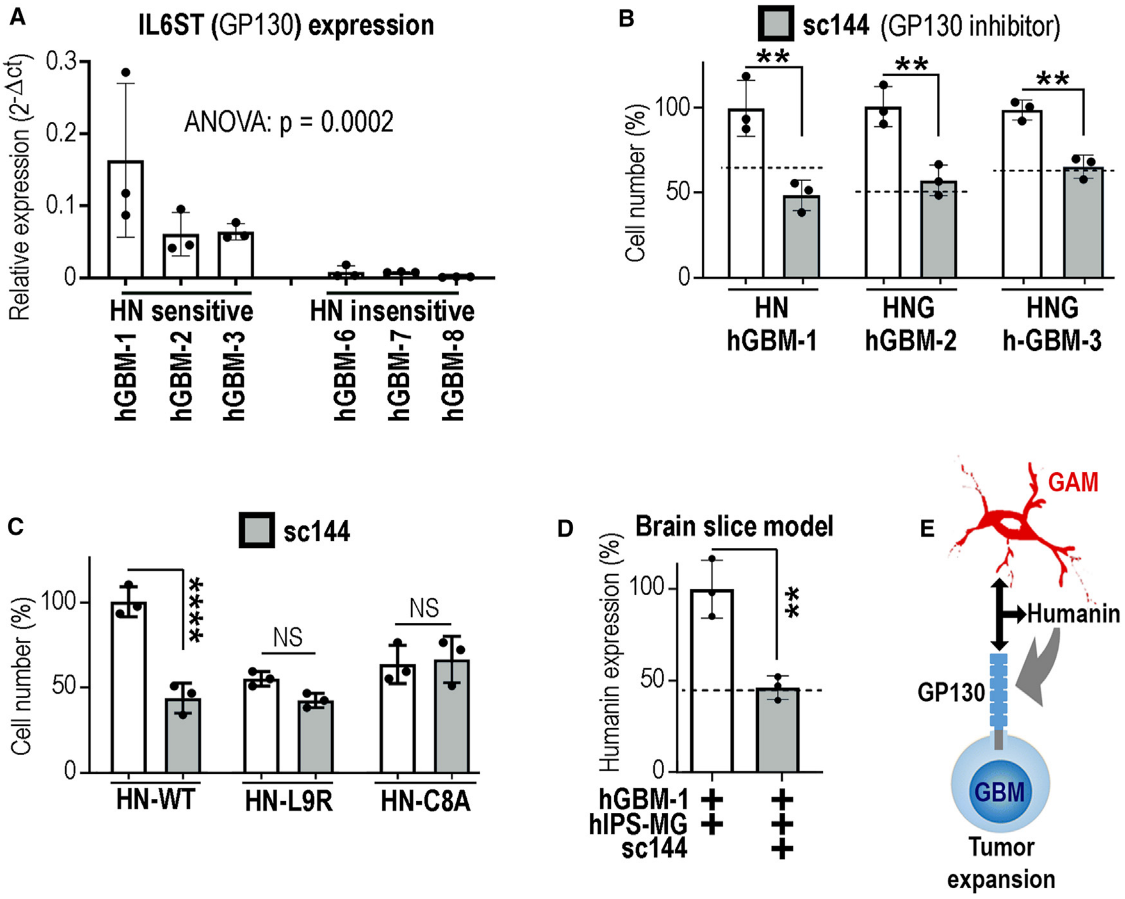

Figure 3. GP130 is essential for humanin-induced chemoresistance

(A) Quantitative reverse-transcription PCR (RT-PCR) for the humanin receptor subunit *IL6ST* (encoding GP130) was performed; note that *IL6ST* levels are much higher in humanin-sensitive than humanin-insensitive hGBMs.

(B) hGBMs were stimulated with HN or HNG, partly sc144 was coapplied, which consistently abrogated the protumorigenic effect of HN and HNG (dashed line: controls without HN or sc144).

(C) Expansion of hGBM1-HN-WT, HN-C8A, or HN-L9R cells, with or without sc144.

(D) Humanin expression levels in brain slices with hiPSC-derived microglia and hGBM1 cells were attenuated after addition of sc144 (graphically summarized in E). The number of biological replicates is indicated (dots in graphs indicate data from individual experiments); all error bars are presented as mean ± SDM. Statistical significance is shown by one-way ANOVA in (A) and t test in (B–D): $^{**}p < 0.01$; $^{****}p < 0.0001$; NS, not significant.

However, knockdown of a STAT3-activating component (Figure S14B and S14C) did not consistently blunt TMZ resistance in humanin-sensitive GBMs (Figure S14D). Altogether, our data support a central role for ERK (but not STAT3) signaling in humanin-induced chemoresistance.

We validated an immunohistochemical method detecting humanin-like peptides in mice[32] (Figure 4C) and detected a basal level of humanin immunopositivity in the cerebellum but not in the forebrain (Figure 4D). Orthotopic implantation of Gl261 cells (a mouse glioma cell line; Figures 4E and S2E) did not lead to humanin expression, but injection of hGBM1 (overexpressing HN-WT) into the brain resulted in strong and homogeneous humanin immunopositivity throughout the entire GBM area (Figures 4F and S2C). We used this model to treat established, orthotopic HN-WT GBMs with TMZ and coapplied (intraperitoneally [i.p.]) the MEK inhibitor mirdametinib (blocking MEK-induced ERK1/2 activation)[33] or vehicle (control). Mirdametinib, which is currently investigated in clinical trials for low-grade gliomas (NCT04923126), promoted survival throughout a chemotherapy schedule in our preclinical model (Figure 4G). Hence, our *in vitro* and *in vivo* experiments have consistently shown that ERK blockade is a therapeutically efficacious and fully translatable approach to blunt humanin-induced chemoresistance. Altogether, it broadens our view on the diverse pathological roles of the GP130 receptor subunit and indicates that stimulation of GP130 with humanin drives TMZ resistance through the ERK pathway (Figure 4H).

Humanin-induced chemoresistance is controlled by the HUS1, ATR pathway

To obtain further mechanistic insight how humanin promoted chemoresistance, we performed two consecutive RNA sequencing studies (Figures 5A and 5B) in humanin-sensitive GBMs. By filtering for the coherently expressed genes from

CellPress
OPEN ACCESS

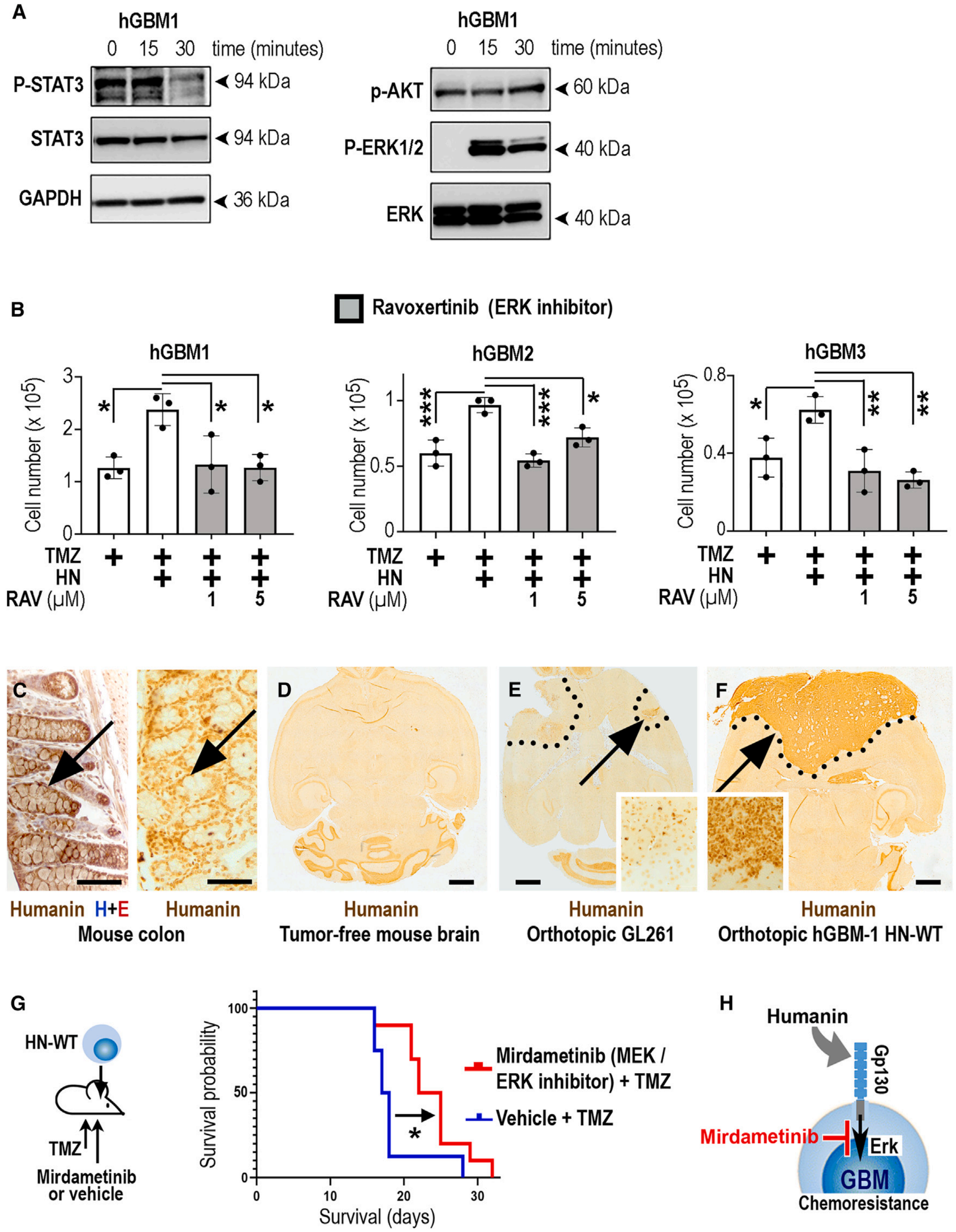

Figure 4. GP130-mediated ERK signaling is required for humanin-induced chemoresistance

(A) hGBM1 cells were stimulated with HNG or left untreated (0 min) and analyzed by western blotting.

(B) Application of HN improved the viability of TMZ-treated hGBM-1, 2, or 3. Cotreatment with the ERK inhibitor ravoxertinib (RAV) consistently abrogated HN-induced chemoresistance.

(C) Humanin-like peptide is expressed in the mouse colon (positive control; immunostaining partly counterstained with hematoxylin/eosin; H + E), but not in the mouse forebrain (D) or mouse gliomas (GL261; E).

(F) Humanin was immunolabeled in orthotopic HN-WT GBMs (tumor is indicated by a dotted line; area pointed out by arrow is magnified).

(legend continued on next page)

both experiments (Table S5), we uncovered a molecular network with functional annotations in the ATR-controlled DNA damage pathway, neurodegeneration, and apoptosis (Figures 5B and S15A). From the list of genes within this network, we explored which factors have clinical relevance in GBMs (Figure S15B). This showed a negative prognostic property for the humanin-induced gene *HUS1*, which was particularly pronounced for the mesenchymal GBM subtype (Figure 5C). Knockdown of *HUS1* in hGBMs strongly reduced cell viability (Figure S15C) in accordance with data from Cancer Dependency Maps (precluding further experiments using *Hus1* knockdown vectors; Figure S15D). In agreement with our observation on a central role of ERK signaling in humanin-mediated therapy resistance, we found that humanin-induced Hus1 expression was blunted with the ERK inhibitor ravoxertinib (Figures S15E and S15F). Coherent data were retrieved from a proteomics study comparing hGBM1 cells under control conditions or after stimulation with humanin (200 nM) for 15 min or 12 h (Figure S16). This showed that humanin did not trigger large changes in protein expression (Tables S6 and S7), but prominently altered the phosphoproteome (Tables S8 and S9). A pathway analysis of the phosphoproteome indicated that humanin modulated ERK signaling (Figure S16A) and the DDR (Figure S16B). The humanin-stimulated pathways included cell cycle regulation and DNA repair, and mechanistically, human-induced signaling trajectories correlate with chromatin remodeling (via the SWI/SNF complex; Figure S16C).

To investigate whether the humanin-stimulated HUS1 expression was induced by GAM/GBM interaction (and modulated by GP130), we used our organotypic brain slice model. Implantation of hiPSC microglia resulted only in a low HUS1 expression level within the slice preparation; hGBM cells (alone) had a higher HUS1 level, and coimplantation of hGBMs with hiPSC microglia accelerated HUS1 expression in a GP130-dependent manner (Figures 5D and S17A). HUS1 is an essential component of the RAD9A-RAD1-HUS1 (9-1-1) complex, which supports ATR-dependent DNA repair[5,34] and can thereby promote chemoresistance.[3] Since HUS1 knockdown resulted in GBM cell death (Figure S15C), and as 9-1-1 cannot be modulated pharmacologically, we blocked the DDR with the ATR inhibitor AZ20. We observed that AZ20 blunted the growth-promoting effect of HN-WT but not of mutant humanin controls (Figure 5E). Remarkably, ATR inhibition fully blocked the humanin-promoted chemoresistance in hGBMs-1, 2, and 3 (Figure 5F). The expression level of HUS1 and the extent of ATR activation (phosphorylation on threonine residue 1989; pT1989) were investigated by western blotting (Figure 5G; recapitulating the experimental schedules outlined earlier). We observed that HN-stimulated hGBM1 cells had increased levels of HUS1 and augmented activity of ATR (within 12 h, as compared to controls). Application of AZ20 (to HN-treated hGBMs) led to a profound reduction in HUS1 expression and ATR activity. Collectively, our data show that HUS1 and ATR are responsible for humanin-promoted GBM growth and chemoresistance (Figure 5H).

GP130 inhibition blocks humanin-induced chemoresistance *in vivo*

Further insight into humanin-induced therapy resistance was obtained with our *in vivo* model. Therefore, we orthotopically implanted HN-WT hGBM cells (secreting the active form of humanin) or HN-C8A cells (inactive humanin mutant) and applied TMZ (or vehicle) according to established schedules.[35] Mice were sacrificed at a fixed endpoint, and tumor size in each experimental group was quantified.[18] The HN-C8A tumors responded well to TMZ treatment, but HN-WT cells were fully rescued from the therapeutic effects of TMZ even after extended application (14 days) of a high TMZ concentration (Figure 6A). Both the *in vivo* model (Figure 6A) and the organotypic brain slice model (Figures S17B–S17F) showed that humanin does not promote tumor growth of GBMs that are embedded in a cellular microenvironment (in contrast to an *in vitro* situation; Figures 2D–2F). The main pathological effect of humanin *in vivo* is the induction of TMZ resistance (Figure 6A), which was also detected *in vitro* (Figures 2D–2F).

To study the *in vivo* pathways for this apparent chemoresistance in more detail, we injected hGBM-1 cells into mouse brains, infused humanin or artificial cerebrospinal fluid (aCSF) into the tumor mass, and treated the animals with i.p. injections of BZA, which is BTB permeable,[25] or vehicle (Figure 6B). Brains were harvested, and immunofluorescence for HUS1 (as a marker for humanin-induced GP130 signaling and chemoresistance) was inspected. Intratumoral infusion of aCSF generated tumors with weakly detectable levels of HUS1, while immunofluorescence intensity for HUS1 was much stronger in humanin-infused GBMs, which was blocked by i.p. application of BZA (Figure 6B; for higher magnification micrographs, see Figure S18). Since BZA blunted the humanin-stimulated expression of HUS1, we investigated if BZA would therapeutically support TMZ treatment in humanin-expressing GBMs. One week after implanting HN-WT cells into mouse brains (and verifying GBM growth), we treated all mice with TMZ and one cohort was i.p. cotreated with BZA, whereas another received vehicle (as a control; schematic in Figure 6C). At the predetermined endpoint, tumor size was assessed, and we noted that HN-WT GBMs receiving cotreatment with TMZ and BZA were substantially smaller than HN-WT tumors receiving TMZ plus vehicle (Figure 6C). Consequently, our series of *in vivo* studies showed that humanin promotes TMZ resistance of GBMs and that BZA restores the chemotherapeutic properties of TMZ (consistently, BZA promoted TMZ-induced tumor cell death; Figure 6D).

The detailed histopathological inspection of our humanin-expressing GBMs *in vivo* models (HN-WT GBMs) also suggested an angiogenic role of humanin. After immunolabeling these

(G) HN-WT GBMs (as in H) received TMZ together with systemic application (i.p.) of the MEK (ERK pathway) inhibitor mirdanetinib (10 mg/kg, i.p.; $n = 10$ mice) or vehicle ($n = 8$ mice) and overall survival was quantified.

(H) Schematic summary: extracellular humanin induces GP130-mediated chemoresistance in GBMs, which can be addressed therapeutically. The number of biological replicates is indicated (dots in graphs indicate data from individual experiments); all error bars are presented as mean ± SDM. Statistical significance is shown by one-way ANOVA in (B) or Mantel-Cox test (G): *$p < 0.05$, **$p < 0.01$, ***$p < 0.001$. Scales indicate 200 μm (C) or 1 mm (D –F).

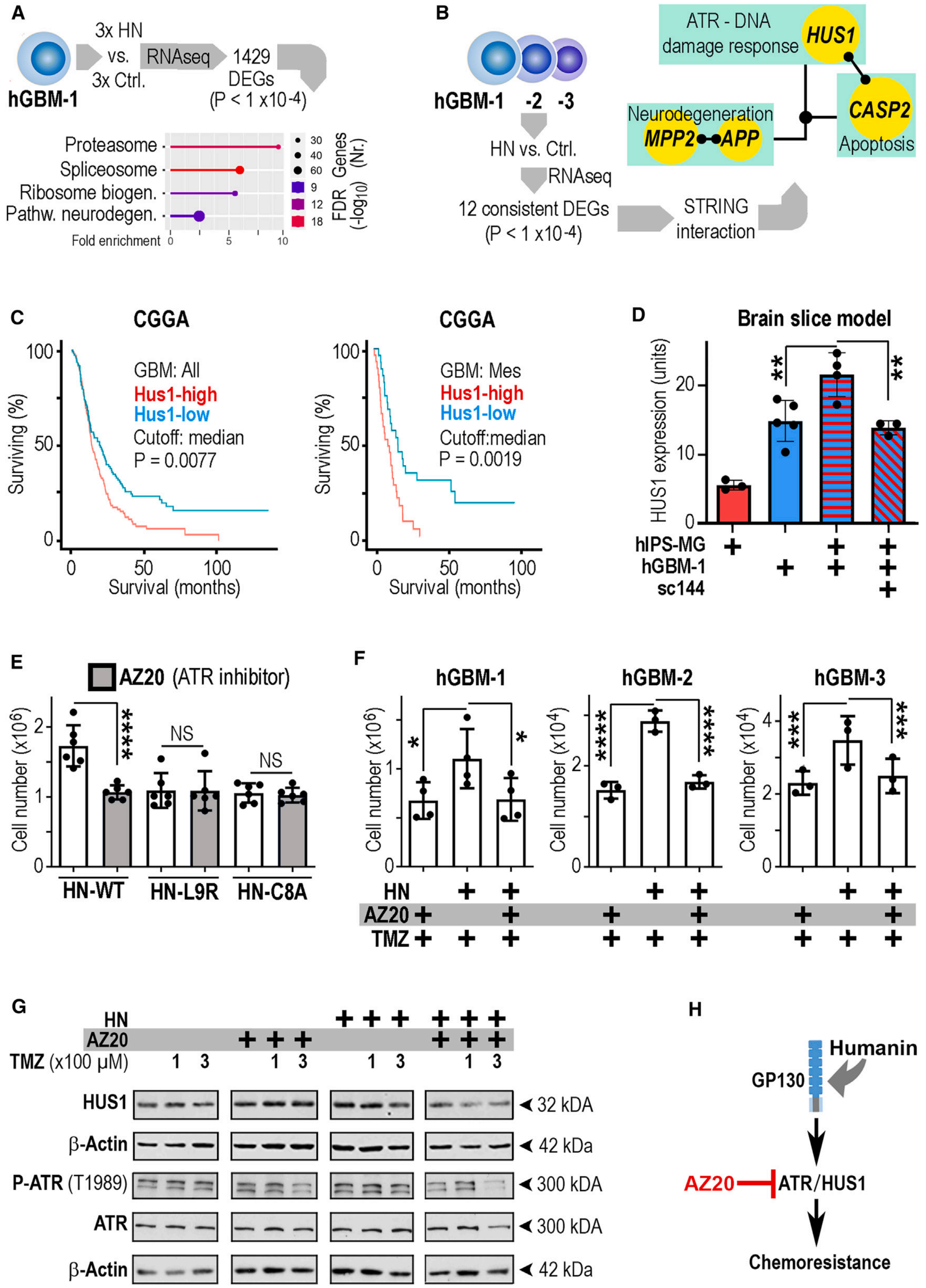

Figure 5. Humanin-induced chemoresistance requires ATR signaling

(A) hGBM1 cells were stimulated with HN or vehicle (Ctrl.), underwent transcriptomics, and differentially expressed genes (DEGs) were analyzed by bioinformatics.

(legend continued on next page)

preclinical tumor models with vascular markers, we found that HN-WT GBMs showed accelerated vascularization and altered vessel morphology (Figure 6E). In accordance with the anti-angiogenic role of TMZ,[36] chemotherapy blunted neoangiogenesis (Figure 6E). Strikingly, coapplication of BZA strongly supported the anti-angiogenic effects of TMZ application (Figure 6F) without accelerating GBM cell invasion (Figure S19A). Altogether, we observed a profound chemotherapy-supporting effect of BZA on both the level of GBM cytotoxicity and neoplastic angiogenesis.

GP130 inhibition blocks blood-tumor barrier formation

In HN-WT GBMs (versus HN-C8A controls), we noted a strong increase in platelet-derived growth factor receptor-beta (PDGFRB) expressing vascular mural cells (pericytes[37,38]), which was unaffected by TMZ application (Figure 7A). Furthermore, we observed that BZA-treated HN-WT GBMs did not exhibit increased pericyte coverage of tumor vessels (Figures 7B and 7C). Since the interaction of pericytes and endothelia may contribute to BTB formation and therapy resistance in GBMs,[37] we inspected both cell types in a transcriptomics experiment. We inoculated GL261 cells into transgenic mice allowing the tracing of pericytes (see STAR Methods), infused humanin or vehicle (aCSF), and purified (by flow cytometry) endothelia or pericytes from GBMs (Figure 7D). Analysis of differentially expressed genes (Table S10) and gene set enrichment analysis (GSEA) revealed a proangiogenic effect of humanin on endothelia (Figure 7D; differentially expressed genes for pericytes are listed in Table S11). Since interaction of vascular cells is required for barrier formation,[38] we inspected cell communication in humanin-infused versus control GBMs (with ICELLNET[39] and the murine CellPhoneDB). This revealed a profound impact of humanin on pericyte to endothelial communication (and vice versa; Figures S19B and S19C). We concentrated on the ligand-receptor pairs receiving the highest signaling scores in humanin (and not vehicle)-treated groups (Figure 7E), which revealed elevated bone morphogenetic protein (BMP) signaling (with established angiogenic traits[40]) from endothelia to pericytes. Notably, pericyte-derived factors were associated with IL6ST (GP130) receptor activation in endothelia, specifically in humanin-infused GBMs. A role for GP130 signaling endothelia-pericyte interaction was corroborated when quantifying the vascular mural cell coverage of GBM blood vessels in humanin-containing, TMZ-treated tumors (as compared to GBMs without BZA treatment; Figures 7F, S20A, and S20B). This indicated that systemic BZA application reduced vascular pericyte coverage in HN-WT GBMs and suggested that BZA treatment may interfere with BTB formation. We investigated this hypothesis by intravenous (i.v.) injection of a fluorescent vascular tracer (70 kDa) into the HN-WT GBM models in a chemotherapy paradigm with or without BZA application. Strikingly, we found that the tracer strongly and specifically accumulated in tumors of BZA-treated mice but not in control tumors (Figures 7G, S20C, and S20D). Altogether, this indicated that humanin promotes BTB formation, which is abrogated by BZA treatment. This finding opened the possibility that GP130 blockade in humanin-sensitive GBMs may exert a dual therapy-supporting role: GP130 inhibition promotes delivery of therapeutics by blunting the BTB and then suppresses chemoresistance of GBMs. To explore this scenario, we performed a survival study (using HN-WT GBMs plus TMZ treatments) and applied the specific GP130 inhibitor sc144 (or vehicle) via minipumps. Here, we did not apply BZA, as this substance (which is clinically used as an estrogen receptor antagonist[41]) had a side effect on body weight upon prolonged administration. We observed that sc144-mediated blockade of GP130 resulted in a strong TMZ therapy-supporting effect in humanin-secreting GBMs (Figure 7H). Hence, in humanin-sensitive GBMs, GP130 inhibitors have a strong, multi-modal chemotherapy-supporting effect (Figure 7I).

DISCUSSION

Resistance to TMZ is a major issue in clinical care for GBMs,[2] and cell-autonomous mechanisms preventing TMZ efficacy have predictive capacity in neurooncology.[1] Simultaneously, TMZ delivery into GBMs is locoregionally inefficient due to BTB formation.[9] However, diagnostic markers and therapeutic measures tackling BTB induction still require entry into routine clinical practice for neurooncology.[2,9] In the present study, we uncovered that GAMs promote chemoresistance both on the level of single tumor cells and on the level of systemic delivery of therapeutics. We identified high intratumoral levels of the peptide humanin as a risk factor for BTB development, showed that HUS1 is linked with humanin-mediated DNA repair, and uncovered GP130 inhibition as a treatment strategy to simultaneously suppress systemic and cell-autonomous resistance for TMZ.

MRI-based diagnostics for GBMs partly rely on an attenuated vascular barrier function in brain tumors.[1,9,10] However, with the advent of different imaging modalities in neurooncology, it became clear that vascular barrier formation is subject to profound locoregional heterogeneity[42] and that the BTB protects large parts of a given GBM from blood-borne therapeutics.[9] Our data exhibit an unforeseen role of humanin in controlling

(B) Experiments described in (A) were repeated with hGBM-1, 2, and 3 cells providing 12 consistent DEGs, of which several components assembled in a network.
(C) HUS1 was associated with outcome in human GBMs.
(D) In a myeloid-free brain sample, hGBMs have a basal level of HUS1 expression, which is upregulated by interaction with hiPSC microglia in a GP130-dependent manner.
(E and F) Contribution of the ATR pathway to humanin-induced GBM expansion (E) and chemoresistance (F) was demonstrated with the ATR inhibitor AZ20.
(G) Western blots showing expression levels of HUS1, ATR and beta-actin (loading control) and a readout for of ATR activation (pT1989) in hGBM1 cells treated with bovine serum albumin (control), TMZ, HN, or AZ20.
(H) In summary, AZ20 does not cooperate with TMZ per se, but blocks HN-induced TMZ resistance. The number of biological replicates is indicated (dots in graphs indicate data from individual experiments); all error bars are presented as mean ± SDM. Statistical significance is shown as FDR in (A), one-way ANOVA (D, E), or two-way ANOVA (F): *$p < 0.05$; ***$p < 0.001$; ****$p < 0.0001$; NS, not significant.

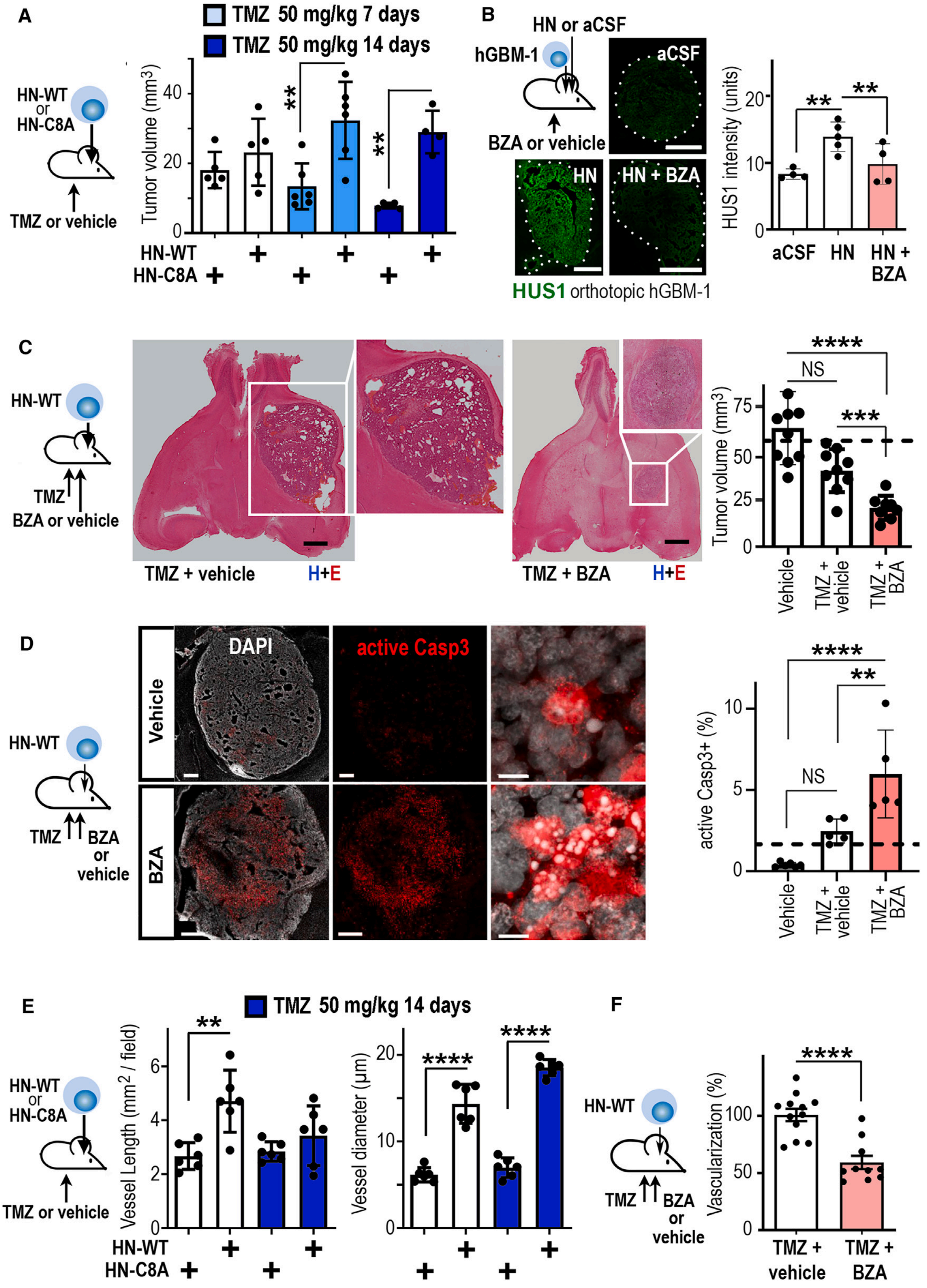

Figure 6. Humanin-induced chemoresistance can be blocked therapeutically

(A) Tumor size of orthotopic HN-WT or HN-C8a tumors was compared in mice receiving TMZ or vehicle (in animals with established tumor growth, 5x per week for 2 weeks; pre-defined endpoint was at 3 weeks).

(legend continued on next page)

brain tumor vascularization and BTB induction. We observed that humanin release in GBMs increases pericyte coverage in GBM vessels and blocks intratumoral accumulation of i.v. applied tracers. We demonstrate that humanin activates GP130 receptors in GBM cells and that GP130 inhibition blunts humanin-induced BTB induction. It is established that brain endothelia express GP130,[43] but only few pharmacological pathways for BTB modulation were previously discovered[44,45] and a role for GP130 in BTB maintenance was not explored.[25] Our data suggest GP130 inhibition in humanin-sensitive tumors as a promising option to promote TMZ entry into neoplastic areas and simultaneously block GBM DNA repair. This is supported by our *in vivo* studies showing that blunting cell-intrinsic mechanisms for TMZ resistance with mirdametinib had a moderate chemotherapy-supporting effect, while application of the GP130 antagonist sc144 leveraged TMZ-based therapy without apparent adverse effects mediated by sc144. In this context, it is important to note that the BTB-penetrating GP130 blocker BZA can have unwanted effects, since BZA is an estrogen receptor antagonist,[41] which reduced body weight in our preclinical models after prolonged application.

Currently, O6-methylguanine DNA methyltransferase (MGMT) methylation status serves as a stratifying marker for chemotherapy in GBMs.[1] However, the MGMT status alone does not always represent a fully satisfactory marker for treatment decisions.[46] The present study widens the repertoire of markers for TMZ application and shows that expression levels for humanin, HUS1, and GP130 are candidate predictive markers for TMZ treatment. We found that nanomolar concentrations of humanin are sufficient to induce GP130-mediated ER1/2 signaling and TMZ resistance via the ATR DDR pathway. Coherently, application of MEK/ERK inhibitors—and even more efficiently GP130 inhibitors—restored TMZ sensitivity. Hence, the development of BTB-penetrating GP130 antagonists is a promising route to augment TMZ treatment, and likely also more targeted therapies, in neurooncology.

The cytoprotective effects of humanin were discovered in neurodegenerative disease and have been confirmed in a larger number of studies.[15,21,23] Treatment of tumor cells with chemotherapeutics may contribute to humanin release.[47,48] However, this observation requires additional investigation since it was made with GBM cells corresponding to humanin-insensitive cells of the present study. We uncovered the pathological role of humanin after genetic screens in patient-derived GAMs, and our brain slice experiments showed that humanin is expressed upon interaction of GBM cells with GAMs. Subsequent *in vitro* assays indicated that secreted humanin initiates TMZ resistance at nanomolar concentrations of extracellular humanin.[49] This was confirmed by an immunodepletion experiment. Transcriptomics, western blotting, and genetic manipulation indicated a molecular mechanism for humanin-induced chemoresistance. Under *in vitro* conditions, humanin exerted even a wider spectrum of tumor-supporting traits and also promoted GBM cell expansion. This was not observed in our orthotopic mouse models. We interpret that humanin-induced chemoresistance is a robust pathological effect prevailing even in the complex GBM microenvironment, while tumor parenchymal signals may blunt the growth-promoting impact of humanin (which is therefore restricted to an *in vitro* condition).

Clinical and preclinical research has shown that GAMs are legitimate targets for adjuvant treatment of GBMs.[2,6,7,50] However, it also became clear that GAM-directed therapies require careful characterization of the tumor parenchyma and of GBM cells in order to stratify individuals for a specific treatment.[7,51–53] This study complies with these stringent criteria and outlines a preclinical strategy to identify humanin-sensitive GBMs for which GP130 inhibitors have an overarching, synergistic effect to support TMZ efficacy. Hence, this provides a basis to improve the standard of care in a large group of patients with GBMs.

Limitations of the study

In our GBM mouse models, we have recapitulated humanin signaling by orthotopically implanting humanin-sensitive GBM cells and partly infused nanomolar amounts of humanin (or vehicle). In other experiments, we overexpressed functionally released humanin isoforms (as compared to experiments with an inactive isoform). This was necessary in order to substitute for humanin expression in GAMs, which is lacking in mouse myeloid cells. Our bioassays with humanin-sensitive hGBM1-HN-WT cells suggested that humanin release from genetically manipulated GBMs was biologically adequate: humanin-sensitive GBMs respond specifically to nanomolar (but not higher) concentrations of humanin and the HN-WT expression in hGBM1 cells stimulated cell growth (hence was stimulated by nanomolar concentrations of humanin). Hence, recapitulating human-specific signaling in mice naturally faces some limitations, but these were carefully accounted for in the present work. Intravenous tracing, histopathology, and pharmacological experiments confirmed the multi-modal protumorigenic effects of humanin in GBMs and indicated a therapeutic solution. Altogether, this highlighted the pathological role of humanin signaling in neuropathology.

(B) Orthotopic hGBM1 was infused with HN (100 nM) or vehicle (artificial cerebrospinal fluid, aCSF) and i.p. injected with bazedoxifene-A (5 injections of BZA per week; 40 mg/kg; for 2 weeks) or vehicle; brains were labeled for HUS1; HUS1 expression was quantified.

(C) Mice with established, orthotopic HN-WT tumors received TMZ (50 mg/kg) and were cotreated with vehicle or BZA (as in B); after 3 weeks, tumor size was quantified (dashed line: average data from untreated WT GBMs).

(D) Mice with HN-WT GBMs received TMZ and were cotreated with vehicle or BZA (as in C); GBM samples were immunostained for active caspase-3 and immunolabeling was quantified (dashed line: average data from untreated WT GBMs).

(E) Intratumoral vascularization and vessel diameter were compared in HN-WT or HN-C8a tumors receiving TMZ.

(F) The HN-WT GBM mouse model was i.p. injected with TMZ and cotreated either with BZA or vehicle and the extent of intratumoral vascularization was compared. The number of biological replicates is indicated (dots in graphs indicate data from individual mice); all error bars are presented as mean ± SDM. Statistical significance is shown by one-way ANOVA (A, E), two-way ANOVA (B–D), or t test (F): *$p < 0.05$, **$p < 0.01$, ***$p < 0.001$, ****$p < 0.0001$; NS, not significant. Scale bars in (B, C) indicate 1 mm; scales in (D) represent 500 (overview) or 10 μm (magnified).

CellPress
OPEN ACCESS

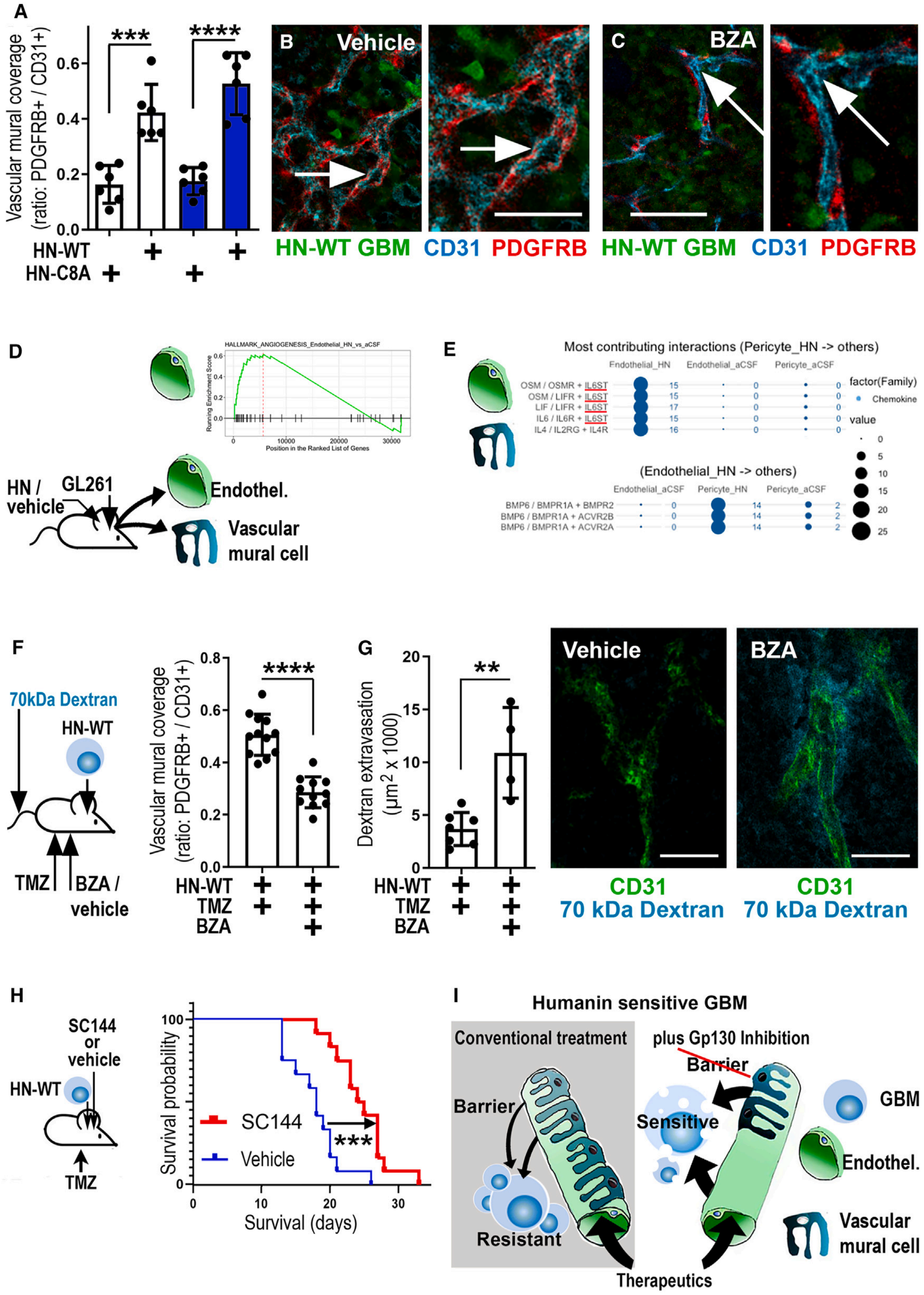

Figure 7. Humanin-mediated BTB formation is blunted by GP130 blockage increasing chemotherapeutic efficacy and survival

(A) Quantification of vascular mural coverage on tumor vessels in orthotopic HN-WT or HN-C8a GBMs with or without TMZ (indicated by blue bars).

(B and C) Vascular mural coverage of tumor vessels in TMZ-treated orthotopic HN-WT GBMs with or without BZA.

(legend continued on next page)

STAR★METHODS

Detailed methods are provided in the online version of this paper and include the following:

- KEY RESOURCES TABLE
- RESOURCE AVAILABILITY
  - Lead contact
  - Materials availability
  - Data and code availability
- EXPERIMENTAL MODEL AND SUBJECT DETAILS
  - Mice
  - Human glioblastoma specimens
  - Cell culture
- METHOD DETAILS
  - Transcriptomics
  - Proteomics
  - Q-RT-PCR
  - Genetic manipulation
  - Cell-counting
  - Immunodepletion
  - Cell cycle analysis
  - Western blotting
  - Mouse brain slice culture
  - *In vivo* experiments
  - Intracerebral drug application
  - Single cell preparation and fluorescence-activated cell sorting of intracerebrally HN-treated tumor vasculature
  - Dextran leakage assay
  - Histology and immunostaining
  - Immunofluorescence staining
  - Immunofluorescence staining for human GBMs samples
  - Immunohistochemistry for mouse samples
  - Microscopy
  - Tumor volume quantification
  - Quantification of tumor vasculature and pericye coverage
- QUANTIFICATION AND STATISTICAL ANALYSIS

SUPPLEMENTAL INFORMATION

Supplemental information can be found online at https://doi.org/10.1016/j.xcrm.2024.101658.

ACKNOWLEDGMENTS

Funding by the Deutsche Forschungsgemeinschaft (DFG) in the Priority Program, "Local and Peripheral Drivers of Microglial Diversity and Function" (SPP 2395) is gratefully acknowledged by C.F. (FL 1123/4-1) and R.G. (GL 691/8-1). R.G. and R.E.K. also acknowledge DFG grants GL 691/9-1 and GL691/2 and funding by the Anni Hofmann Stiftung, "Wilhelm Sander-Stiftung," and the "Verein zur Förderung von Wissenschaft und Forschung an der Medizinischen Fakultät der LMU München" (WiFoMed). DFG infrastructural support is acknowledged by R.G. (INST 409/223-1 FUGG) and A.I. (INST 86/2020-1). Valuable support by Veerakumar Balasubramaniyan and Andrea Schneider for flow cytometry and histological analysis is acknowledged.

AUTHOR CONTRIBUTIONS

J.C., M.L., E.M., H.K., A.I., I.F., K.P.L.B., C.F., R.E.K., and R.G. designed the experiments and/or conducted the experiments and analyzed the data; D.B., W.S., D.Z., G.L., S.Z., A.Y., B.D.V., and S.S.-K. conducted the experiments and analyzed the data; K.P.L.B., C.F., R.E.K., and R.G. wrote the manuscript; and all authors discussed, revised, and approved of the manuscript.

DECLARATION OF INTERESTS

The authors declare no competing interests.

Received: November 28, 2023
Revised: May 10, 2024
Accepted: July 2, 2024
Published: July 24, 2024

REFERENCES

1. Ostrom, Q.T., Price, M., Neff, C., Cioffi, G., Waite, K.A., Kruchko, C., and Barnholtz-Sloan, J.S. (2023). CBTRUS Statistical Report: Primary Brain and Other Central Nervous System Tumors Diagnosed in the United States in 2016-2020. Neuro Oncol. *25*, iv1–iv99. https://doi.org/10.1093/neuonc/noad149.
2. Aldape, K., Brindle, K.M., Chesler, L., Chopra, R., Gajjar, A., Gilbert, M.R., Gottardo, N., Gutmann, D.H., Hargrave, D., Holland, E.C., et al. (2019). Challenges to curing primary brain tumours. Nat. Rev. Clin. Oncol. *16*, 509–520. https://doi.org/10.1038/s41571-019-0177-5.
3. Aasland, D., Gotzinger, L., Hauck, L., Berte, N., Meyer, J., Effenberger, M., Schneider, S., Reuber, E.E., Roos, W.P., Tomicic, M.T., et al. (2019). Temozolomide Induces Senescence and Repression of DNA Repair Pathways in Glioblastoma Cells via Activation of ATR-CHK1, p21, and NF-κB. Cancer Res. *79*, 99–113. https://doi.org/10.1158/0008-5472.CAN-18-1733.
4. Yan, S., and Michael, W.M. (2009). TopBP1 and DNA polymerase alpha-mediated recruitment of the 9-1-1 complex to stalled replication forks: implications for a replication restart-based mechanism for ATR checkpoint activation. Cell Cycle *8*, 2877–2884. https://doi.org/10.4161/cc.8.18.9485.
5. Parrilla-Castellar, E.R., Arlander, S.J., and Karnitz, L. (2004). Dial 9-1-1 for DNA damage: the Rad9-Hus1-Rad1 (9-1-1) clamp complex. DNA Repair *3*, 1009–1014. https://doi.org/10.1016/j.dnarep.2004.03.032.

(D) Endothelial cells and pericytes were purified from a transgenic GBM mouse model (GL261, n = 11; infused with humanin, HN, or aCSF, control) and analyzed by transcriptomics; GSEA indicated enrichment for angiogenic traits in HN-stimulated endothelia (as compared to aCSF).
(E) Signaling pathways between endothelia and pericytes were analyzed from HN-infused versus control GBMs; in HN-infused GBMs, pericytes promote IL6ST (GP130) signaling in endothelia; in HN-infused GBMs, endothelia promote BMP signaling in pericytes.
(F) In HN-WT GBMs, receiving TMZ cotreatment with BZA reduced pericyte (PDGFRB+) coverage of tumor vessels ($CD31^+$; as compared to cotreatment with vehicle).
(G) Intravenous application of 70 kDA dextran as a tracer for vessel tightness showed that BZA-treated gliomas had significantly increased leakage (across $CD31^+$ vessels) into the tumor parenchyma, as compared to vehicle-treated mice.
(H) In mice with orthotopic HN-WT GBMs, intracerebral infusion of sc144 (10 μM) during TMZ chemotherapy prolonged survival as compared to intracerebral infusion of vehicle (n = 12 per group).
(I) Schematic summary: the BTB and DDR protect humanin-sensitive GBMs from TMZ. GP130 inhibitors reduce BTB tightness and blunt chemoresistance. Scale bars indicate 200 μm in (A) and 20 μm in (G). The number of biological replicates is indicated (dots in graphs indicate data from individual mice); all error bars are presented as mean ± SDM. Statistical significance is shown by one-way ANOVA (A), t test (F, G), or by Mantel-Cox test (F): *$p < 0.05$, **$p < 0.01$, ***$p < 0.001$.

CellPress
OPEN ACCESS

6. Audia, A., Conroy, S., Glass, R., and Bhat, K.P.L. (2017). The Impact of the Tumor Microenvironment on the Properties of Glioma Stem-Like Cells. Front. Oncol. *7*, 143. https://doi.org/10.3389/fonc.2017.00143.
7. Wei, J., Chen, P., Gupta, P., Ott, M., Zamler, D., Kassab, C., Bhat, K.P., Curran, M.A., de Groot, J.F., and Heimberger, A.B. (2020). Immune biology of glioma-associated macrophages and microglia: functional and therapeutic implications. Neuro Oncol. *22*, 180–194. https://doi.org/10.1093/neuonc/noz212.
8. Hambardzumyan, D., Gutmann, D.H., and Kettenmann, H. (2016). The role of microglia and macrophages in glioma maintenance and progression. Nat. Neurosci. *19*, 20–27. https://doi.org/10.1038/nn.4185.
9. Arvanitis, C.D., Ferraro, G.B., and Jain, R.K. (2020). The blood-brain barrier and blood-tumour barrier in brain tumours and metastases. Nat. Rev. Cancer *20*, 26–41. https://doi.org/10.1038/s41568-019-0205-x.
10. Jain, R.K., di Tomaso, E., Duda, D.G., Loeffler, J.S., Sorensen, A.G., and Batchelor, T.T. (2007). Angiogenesis in brain tumours. Nat. Rev. Neurosci. *8*, 610–622. https://doi.org/10.1038/nrn2175.
11. Armulik, A., Genove, G., and Betsholtz, C. (2011). Pericytes: developmental, physiological, and pathological perspectives, problems, and promises. Dev. Cell *21*, 193–215. https://doi.org/10.1016/j.devcel.2011.07.001.
12. Geraldo, L.H., Garcia, C., Xu, Y., Leser, F.S., Grimaldi, I., de Camargo Magalhaes, E.S., Dejaegher, J., Solie, L., Pereira, C.M., Correia, A.H., et al. (2023). CCL21-CCR7 signaling promotes microglia/macrophage recruitment and chemotherapy resistance in glioblastoma. Cell. Mol. Life Sci. *80*, 179. https://doi.org/10.1007/s00018-023-04788-7.
13. Mathivet, T., Bouleti, C., Van Woensel, M., Stanchi, F., Verschuere, T., Phng, L.K., Dejaegher, J., Balcer, M., Matsumoto, K., Georgieva, P.B., et al. (2017). Dynamic stroma reorganization drives blood vessel dysmorphia during glioma growth. EMBO Mol. Med. *9*, 1629–1645. https://doi.org/10.15252/emmm.201607445.
14. Sankowski, R., Bottcher, C., Masuda, T., Geirsdottir, L., Sagar, Sindram, E., Seredenina, T., Muhs, A., Scheiwe, C., Shah, M.J., et al. (2019). Mapping microglia states in the human brain through the integration of high-dimensional techniques. Nat. Neurosci. *22*, 2098–2110. https://doi.org/10.1038/s41593-019-0532-y.
15. Lee, C., Yen, K., and Cohen, P. (2013). Humanin: a harbinger of mitochondrial-derived peptides? Trends Endocrinol. Metabol. *24*, 222–228. https://doi.org/10.1016/j.tem.2013.01.005.
16. Tervaniemi, M.H., Katayama, S., Skoog, T., Siitonen, H.A., Vuola, J., Nuutila, K., Sormunen, R., Johnsson, A., Linnarsson, S., Suomela, S., et al. (2016). NOD-like receptor signaling and inflammasome-related pathways are highlighted in psoriatic epidermis. Sci. Rep. *6*, 22745. https://doi.org/10.1038/srep22745.
17. Huang, Y., Motta, E., Nanvuma, C., Kuhrt, L.D., Yuan, Y., Xia, P., Lubas, M., Zhu, S., Schnauss, M., Qazi, N., et al. (2022). Microglia/macrophage-derived human CCL18 promotes glioma progression via CCR8-ACP5 axis analyzed in humanized slice model. Cell Rep. *39*, 110670. https://doi.org/10.1016/j.celrep.2022.110670.
18. Volmar, M.N.M., Cheng, J., Alenezi, H., Richter, S., Haug, A., Hassan, Z., Goldberg, M., Li, Y., Hou, M., Herold-Mende, C., et al. (2021). Cannabidiol converts NF-κB into a tumor suppressor in glioblastoma with defined antioxidative properties. Neuro Oncol. *23*, 1898–1910. https://doi.org/10.1093/neuonc/noab095.
19. Kalin, R.E., Cai, L., Li, Y., Zhao, D., Zhang, H., Cheng, J., Zhang, W., Wu, Y., Eisenhut, K., Janssen, P., et al. (2021). TAMEP are brain tumor parenchymal cells controlling neoplastic angiogenesis and progression. Cell Syst *12*, 248–262.e247. https://doi.org/10.1016/j.cels.2021.01.002.
20. Mastrella, G., Hou, M., Li, M., Stoecklein, V.M., Zdouc, N., Volmar, M.N.M., Miletic, H., Reinhard, S., Herold-Mende, C.C., Kleber, S., et al. (2019). Targeting APLN/APLNR Improves Antiangiogenic Efficiency and Blunts Proinvasive Side Effects of VEGFA/VEGFR2 Blockade in Glioblastoma. Cancer Res. *79*, 2298–2313. https://doi.org/10.1158/0008-5472.CAN-18-0881.
21. Gong, Z., Tas, E., and Muzumdar, R. (2014). Humanin and age-related diseases: a new link? Front. Endocrinol. *5*, 210. https://doi.org/10.3389/fendo.2014.00210.
22. Metselaar, D.S., Meel, M.H., Benedict, B., Waranecki, P., Koster, J., Kaspers, G.J.L., and Hulleman, E. (2019). Celastrol-induced degradation of FANCD2 sensitizes pediatric high-grade gliomas to the DNA-crosslinking agent carboplatin. EBioMedicine *50*, 81–92. https://doi.org/10.1016/j.ebiom.2019.10.062.
23. Jia, Y., Swerdloff, R.S., Lue, Y., Dai-Ju, J., Surampudi, P., Cohen, P., and Wang, C. (2021). The IL-27 component EBI-3 and its receptor subunit IL-27Rα are essential for the cytoprotective action of humanin on male germ cells†. Biol. Reprod. *104*, 717–730. https://doi.org/10.1093/biolre/ioaa225.
24. Xu, S., Grande, F., Garofalo, A., and Neamati, N. (2013). Discovery of a novel orally active small-molecule gp130 inhibitor for the treatment of ovarian cancer. Mol. Cancer Therapeut. *12*, 937–949. https://doi.org/10.1158/1535-7163.MCT-12-1082.
25. Wightman, S.M., Alban, T.J., Chen, X., Lathia, J.D., Wang, Y., and Stark, G.R. (2021). Bazedoxifene inhibits sustained STAT3 activation and increases survival in GBM. Transl. Oncol. *14*, 101192. https://doi.org/10.1016/j.tranon.2021.101192.
26. Rose-John, S. (2018). Interleukin-6 Family Cytokines. Cold Spring Harbor Perspect. Biol. *10*, a028415. https://doi.org/10.1101/cshperspect.a028415.
27. Chen, Y., Zhou, J., Xu, S., and Nie, J. (2023). Role of Interleukin-6 Family Cytokines in Organ Fibrosis. Kidney Dis. *9*, 239–253. https://doi.org/10.1159/000530288.
28. Shi, Y., Guryanova, O.A., Zhou, W., Liu, C., Huang, Z., Fang, X., Wang, X., Chen, C., Wu, Q., He, Z., et al. (2018). Ibrutinib inactivates BMX-STAT3 in glioma stem cells to impair malignant growth and radioresistance. Sci. Transl. Med. *10*, eaah6816. https://doi.org/10.1126/scitranslmed.aah6816.
29. Filppu, P., Tanjore Ramanathan, J., Granberg, K.J., Gucciardo, E., Haapasalo, H., Lehti, K., Nykter, M., Le Joncour, V., and Laakkonen, P. (2021). CD109-GP130 interaction drives glioblastoma stem cell plasticity and chemoresistance through STAT3 activity. JCI Insight *6*, e141486. https://doi.org/10.1172/jci.insight.141486.
30. Brantley, E.C., and Benveniste, E.N. (2008). Signal transducer and activator of transcription-3: a molecular hub for signaling pathways in gliomas. Mol. Cancer Res. *6*, 675–684. https://doi.org/10.1158/1541-7786.MCR-07-2180.
31. Varga, A., Soria, J.C., Hollebecque, A., LoRusso, P., Bendell, J., Huang, S.A., Wagle, M.C., Okrah, K., Liu, L., Murray, E., et al. (2020). A First-in-Human Phase I Study to Evaluate the ERK1/2 Inhibitor GDC-0994 in Patients with Advanced Solid Tumors. Clin. Cancer Res. *26*, 1229–1236. https://doi.org/10.1158/1078-0432.CCR-19-2574.
32. Tajima, H., Niikura, T., Hashimoto, Y., Ito, Y., Kita, Y., Terashita, K., Yamazaki, K., Koto, A., Aiso, S., and Nishimoto, I. (2002). Evidence for in vivo production of Humanin peptide, a neuroprotective factor against Alzheimer's disease-related insults. Neurosci. Lett. *324*, 227–231. https://doi.org/10.1016/s0304-3940(02)00199-4.
33. Sabelstrom, H., Petri, R., Shchors, K., Jandial, R., Schmidt, C., Sacheva, R., Masic, S., Yuan, E., Fenster, T., Martinez, M., et al. (2019). Driving Neuronal Differentiation through Reversal of an ERK1/2-miR-124-SOX9 Axis Abrogates Glioblastoma Aggressiveness. Cell Rep. *28*, 2064–2079.e2011. https://doi.org/10.1016/j.celrep.2019.07.071.
34. Balmus, G., Lim, P.X., Oswald, A., Hume, K.R., Cassano, A., Pierre, J., Hill, A., Huang, W., August, A., Stokol, T., et al. (2016). HUS1 regulates in vivo responses to genotoxic chemotherapies. Oncogene *35*, 662–669. https://doi.org/10.1038/onc.2015.118.
35. Cen, L., Carlson, B.L., Pokorny, J.L., Mladek, A.C., Grogan, P.T., Schroeder, M.A., Decker, P.A., Anderson, S.K., Giannini, C., Wu, W., et al. (2013). Efficacy of protracted temozolomide dosing is limited in

MGMT unmethylated GBM xenograft models. Neuro Oncol. *15*, 735–746. https://doi.org/10.1093/neuonc/not010.

36. Kurzen, H., Schmitt, S., Naher, H., and Mohler, T. (2003). Inhibition of angiogenesis by non-toxic doses of temozolomide. Anti Cancer Drugs *14*, 515–522. https://doi.org/10.1097/00001813-200308000-00003.

37. Cheng, J., Korte, N., Nortley, R., Sethi, H., Tang, Y., and Attwell, D. (2018). Targeting pericytes for therapeutic approaches to neurological disorders. Acta Neuropathol. *136*, 507–523. https://doi.org/10.1007/s00401-018-1893-0.

38. Armulik, A., Genove, G., Mae, M., Nisancioglu, M.H., Wallgard, E., Niaudet, C., He, L., Norlin, J., Lindblom, P., Strittmatter, K., et al. (2010). Pericytes regulate the blood-brain barrier. Nature *468*, 557–561. https://doi.org/10.1038/nature09522.

39. Massenet-Regad, L., and Soumelis, V. (2024). ICELLNET v2: a versatile method for cell-cell communication analysis from human transcriptomic data. Bioinformatics *40*, btae089. https://doi.org/10.1093/bioinformatics/btae089.

40. Beets, K., Huylebroeck, D., Moya, I.M., Umans, L., and Zwijsen, A. (2013). Robustness in angiogenesis: notch and BMP shaping waves. Trends Genet. *29*, 140–149. https://doi.org/10.1016/j.tig.2012.11.008.

41. Silverman, S.L., Christiansen, C., Genant, H.K., Vukicevic, S., Zanchetta, J.R., de Villiers, T.J., Constantine, G.D., and Chines, A.A. (2008). Efficacy of bazedoxifene in reducing new vertebral fracture risk in postmenopausal women with osteoporosis: results from a 3-year, randomized, placebo- and active-controlled clinical trial. J. Bone Miner. Res. *23*, 1923–1934. https://doi.org/10.1359/jbmr.080710.

42. Jackson, S., ElAli, A., Virgintino, D., and Gilbert, M.R. (2017). Blood-brain barrier pericyte importance in malignant gliomas: what we can learn from stroke and Alzheimer's disease. Neuro Oncol. *19*, 1173–1182. https://doi.org/10.1093/neuonc/nox058.

43. Ruprecht, K., Kuhlmann, T., Seif, F., Hummel, V., Kruse, N., Bruck, W., and Rieckmann, P. (2001). Effects of oncostatin M on human cerebral endothelial cells and expression in inflammatory brain lesions. J. Neuropathol. Exp. Neurol. *60*, 1087–1098. https://doi.org/10.1093/jnen/60.11.1087.

44. Chang, J., Mancuso, M.R., Maier, C., Liang, X., Yuki, K., Yang, L., Kwong, J.W., Wang, J., Rao, V., Vallon, M., et al. (2017). Gpr124 is essential for blood-brain barrier integrity in central nervous system disease. Nat. Med. *23*, 450–460. https://doi.org/10.1038/nm.4309.

45. Chen, X., Momin, A., Wanggou, S., Wang, X., Min, H.K., Dou, W., Gong, Z., Chan, J., Dong, W., Fan, J.J., et al. (2023). Mechanosensitive brain tumor cells construct blood-tumor barrier to mask chemosensitivity. Neuron *111*, 30–48.e14. https://doi.org/10.1016/j.neuron.2022.10.007.

46. Butler, M., Pongor, L., Su, Y.T., Xi, L., Raffeld, M., Quezado, M., Trepel, J., Aldape, K., Pommier, Y., and Wu, J. (2020). MGMT Status as a Clinical Biomarker in Glioblastoma. Trends Cancer *6*, 380–391. https://doi.org/10.1016/j.trecan.2020.02.010.

47. Pena Agudelo, J.A., Pidre, M.L., Garcia Fallit, M., Perez Kuper, M., Zuccato, C., Nicola Candia, A.J., Marchesini, A., Vera, M.B., De Simone, E., Giampaoli, C., et al. (2023). Mitochondrial Peptide Humanin Facilitates Chemoresistance in Glioblastoma Cells. Cancers *15*, 4061. https://doi.org/10.3390/cancers15164061.

48. Moreno Ayala, M.A., Gottardo, M.F., Zuccato, C.F., Pidre, M.L., Nicola Candia, A.J., Asad, A.S., Imsen, M., Romanowski, V., Creton, A., Isla Larrain, M., et al. (2020). Humanin Promotes Tumor Progression in Experimental Triple Negative Breast Cancer. Sci. Rep. *10*, 8542. https://doi.org/10.1038/s41598-020-65381-7.

49. Zarate, S.C., Traetta, M.E., Codagnone, M.G., Seilicovich, A., and Reines, A.G. (2019). Humanin, a Mitochondrial-Derived Peptide Released by Astrocytes, Prevents Synapse Loss in Hippocampal Neurons. Front. Aging Neurosci. *11*, 123. https://doi.org/10.3389/fnagi.2019.00123.

50. Glass, R., and Synowitz, M. (2014). CNS macrophages and peripheral myeloid cells in brain tumours. Acta Neuropathol. *128*, 347–362. https://doi.org/10.1007/s00401-014-1274-2.

51. Rao, R., Han, R., Ogurek, S., Xue, C., Wu, L.M., Zhang, L., Zhang, L., Hu, J., Phoenix, T.N., Waggoner, S.N., and Lu, Q.R. (2022). Glioblastoma genetic drivers dictate the function of tumor-associated macrophages/microglia and responses to CSF1R inhibition. Neuro Oncol. *24*, 584–597. https://doi.org/10.1093/neuonc/noab228.

52. Li, J., Kaneda, M.M., Ma, J., Li, M., Shepard, R.M., Patel, K., Koga, T., Sarver, A., Furnari, F., Xu, B., et al. (2021). PI3Kγ inhibition suppresses microglia/TAM accumulation in glioblastoma microenvironment to promote exceptional temozolomide response. Proc. Natl. Acad. Sci. USA *118*, e2009290118. https://doi.org/10.1073/pnas.2009290118.

53. Pombo Antunes, A.R., Scheyltjens, I., Lodi, F., Messiaen, J., Antoranz, A., Duerinck, J., Kancheva, D., Martens, L., De Vlaminck, K., Van Hove, H., et al. (2021). Single-cell profiling of myeloid cells in glioblastoma across species and disease stage reveals macrophage competition and specialization. Nat. Neurosci. *24*, 595–610. https://doi.org/10.1038/s41593-020-00789-y.

54. Podergajs, N., Brekka, N., Radlwimmer, B., Herold-Mende, C., Talasila, K.M., Tiemann, K., Rajcevic, U., Lah, T.T., Bjerkvig, R., and Miletic, H. (2013). Expansive growth of two glioblastoma stem-like cell lines is mediated by bFGF and not by EGF. Radiology and oncology *47*, 330–337. https://doi.org/10.2478/raon-2013-0063.

55. Binda, E., Visioli, A., Giani, F., Trivieri, N., Palumbo, O., Restelli, S., Dezi, F., Mazza, T., Fusilli, C., Legnani, F., Carella, M., Di Meco, F., Duggal, R., and Vescovi, A.L. (2017). Wnt5a Drives an Invasive Phenotype in Human Glioblastoma Stem-like Cells. Cancer research *77*, 996–1007. https://doi.org/10.1158/0008-5472.CAN-16-1693.

56. Peña Agudelo, Pidre, M.L., Garcia Fallit, M., Pérez Küper, M., Zuccato, C., Nicola Candia, Marchesini, A., Vera, M.B., De Simone, E., Giampaoli, C., Amorós Morales, Gonzalez, N., Romanowski, V., Videla-Richardson, G.A., Seilicovich, A., and Candolfi, M. (2023). Mitochondrial Peptide Humanin Facilitates Chemoresistance in Glioblastoma Cells. Cancers *15*, 4061. https://doi.org/10.3390/cancers15164061.

57. Cerami, E., Gao, J., Dogrusoz, U., Gross, B.E., Sumer, S.O., Aksoy, B.A., Jacobsen, A., Byrne, C.J., Heuer, M.L., Larsson, E., Antipin, Y., Reva, B., Goldberg, A.P., Sander, C., and Schultz, N. (2012). The cBio cancer genomics portal: an open platform for exploring multidimensional cancer genomics data. Cancer discovery *2*, 401–404. https://doi.org/10.1158/2159-8290.CD-12-0095.

58. Gao, J., Aksoy, B.A., Dogrusoz, U., Dresdner, G., Gross, B., Sumer, S.O., Sun, Y., Jacobsen, A., Sinha, R., Larsson, E., Cerami, E., Sander, C., and Schultz, N. (2013). Integrative analysis of complex cancer genomics and clinical profiles using the cBioPortal. Science signaling *6*, l1. https://doi.org/10.1126/scisignal.2004088.

59. Noël, F., Massenet-Regad, L., Carmi-Levy, I., Cappuccio, A., Grandclaudon, M., Trichot, C., Kieffer, Y., Mechta-Grigoriou, F., and Soumelis, V. (2021). Dissection of intercellular communication using the transcriptome-based framework ICELLNET. Nature communications *12*, 1089. https://doi.org/10.1038/s41467-021-21244-x.

60. Bowman, R.L., Wang, Q., Carro, A., Verhaak, R.G., and Squatrito, M. (2017). GlioVis data portal for visualization and analysis of brain tumor expression datasets. Neuro-oncology *19*, 139–141. https://doi.org/10.1093/neuonc/now247.

61. Schindelin, J., Arganda-Carreras, I., Frise, E., Kaynig, V., Longair, M., Pietzsch, T., Preibisch, S., Rueden, C., Saalfeld, S., Schmid, B., Tinevez, J.Y., White, D.J., Hartenstein, V., Eliceiri, K., Tomancak, P., and Cardona, A. (2012). Fiji: an open-source platform for biological-image analysis. Nature methods *9*, 676–682. https://doi.org/10.1038/nmeth.2019.

62. Perez-Riverol, Y., Bai, J., Bandla, C., Garcia-Seisdedos, D., Hewapathirana, S., Kamatchinathan, S., Kundu, D.J., Prakash, A., Frericks-Zipper, A., Eisenacher, M., et al. (2022). The PRIDE database resources in 2022: a hub for mass spectrometry-based proteomics evidences. Nucleic Acids Res. *50*, D543–D552. https://doi.org/10.1093/nar/gkab1038.

CellPress
OPEN ACCESS

63. Humphrey, S.J., Karayel, O., James, D.E., and Mann, M. (2018). High-throughput and high-sensitivity phosphoproteomics with the EasyPhos platform. Nat. Protoc. *13*, 1897–1916. https://doi.org/10.1038/s41596-018-0014-9.

64. Bachmaier, S., Volpato Santos, Y., Kramer, S., Githure, G.B., Klockner, T., Pepperl, J., Baums, C., Schenk, R., Schwede, F., Genieser, H.G., et al. (2019). Nucleoside analogue activators of cyclic AMP-independent protein kinase A of Trypanosoma. Nat. Commun. *10*, 1421. https://doi.org/10.1038/s41467-019-09338-z.

65. Liu, T., Ma, W., Xu, H., Huang, M., Zhang, D., He, Z., Zhang, L., Brem, S., O'Rourke, D.M., Gong, Y., et al. (2018). PDGF-mediated mesenchymal transformation renders endothelial resistance to anti-VEGF treatment in glioblastoma. Nat. Commun. *9*, 3439. https://doi.org/10.1038/s41467-018-05982-z.

66. Al-Mayhani, T.F., Heywood, R.M., Vemireddy, V., Lathia, J.D., Piccirillo, S.G.M., and Watts, C. (2019). A non-hierarchical organization of tumorigenic NG2 cells in glioblastoma promoted by EGFR. Neuro Oncol. *21*, 719–729. https://doi.org/10.1093/neuonc/noy204.

STAR★METHODS

KEY RESOURCES TABLE

REAGENT or RESOURCE	SOURCE	IDENTIFIER
Antibodies		
Rat anti- CD31	Becton Dickinson	Cat#: 550274; RRID: AB_393571
Rabbit anti-Humanin	Thermo Fisher Scientific	Cat#: PA1-41325; RRID: AB_1957735
Rabbit anti-Humanin	Biorbyt	Cat# orb157596
Goat anti- PDGFR-b	R&D Systems	Cat#: AF1042; RRID: AB_2162633
Goat anti- Iba1	Abcam	Cat#: ab5076; RRID: AB_2224402
Mouse anti- IDH1-R132H	Dianova	Cat# DIA-H09, RRID:AB_2335716
Rabbit anti Laminin	Sigma	Cat# L9393, RRID:AB_477163
Mouse anti human-nuclei	Millipore	Cat# FCMAB306P, RRID:AB_10807437
Rabbit anti cleaved caspase3	Cell Signaling Technology	Cat# 9664; RRID:AB_2070042
Rabbit anti- HUS1	Thermo Fisher Scientific	Cat# PA5-109839, RRID:AB_2855250
Rabbit anti- Phospho-Histone H2A.X (Ser139)	Cell Signaling Technology	Cat# 9718, RRID:AB_2118009)
Biotinylated Donkey anti- rabbit IgG	Jackson ImmunoResearch	Cat# 711-065-152RRID: AB_2340593
Alexa Fluor 488 Donkey anti-rabbit IgG	Jackson ImmunoResearch	Cat#: 711-545-152; RRID: AB_2313584
Alexa Fluor 594 Donkey anti-rabbit IgG	Jackson ImmunoResearch	Cat#: 711-585-152; RRID: AB_2340621
Alexa Fluor 647 Donkey anti-rabbit IgG	Jackson ImmunoResearch	Cat#: 711-606-152; RRID: AB_2340625
Biotinylated Horse anti- goat IgG	Vector Laboratories	Cat#: BA-9500; RRID:AB_2336123
Biotinylated Donkey anti- goat IgG	Jackson ImmunoResearch	Cat#: 705-065-147 RRID: AB_2340397
Alexa Fluor 488 Donkey anti- goat IgG	Jackson ImmunoResearch	Cat#: 705-545-147; RRID: AB_2336933
Alexa Fluor 647 Donkey anti- goat IgG	Jackson ImmunoResearch	Cat#: 705-605-003; RRID: AB_2340436
Biotinylated Donkey anti- rat IgG	Jackson ImmunoResearch	Cat#: 12-055-153; RRID: AB_2340643
Biotinylated goat anti- rat IgG	Vector Laboratories	Cat# BA-9400, RRID:AB_2336202
Cy 5 conjugated Donkey anti-rat IgG	Jackson ImmunoResearch	Cat# 712-175-150; RRID: AB_2340671
Alexa Fluor 594 Donkey anti- rat IgG	Jackson ImmunoResearch	Cat#: 712-585-150; RRID: AB_2340688
Alexa Fluor 647 Donkey anti- rat IgG	Jackson ImmunoResearch	Cat#: 712-585-150; RRID: AB_2340688
Cy 2 conjugated Donkey anti-rat IgG	Jackson ImmunoResearch	Cat# 712-175-150; RRID: AB_2340673
Biotinylated Donkey anti-chicken IgG	Jackson ImmunoResearch	Cat# 703-065-155; RRID: AB_2313596
FITC conjugated Donkey anti-chicken IgG	Jackson ImmunoResearch	Cat# 703-095-155; RRID: AB_2340356
Alexa Fluor 488 conj. Streptavidin	Jackson ImmunoResearch	Cat#: 016-540-084; RRID: AB_2337249
Alexa Fluor 594-streptavidin conjugate	Jackson ImmunoResearch	Cat# 016-580-084; RRID: AB_2337250
Alexa Fluor 647-streptavidin conjugate	Jackson ImmunoResearch	Cat# 016-600-084; RRID: AB_2341101
Biotinylated Isolectin B4 (IB4)	Santa Cruz	Cat# sc-1205, RRID:AB_2155054
Rabbit anti- Akt	Cell Signaling Technology	Cat# 9272, RRID:AB_329827
Rabbit anti- phospho-AKT S473	Cell Signaling Technology	Cat# 4060, RRID:AB_2315049
Rabbit anti- ATR	Cell Signaling Technology	Cat# 2790, RRID:AB_2227860
Rabbit anti- phospho-ATR T1989	Cell Signaling Technology	Cat# 30632, RRID:AB_2798992
Rabbit anti- phospho-ERK1/2 T202/Y204	Cell Signaling Technology	Cat# 4370, RRID:AB_2315112
Rabbit anti- ERK1/2	Cell Signaling Technology	Cat# 9102 (also 9102L, 9102S), RRID:AB_330744
Rabbit anti- GAPDH	Cell Signaling Technology	Cat# 5174, RRID:AB_10622025
Rabbit anti- STAT3	Cell Signaling Technology	Cat# 4904, RRID:AB_331269
Rabbit anti- phospho-STAT3 Y705 (	Cell Signaling Technology	Cat# 9145, RRID:AB_2491009
Rabbit anti- HUS1	Cell Signaling Technology	Cat# 16416, RRID:AB_2798762
mouse Anti-a-actin	Sigma-Aldrich	Cat# A5441, RRID:AB_476744
Goat anti-Mouse IgG-HRP	GenDepot	Cat# SA201

(*Continued on next page*)

Continued		
REAGENT or RESOURCE	SOURCE	IDENTIFIER
and goat anti-Rabbit IgG-HRP	GenDepot	Cat# SA202
PE Mouse Anti-Human CD11b	BD Biosciences	Cat# 561001, RRID:AB_10563205
FITC Mouse Anti-Human CD45	BD Biosciences	Cat# 347463, RRID:AB_40030
Alexa Fluor 647 Rat Anti-Mouse CD31	BD Biosciences	Cat# 563608, RRID:AB_2738313
Chemicals, peptides, and recombinant proteins		
TSA Reagent, Biotin-XX Tyramide	Fisher Scientific	Cat# T20947
Dapi4′,6-Diamidin-2-phenylindole (DAPI)	Sigma-Aldrich	Cat# D 9564
X-tremeGENE HP DNA transfection reagent	Sigma-Aldrich	Cat# 6366244001
Tamoxifen	Sigma-Aldrich	Cat#: T5648
Tissue-Tec O.C.T	Sakura-Finetek	Cat#: 4583
Fluorescence Mounting Medium	Dako	Cat#: S3023
SYTOX Blue dead cell stain	ThermoFisher Scientific	Cat#: S34857
DMEM	Milipore	Cat#: FG0415
Dulbeccos MEM (10x)	Biochrom	Cat#: F0455
FBS superior	Biochrom	Cat#: F0615
DMEM F12	ThermoFisher Scientific	Cat#: 11320-074
B27-supplements	ThermoFisher Scientific	Cat#: 17504044
EGF	PeproTech	Cat#: 100-15
FGF	PeproTech	Cat#: 100-18B
TRIzol	ThermoFisher Scientific	Cat#: 15596026
QuantiTect Reverse Transcription Kit	Qiagen	Cat#: 205311
TaqMan™ Gene Expression Master Mix,	ThermoFisher Scientific	Cat#: 4370074
Bazedoxifene acetate (BZA)	Sigma-Aldrich	Cat#: PZ0018
Humanin	Biorbyt	Cat#: orb372420
Humanin G (HNG)	Designer BioScience	Cat#: H54838
SC144 hydrochloride	Sigma-Aldrich	Cat#: SML0763
AZ20	Tocris Bioscience	Cat#: 5198
Temozolomide	Sigma-Aldrich	Cat#: T2577
Clodronate Liposomes	Liposoma BV	Cat#: C-005
Corn Oil	Sigma-Aldrich	Cat#: C8267
7-AAD	BD Pharmingen	Cat#: 559925
Mirdametinib (PD0325901)	Selleckchem	Cat#:F1036
Ravoxertinib (RAV) GDC-0994	Selleckchem	Cat#: F7554
Dextran (Biotin) Lysine Fixable (70kDA)	ThermoFisher Scientific	Cat#: D1957
Oncostatin M (OSM) humn	Sigma-Aldrich	Cat#: O9635-
Leukemia Inhibitory Factor (LIF) human	MedChemExpress	Cat#: HY-P73276
Ciliary Neurotrophic Factor (CNTF) human	MedChemExpress	Cat#: HY-P7145
Interleukin-6 (IL6) human	Sigma-Aldrich	Cat#: H7416
Phalloidin-iFluor 488	Abcam	Cat#: ab176753
Critical commercial assays		
MaxFluor™ Mouse on Mouse Fluorescence Detection Kit (MaxFluor 488)	Dianova	Cat# MF01
Vectastain Elite ABC Kit (peroxidase standard) PK-6100	Vector Laboratories	Cat# PK-6100, RRID:AB_233681
CD109 TaqMan Genexpression Assay	ThermoFisher Scientific	Cat#: Hs00370347_m1
FPR1 TaqMan Genexpression Assay	ThermoFisher Scientific	Cat#: Hs00181830_m1
FPR2 TaqMan Genexpression Assay	ThermoFisher Scientific	Cat#: Hs00265954_m1
FPR3 TaqMan Genexpression Assay	ThermoFisher Scientific	Cat#: Hs01574392_m1

(*Continued on next page*)

Continued		
REAGENT or RESOURCE	SOURCE	IDENTIFIER
CNTFR TaqMan Genexpression Assay	ThermoFisher Scientific	Cat#: Hs00181798_m1
IL6ST TaqMan Genexpression Assay	ThermoFisher Scientific	Cat#: Hs00174360_m1
ACTB TaqMan Genexpression Assay	ThermoFisher Scientific	Cat#: Hs99999903_m1
Deposited data		
Raw and analyzed data of transcription profiling by array of TAM and total tumor cell samples	https://www.ebi.ac.uk/biostudies/arrayexpress/	ArrayExpress: E-MTAB-13219
Raw and analyzed data of transcription profiling by array of human GBM samples	https://www.ebi.ac.uk/biostudies/arrayexpress/	ArrayExpress: E-MTAB-13220
RNA-seq Raw and analyzed data of Humanin treatment on brain tumor cell lines	https://www.ebi.ac.uk/biostudies/arrayexpress/	ArrayExpress: E-MTAB-13225
Phosphoproteome raw data and analyzed data of Humanin treated brain tumor cells	https://www.ProteomeXchange.org	ProteomeXchange: PXD051404
Copy number variation Raw data of primary brain tumor cells	https://www.ebi.ac.uk/biostudies/arrayexpress/	ArrayExpress: E-MTAB-7649
Effect of Humanin treatment on brain tumor vascular cells	https://www.ebi.ac.uk/biostudies/arrayexpress/	ArrayExpress: E-MTAB-14064
Experimental models: Cell lines		
HEK293T/17(ATCC)	Cellosaurus	RRID:CVCL_1926)
GL261	National Cancer Institute, NCI-Frederick	RRID:CVCL_Y003 and Mastrella et al., 2019[20]
U87MG	Cellosaurus	RRID:CVCL_0022
NCH644 primary human GBM cells (called GBM-1 in this study)	Podergajs et al.[54]	RRID: CVCL_X914 and Mastrella et al., 2019[20]
NCH684primary human GBM cells (called GBM-2 in this study)	Kalin et al.[19]	Volmar et al., 2021[18]
GBM20 primary human GBM cells (called GBM-3 in this study)	Kalin et al.[19]	Volmar et al., 2021[18]
G0128 primary human GBM cells (called GBM-4 in this study)	this study	N/A
G0147 primary human GBM cells (called GBM-5 in this study)	this study	N/A
Line#2 primary human GBM cells (called GBM-6 in this study)	Binda et al.[55]	Volmar et al., 2021[18]
Line#10 primary human GBM cells (called GBM-7 in this study)	Binda et al.[55] and this study	N/A
Line#11 primary human GBM cells (called GBM-8 in this study)	Binda et al.[55] and this study	N/A
pluripotent stem cells (hiPSC-MG)		N/A
Experimental models: Organisms/strains		
Pdgfrb-cre/ERT2 Csln/J (here: *Pdgfrb*-creER)	The Jackson Laboratory	RRID:IMSR_JAX: 030201 and Kalin et al., 2021[19]
Gt(ROSA)26Sor-ACTB-2tdTomato-EGFP/Luo/J (here: lox-STOP-lox-tdTomato)	The Jackson Laboratory	RRID:IMSR_JAX: 007676 and Kalin et al., 2021[19]
C57BL6J	Charles River	Cat#: 632C57BL/6J
Hsd:Athymic Nude-Foxn1nu	Charles River	Cat#: 490CRATHHO
Recombinant DNA		
HN-WT-ORF plasmid (HN_WT-ORF_ pcDNA3.1(+)-P2A-eGFP)	Genscript	N/A
HN-mutant-L9R plasmid (HN_L9R-mutant_ pcDNA3.1(+)-P2A-eGFP)	Genscript	N/A
HN- mutant-C8A plasmid (HN_C8A-mutant_ pcDNA3.1(+)-P2A-eGFP)	Genscript	N/A

(*Continued on next page*)

Continued

REAGENT or RESOURCE	SOURCE	IDENTIFIER
Humanin shRNA lentiviral and non-target control constructs (Plasmid U6.shRNA.CMV.copGFP-2A-Puro.WPRE and U6.ctl shRNA.CMV.copGFP-2A-Puro.WPRE)	pUC57 vector with Humanin shRNA CCCGTGAAGAGGCGGGCATAAAA GTTCTCTTTATGCCCGCCTCTTC ACGGGTTTTTT (Packgene)	Peña Agudelo et al.[56]
HUS1 shRNA lentiviral and non-target control constructs	BioCat	Cat#: TLHSU1400-3364-pZIP-hCMV-ZsGreen-GVO-TRI
CD109 shRNA lentiviral and non-target control constructs	BioCat	Cat#: TLHSU1400-135228-GVO-TRI
psPAX2	Addgene	RRID:Addgene_12260
pMD2.G	Addgene	RRID:Addgene_12259
Software and algorithms		
AxioVision Imaging System	Zeiss	RRID:SCR_002677
bcl2Fastq	Illumina	RRID:SCR_015058
BD FacsDiva	BD Biosciences	RRID:SCR_001456
cbioportal (visualization, analysis and download of large-scale cancer genomics datasets)	http://www.cbioportal.org/; Cerami et al.[57] and Gao et al.[58]	RRID:SCR_014555
Chilibot: Gene and Protein relationships from MEDLINE	http://www.chilibot.net/	RRID:SCR_001705
DESeq2 (Software package for differential gene expression analysis)	https://bioconductor.org	RRID:SCR_015687
ClusterProfiler (Software R package for statistical analysis and visualization of functional profiles for genes and gene clusters.)	https://bioconductor.org	RRID:SCR_016884
enrichplot (*visualization of Functional Enrichment Result*)	https://bioconductor.org	https://doi.org/10.18129/B9.bioc.enrichplot
ICELLNET (Dissection of intercellular communication using the transcriptome-based framework ICELLNET)	https://github.com/soumelis-lab/ICELLNET	Noël et al.[59]
CLC Genomics Workbench	Qiagen	RRID:SCR_011853
CLC Sequence Viewer 8 on CLC Main Workbench	Qiagen	RRID:SCR_000354
http://designstudio.illumina.com/for Visualization/design of genetic code	Illumina	N/A
FCS Express	https://denovosoftware.com/?gclid=EAIaIQobChMI36rn3-Dd3AIV2ud3Ch27Iw2oEAAYASAAEgLbRvD_BwE	RRID:SCR_016431
FlowJo	https://www.flowjo.com/solutions/flowjo	RRID:SCR_008520
https://github.com/msquatrito/shiny_GlioVis	Bowman et al.[60]	N/A
https://imagej.net/Fiji	Schindelin et al.[61]	RRID:SCR_002285
SoftMax Pro Data Acquisition and Analysis Software	Molecular Devices	RRID:SCR_01424
Tecan i-Control	Tecan	RRID:SCR_024562
ViiA 7 Real-Time PCR System	Applied Biosystems	RRID:SCR_023358
Leica Application Suite X	https://www.leica-microsystems.com/products/microscope-software/details/product/leica-las-x-ls/	RRID:SCR_013673

RESOURCE AVAILABILITY

Lead contact

Further information and requests for resources and reagents should be directed to and will be fulfilled by the lead contact, Rainer Glass (rainer.glass@med.uni-muenchen.de).

Materials availability

This study did not generate unique reagents.

Data and code availability

- Raw and analyzed data of RNA-seq and transcription profiling by array are publicly available under the accession numbers E-MTAB-13219, E-MTAB-13220,E-MTAB-13225 and E-MTAB-14064. The mass spectrometry proteomics data have been deposited to the ProteomeXchange Consortium via the PRIDE[62] partner repository with the dataset identifier PXD051404.
- This paper does not report original code.
- Any additional information required to reanalyze the data reported in this paper is available from the lead contact upon request.

EXPERIMENTAL MODEL AND SUBJECT DETAILS

Mice

Animal experiments were carried out in compliance with the German law on animal welfare, and animal protocols were approved by local authorities ‘‘Regierung von Oberbayern’’ in Munich or the ‘‘Ministerium für Energiewende, Landwirtschaft, Umwelt, Natur und Digitalisierung des Landes Schleswig-Holstein (MELUND)’’ Kiel, Germany as required. Mice were housed in standardized cages in the animal centers of the Ludwig-Maximilians-University (LMU) Munich, the University of Kiel or the animal experiment center, received chow *ad libitum* and were kept under a circadian rhythm with 12 h light and dark cycles. ARRIVE guidelines were followed for all animal experiments. Mice used for experiments were of both sex and older than postnatal day 100. Tumor-take in all neuro-oncological models was R 98%; mice were only excluded from analysis when no tumor-growth observed. A comprehensive list of all transgenic mouse strains used for this study is given in the key resources table.

Human glioblastoma specimens

Glioblastoma samples were obtained from the Neurosurgery Department of the University Hospital, LMU Munich (Germany), from Charité university clinics (Berlin, Germany) and from the First Affiliated Hospital of Sun Yat-sen University (Guangzhou, China). This investigation received the endorsement of the Ethics Committees (under the project number 599-16) in Munich, in Berlin (under the license numbers EA112/2001, EA3/023/06 and EA2/101/08) and in Guangzhou (under the license number [2020]322).

Cell culture

Human, primary stem-like GBM cells hGBM-1 through −6 were cultured in DMEM-F12 medium supplemented with 1× B27, 10 ng/mL human EGF, 10 ng/mL human fibroblast growth factor (FGF), and 1% penicillin-streptomycin. The mouse GBMs cell line GL261 was cultured under adherent condition in Dulbecco’s Modified Eagle Medium (DMEM) containing 1× MEM non-essential amino acids, 10% fetal bovine serum (FBS), and 1% penicillin-streptomycin (100 units/ml penicillin and 100 μg/ml streptomycin). All cells were maintained in a 37°C humidified incubator with 95% O_2 and 5% CO_2. The culture medium was changed once or twice weekly according to the growth rate. GL261 cells were split with trypsin when the cells occupied over 80% of the culture flask. Human GBMs cell lines were passaged with accutase when big spheres formed.

METHOD DETAILS

Transcriptomics

Human GBMs samples and controls (patients with lateral sclerosis) were collected and split into single cell. The samples were stained with CD11b and CD45 and sorted using flow cytometry. $CD45^+$CD11b+ cells were collected from patients with GBMs and controls. Unlabeled cells (GBMs cells without $CD45^+$CD11b+ cells) were also collected from patients with GBMs. 10,000 cells were collected from each sample and stored at −80°C. For microarray hybridization, mRNA was isolated from samples and converted into cDNA; the cDNA was labeled with biotin. Later, the labeled and fragmented single strand cDNA was spiked with cDNA hybridization controls, which served as an internal control for sensitivity and accuracy of the hybridization reaction as well as the wash and staining procedure. The spiked cDNA samples were hybridized at 45°C for 16.5 h on separate Affymetrix GeneChip HuGene ST 2.0 Arrays. After hybridization, microarrays were stained in two binding cycles using anti-biotin antibodies and a streptavidin-phycoerythrin conjugate. Subsequently, the microarrays were washed with increasing stringency and conserved in holding buffer using the Affymetrix GeneChip 3000 Fluidics Station in combination with the Affymetrix GeneChip Command Console (AGCC) - Fluidics Control Software v4.0.0. Fluorescent signal intensities were detected with the Affymetrix GeneChip 3000 Scanner and AGCC Scan Control Software v4.0.0 (Affymetrix). Automatic grid was arranged and raw data were processed to generate image and intensity files by the AGCC software. The software tools AGCC Viewer v4.0.0 and Expression Console v1.4.1.46 (both Affymetrix) were used for visualization of the performance of microarray analysis.

hGBM-1 through −6 were used for RNA sequencing. The same number of cells for each cell line was plated under both the control and HN 200 nM conditions. After 72 h, cells were counted and cell pellets were gained, resuspended in 100 μL Trizol, and stored at −80°C. Samples were sent to single cell discoveries for bulk RNA sequencing.

For the analysis, the obtained data were first filtered to remove genes with no or nearly no expression of the indicated gene dataset across all samples. The resulting dataset contained 16329 genes. The data were then normalized and transformed using regularized log transformation, which normalizes the data with respect to library size and transforms the count data to the log2 scale in a manner that minimizes the differences between samples for genes with small counts. The normalized dataset was later used to assess sample similarities and to calculate the fold changes between samples of interest. For the comparisons without sample replicates, the fold difference for each gene was calculated in a pairwise fashion. The regularized log-transformed data were used to calculate pairwise differences in the log scale. Single-sample Gene Set Enrichment Analysis (ssGSEA) was applied to analyze cell cycle related genes, which were extracted from the molecular signature database under ontology gene sets (MSigDB, http://www.broadinstitute.org/gsea/msigdb) using the ssGSEA method of the R software Gene Set Variation Analysis (GSVA) package.

Proteomics

For the analysis of the Phosphoproteome, hGBM-1 cells were left untreated or were treated with 200 nM Humanin peptide for 15 min or 12 h. To obtain sufficient amount of proteins, cells were cultured under standard conditions (described above) to gain approx. 4 x 106 cells per condition (in triplicate). After treatment, cells were pelleted and directly frozen at −80°C. Next, samples were independently processed using the phosphoproteomics EasyPhos procedure.[63,64] In short, protein concentration was determined by BCA protein assay and samples were adjusted to equal concentrations. Sample preparation and mass spectrometry were exactly carried out as described by Humphrey et al.78. MaxQuant 2.4.14.0 was used to identify and quantify proteins and phosphopeptides with the following parameters: Database, Uniprot_UP000005640_Hsapiens_20231219; MS tol, 10ppm; MS/MS tol, 20ppm; Peptide FDR, 0.1; Protein FDR, 0.01 Min. peptide Length, 5; Variable modifications, Oxidation (M), Phosphorylation (ST); Fixed modifications, Carbamidomethyl (C); Peptides for protein quantitation, razor and unique; Min. peptides, 1; Min. ratio count, 2. For proteomic analysis, identified proteins were considered as statistically significant with FDR:0.05 and s0:1 (Two sample-test adjusted for multiple comparisons, Perseus). Phosphopeptide analysis was carried in Perseus.[63] Mass spectrometry phosphoproteomics data have been deposited to the ProteomeExchange Consortium via the PRIDE partner repository (https://www.ebi.ac.uk/pride/archive) with the dataset identifier PXD051404.[63,64]

Q-RT-PCR

Total RNA was extracted from cell pellets using TRIzol following Invitrogen's TRIzol RNA isolation protocol. cDNA synthesis was performed using the QuantiTect Reverse Transcription Kit (Qiagen) according to the manufacturers protocol with 1 μg total RNA. Obtained cDNA was diluted to 5 ng/μL and used for following qPCR analysis. Gene expression was analyzed using real-time PCR TaqMan Gene expression assay (Thermo Fisher Scientific). Each TaqMan Gene Expression Assay (the table below) consists of sequence-specific PCR primers and TaqMan assay-FAM dye-labeled MGB probe. PCR amplifications were performed in 20 μL total volume reactions containing10 μL TaqMan Gene Expression Master Mix, 2 μL cDNA templates, 7 μL nuclease free water, and 1 μL TaqMan assay probe. PCR reactions were run on StepOnePlus Real-Time PCR System with 45 cycles under standard Mode. The expression level of target genes was normalized by ACTB.

Genetic manipulation

CD109 and Hus1 shRNA lentiviral constructs were obtained from BioCat, including three different shRNA target sequences each and one no target control. Lentiviral packaging plasmid psPAX2(#12260) and VSV-G envelope expressing plasmid pMD2.G(#12259) were obtained from Addgene. For lentiviral packaging HEK293T/17(ATCC) cells were seed in 6 well plates and cultured with 2 mL 10% FBS DMEM medium under the above conditions. When the cells have grown to 60% confluence, replace the fresh medium and prepare the transfection mix. shRNA, psPAX2 and pMD2.G plasmids were prepared by mixing them at a ratio of 4:3:2 (0.4 μg shRNA plasmid+0.3 μg psPAX2+0.2 μg pMD2.G), serum-free DMEM was then added to the mixed plasmid DNA to a total volume of 250 μL. X-tremeGENE HP DNA transfection reagent (Roche, Switzerland) in a ratio of 3:1 plasmid mixture volume was added to the mixture in the previous step (2.7 μL transfection reagent +0.9 μg plasmid mix). Incubate at room temperature for 15 min (after which blurring of the solution can be observed). Spread transfection complex dropwise on the HEK293T/17 cells. The supernatant containing lentiviral particles was collected after 2 days transfection. The supernatant was centrifuged at 2000 rpm for 5 min to remove cell debris and aliquoted for storage at −80°C.

HN shRNA lentiviral is based on the shRNA sequence against Humanin hHN: CCCGTGAAGAGGCGGGCATAAAAG TTCTCTTTATGCCCGCCTCTTCACGGGTTTTTT[47] was synthesized fused to the U6 promoter, cloned into the bicistronic pUC57 vector containing GFP and puromycin under the CMV promoter and the lentivirus packaged (PackGene).

Lentiviral transduction: Human GBM cells were seed into 6-well plates, the same amount of lentiviral particles were added, incubated overnight, and washed with PBS for 3 times. The expression of fluorescence gene was observed under fluorescence microscope, and proper concentration of antibiotics (puromycin) was selected according to killing curve test to obtain stable knock down cell lines.

Cells were prepared as a single cell suspension and seeded at a density of 500,000 cells/2mL per well in 6-well plates. Transfection was performed using LipofectamineTM reagent, according to the manufacturer's protocol. The HN-WT plasmid (HN_WT-ORF_pcDNA3.1(+)-P2A-eGFP), HN-L9R plasmid (HN_L9R-mutant_pcDNA3.1(+)-P2A-eGFP) and HN-C8A plasmid (HN_C8A-mutant_pcDNA3.1(+)-P2A-eGFP) were transfected into hGBM cells. All three plasmids harbored the antibiotic (G418) resistance gene. To

select transfected cells, the antibiotic (G418) kill curve was tested for each cell line before transfection. Cells were selected and maintained in presence of G418.

Cell-counting

Wild type or genetically manipulated hGBMs were plated at a density of 100,000 cells (low density) or 1,000,000 cells (high density) in 2 mL growth factor-deprived medium (DMEM-F12 medium without EGF or FGF) in 6-well plates. Each group was prepared in triplicate. For the low-density group, the cells were counted 6 days after plating. For the high-density group, the cells were counted on day 2. Each experiment was independently repeated at least thrice. Previously, we confirmed that the GP130 inhibitor SC144 can block the protective effect of HN. For the experimental groups with SC144 200 nM treatment, the inhibitor was added on day 0.

To prepare conditioned medium hGBMs (expressing HN-WT, HN-C8A or HN-L9R) were seeded in plates at a concentration of 100,000 cells/ml in a growth factor-deprived medium. After 48 h, the cells were centrifuged at 400 G for 5 min, and the supernatant was collected. Wild type hGBMs (20,000) were plated in 500 μL conditioned medium in a 24-well plate. Three wells were prepared for each treatment group. The conditioned medium was changed every other day. The hGBM cell numbers were counted on day 7.

In additional experiments vehicle or SC144 (200 nM) was added to the conditioned medium. The effects of HN were modulated with BZA, AZ20 or Ravoxertinib (specific concentrations are outlined in the manuscript text): In experiments with BZA or Ravoxertinib hGBM cells were seeded at 100,000 cells per well in 2 mL medium on day 0, and vehicle bovine serum albumin (BSA)/HN (200 nM) and vehicle/BZA were added to the cells every other day. Cells were counted on day 7, and 100,000 cells were returned to the culture. Cells were counted again on day 14. The ATR inhibitor AZ20 was applied to 100,000 hGBMs cells in 2 mL medium (in 6-well plates on day 0) and treated with BSA/HN and vehicle/ATRi every other day. Cells were counted on day 6. The effect of AZ20 was also verified in HN-overexpressing cell line and HN-mutant cell lines.

Immunodepletion

HN-conditioned medium was obtained from hGBMs overexpressing the HN-WT peptide (medium was conditioned for 2 days). Immunodepletion (or generation of HN-containing controls) was performed using an HN antibody (or IgG istotype control) and protein A binding magnetic microbeads. First, the conditioned medium with HN antibody/IgG isotype control and 10%v/v protein A magnetic beads was incubated on ice for 30 min. The μ-columns were set on the MACS MultiStand and rinsed with 70% ethanol as elution buffer, followed by rinse with DMEM-F12 medium without growth factor. Then, the conditioned medium was removed from the ice and loaded onto μ columns. HN was removed from the filtered medium as it should combine with the HN antibody that is connected to the protein A beads attached to the column rather than flow through. hGBM1 cells were then cultured at a density of 40,000 cells/ml in 150μL of HN-depleted or control medium in pre-coated 96-well plates for 5 days. Three replicates were prepared for each experiment after 5 days of culture. The effect of immunodepletion was evaluated by cell quantification and immunostaining.

Cell cycle analysis

hGBM-1 cells were cultured in growth factor-free medium under the following conditions: 1) BSA, 2) BSA+TMZ 100 μM, 3) HN 200 nM, and 4) HN 200 nM + TMZ 100 μM, with 500,000 cells in 2 mL medium for each condition. Compounds were added every other day and the cells were cultured for 5 days. On day 5, the cells were collected and split into single cells using Accutase, followed by three times washes with ice-cold PBS. The cells were then fixed with ice-cold 70% ethanol at 4°C for 30 min. After fixation, the cells were centrifuged, and ethanol was discarded, followed by washing three times with PBS. Cells were filtered through a 40 μm strainer before resuspension in PBS. Then, 7-Amino-Actinomycin D (7-AAD) was added for staining of cell nuclei at the concentration of 5 μL (0.25 μg)/500 μL on cell suspension, and incubated for 10 min before analysis. Data acquisition was performed using BD Calibur at a low flow rate. FACS data analysis was performed using FCS Express software.

Western blotting

After electrophoresis, proteins in gel were transferred to nitrocellulose membrane in transfer buffer containing 15% methanol (Amersham) by Trans-Blot Turbo Transfer System (Bio-Rad). The membrane was washed three times with TBST (0.05% Tween 20), followed by blocked with 10% skim milk/TBST. After washing three times with TBST, the membrane was incubated overnight with primary antibodies diluted in 5% BSA/TBST. The membrane was washed three times with TBST, followed by incubated for 1 h at RT with HRP conjugated secondary antibodies (GenDepot) diluted 1:3000 times in 5% BSA/TBST. After washing four times with TBST, enhanced chemi-luminescent detection (GenDEPOT) of proteins was performed. The following primary antibodies were purchased from Cell Signaling Technologies; AKT (9272), phospho-AKT S473 (4060), ATR (2790), phospho-ATR T1989 (30632), ERK1/2 (9102), phospho-ERK1/2 T202/Y204 (4370), GAPDH (5174), STAT3 (4904), phospho-STAT3 Y705 (9145), HUS1 (16416). Anti-a-actin antibody (A5441) was purchased from Millipore-Sigma. Secondary antibodies, Goat anti-Mouse IgG-HRP (SA201) and goat anti-Rabbit IgG-HRP (SA202) were purchased from GenDepot.

Mouse brain slice culture

Brains from 14-day-old C57BL6N mice were obtained and sectioned into 250 μm slices for culture using vibratome as described previously. The mouse-originated microglia were depleted using clodronated liposomes, a procedure which was obtained over 48 h. 5000 Human microglia-like cells from induced pluripotent stem cells (hIPS-microglia) and/or 5000 hGBM-1 cells were

CellPress
OPEN ACCESS

inoculated into cultured, microglia-depleted mouse brain slices. Five groups were included in this experiment: 1) naive brain (no hIPS-microglia, no hGBM1), 2) hGBM1, 3) hIPS-microglia, 4) hGBM1 + hIPS-microglia, and 5) hGBM1 + hIPS-microglia + SC144. We analyzed brain slices from male and female mice in a 1:1 ratio. Mouse brain slices were cultured in medium (1 mL of DMEM with 10% heat inactivated FCS, 0.2 mM glutamine, 100 U/ml penicillin, and 100 mg/mL streptomycin for the initial culturing for 24 h, then changed to 50% DMEM with 25% of heat inactivated FCS, 25% of Hank's balanced salt solution, 50 mM sodium bicarbonate, 2% glutamine, 250 ng/mL insulin, 2.46 mg/mL glucose, 0.8 mg/mL vitamin C, 25 U/ml penicillin, 100 mg/mL streptomycin, and 5 mM Tris) for 5 days with a medium change every other day. Slices were then fixed with 4% PFA for 3 h. Samples were stored in 1× Tris-buffered saline at 4°C until use. Immunofluorescence staining of cultured mouse brain samples was performed as follow. First, the samples were washed with PBST (PBS+0.1%Tween 20) thrice (5 min for each time), and transferred to primary antibody which was diluted in dilution buffer (5% Donkey serum+0.3%Triton-100 in PBS). Samples were incubated at 4°C for 24 h, and then were washed thrice with 30 min each time. The secondary antibody with the fluorophore was incubated at room temperature for 4 h, followed by washing (3 × 30 min). Finally, the samples were stained with DAPI for 30 min and washed with PBS for 10 min.

In vivo experiments

All animal experiments were conducted according to the protocols of the local authorities and the regulations of the National Guidelines for Animal Protection, Germany. All animals were kept in Walter Brendel Center with sufficient food and water *ad libitum* in standard cages in a cabinet with 12 h light and dark cycle. Mice were examined daily and sacrificed when they were symptomatic or at specific time-points, depending on the experimental plan. The mice were anesthetized intraperitoneally (i.p.) with a mixture of 2% Rompun (0.3 mL), 10% ketamine (1.02 mL) and 0.9% NaCl (4.86 mL) at a dosage of 7 μL/kg. The mice were then disinfected on the head with 7.5% povidone-iodine and eye-protected with Bepanthen cream, and a midline incision was made on the skin above the skull. After stabilization of the stereotactic frame in the flat-skull position, a puncture was carefully and gently made on the skull with a 21G needle at the coordinate of 1 mm anterior and 2 mm right to the bregma. The needle of a 22G Hamilton syringe was rinsed thoroughly with decreasing concentrations of ethanol (99%, 70%, and 50%), sterilized distilled water, and 1×PBS before taking tumor cells. Tumor cells were prepared at a density of 100,000 cells/μL in culture medium. 1 μL cell suspension was injected into each mouse 3 mm under the skull in 2 min. The needle was slowly withdrawn at 1 mm/min after injection. The inoculated mice were then sutured and returned to the cages.

Intracerebral drug application

One day before the operation, artificial cerebrospinal fluid (aCSF) was prepared as follows: 1) solution A was prepared by mixing 500 mL sterile water, 8.66 g NaCl, 0.244 g KCl, 0.206 g CaCl2 · 2H2O, and 0.163 g MgCl2 · 6H2O; 2) solution B was prepared by dissolving 0.214 g Na2HPO4·7H2O and 0.027 g NaH2PO4 · H2O in 500 mL sterile water; 3) solutions A and B was combined in a 1:1 ratio and the solution was filtered through a 0.22 μm filter. Mini-pumps (Alzet mini-osmotic pump model 2004, 0.25 μL per hour, lasts around 28 days) were filled with either 200 μL aCSF or 100 nM HN dissolved in aCSF. All prepared mini-pumps were pre-warmed by immersion in aCSF under 37°C for overnight. After hGBM-1 cell inoculation, mini-pumps were directly installed by gently pushing the pump under the skin of the backs of mice and stabilizing the needle into the puncture point.

BZA was dissolved in 10%v/v dimethyl sulfoxide (DMSO) and 90%v/v corn oil i.p. at 4 mg/kg or 40 mg/kg in mice. BZA was injected from day 7 after tumor inoculation and was applied five times a week until the end of the experiment. The control group was injected with the vehicle.

TMZ was dissolved in 5%v/v DMSO and 95%v/v saline at a concentration of 5 mg/mL, and was maintained on a shaker until use. Mice were injected i.p. with 50 mg/kg from day 7 after tumor inoculation. The control group was injected with the vehicle. The exact injection schedules are introduced according to each experiment in the results section.

Single cell preparation and fluorescence-activated cell sorting of intracerebrally HN-treated tumor vasculature

Transgenic *PDGFRB*^{RFP} reporter (*Pdgfrb*-creER, lox-STOP-lox-tdTomato)[19] mice were inoculated with mouse GBM cells (GL261, 1x10^{5} cells/1 μL) and intracerebral infusion with Humanin (200 nM) via minipump or aCSF was performed. This experimental set-up was necessary to obtain pure cell fractions from GBM models. While endothelial cells can be purified from glioma by flow cytometry for CD31^{+} cells, separation of endothelia from pericytes in glioma is reliably achieved with PDGFRB^{RFP} mice. The reason for this is that surface markers for pericytes (required for FACS) become ambiguous throughout gliomagenesis, e.g., since endothelia strongly express the pericyte marker PDGFRB[65] or since glioma cells express the pericyte marker NG2.[66] However, it is possible to specifically induce RFP expression in intratumoral pericytes by genetic recombination of PDGFRB^{RFP} mice before the onset of tumorigenesis with Tamoxifen application (75 mg/kg body weight) injection one week before tumor inoculation). This labels pericytes in the tumor free brain (Angiogenesis. 2017 Nov; 20(4): 655–662) and the progeny of these recombined pericytes remains specifically labeled in the tumor bearing brain. At 14DPO mice were sacrificed, brains were harvested and the tumor mass was microdissected under a fluorescence-stereomicroscope (Leica). Single cells were obtained by triturating dissected GBM in PBS and subsequent treatment with 1 mg/ml collagenase-I followed by several wash-steps and filtering through a 40mm cell strainer.

FACS sorting was done using a MoFlo Astrios EQ,(Beckman Coulter) equipped with 7 Lasers (355nm, 405nm, 488nm, 532nm, 561nm, 592nm, 642nm) and operated with a 70mm Nozzle at 88kHz on single cell sort mode. Cells were pregated using FSC/SSC debris

exclusion, FSC-W singlet discrimination and bulk sorted for living (excluding SYTOX Blue dead stained cells; ThermoFischer Scientific) RFP+ tumor-derived vascular pericytes (n is 4 HN-treated versus 3 aCSF control animals) or AF647-CD31^{+} stained (isotype-controlled) vascular endothelial cells (n is 2 animals per group). Collected cells were resuspended in 100 μL Trizol, and stored at −80°C. Samples were sent to Genewiz Germany GmbH/Azenta Life Sciences for bulk RNA sequencing.

DESeq2 was used to obtain differentially expressed genes (DEGs; with |log2FC|> 1 and an adjusted *p*-value <0.05 as cut-off criteria). GSEA and GO analysis were performed with the R package ClusterProfiler (4.7.1), visualization of functional enrichment was performed by Enrichplot (1.18.3). To explore the cell-cell interaction in vascular cells, we performed cell communication analysis using ICELLNET.

Dextran leakage assay

Vascular permeability in brain tumors was determined by intravenous injection of 100 μL per mouse of a 70 kDa size lysine-fixable Dextran (biotin-labeled)at 1% in saline 15 min before sacrifice and transcardiac perfusion with 4% paraformaldehyde (PFA). The brains were then removed from the skull and post-fixed in 4% PFA for 48 h, washed in 1x PBS and immersed in 30% sucrose in 1x PBS solution until sunken to the bottom. From the frozen brains, 40 μm horizontal sections were made using a sliding microtome. After immunofluorescence staining against CD31 pictures were made on a TCS SP8 microscope (Leica). Per mouse four pictures at 40× magnification on three sections (12 in total) were made with the same laser settings. For the analysis of leakage of the dextran into the brain parenchyma the sections ware photographed on 40 objective by tile-scan function with 16 fields on a TCS SP8 microscope (Leica). Dextran leakage into the brain parenchyma was obtained by subtracting the CD31 area from the dextran area (stained by AF647-streptavidin) using ImageJ.

Histology and immunostaining

mice were anesthetized with Narcoren and perfused with PBS, followed by 4% PFA solution. Brains and other organs of interest were collected and immersed in 4% PFA at 4°C for 24 h for post-fixation. The organs were then transferred to hypertonic sucrose solution (30% w/v in 1× PBS). The organs were embedded in Cryomatrix and frozen in the vapor phase of liquid nitrogen after achieving the same osmotic pressure as the sucrose solution. The brains were cut into 40 μm sections using a microtome and placed in cryoprotective liquid (with 25% glycerol, 25% ethylene glycol and 50% 1×PBS) for later use. The sectioned samples were stored in a 24-well plate and maintained at −20°C in a freezer.

For immunocytochemistry, round glass (diameter: 12 mm) coverslips were placed in a 24-well plate (and covered with 500 μL poly-*d*-lysine (50 μg/mL) in each well for 1 h at room temperature. After coating, the coverslips were rinsed twice with sterilized water and left to air-dry for 1 h. hGBMs were split into single cell and plated at a density of 250,000 cells in 500μL growth factor-deprived medium per well. Alternatively, 8-well ibidi plates were used coated with 300 μL poly-*d*-lysine and 20′000 cells per well were applied. The coverslips with adherent cells were gently rinsed with PBS and fixed with 4% PFA. The attached cells were then subjected to immunofluorescence staining.

The samples of interest were mounted on slides and promptly dried. H&E staining was performed in glass wares as follows: 1) the slides were immersed in 100% ethanol for 30 s for dehydration; 2) the sections were then transferred to Mayer's hematoxylin solution for nuclei dye for 2 min; 3) the sections were rinsed with running distilled water for 5 min; 4) then, the sections were moved to 0.5% eosin solution for cytoplasm staining for 30 s; 4) briefly, the slides were rinsed in distilled water and transferred to increasing concentrations of ethanol for dehydration (1 min in 70%, 96% and 100% ethanol, respectively); 5) then the slides were transferred to Roti-Histol for 1 min; 6) Entellan was mounted on each slide before covering the coverslips; and 7) the stained sections were allowed to dry under the hood. Images for H&E staining were captured using Carl Zeiss Axioskop 2 microscope with Axiovision Rel. 4.9 software.

Immunofluorescence staining

The brain samples of interest were collected from cryoprotective liquid and placed in washing buffer PBST in a 12-well plate and washed for thrice with 5 min each time. This step was to rinse off the remaining cryoprotective reagent before staining. The samples were then transferred to blocking solution (5% normal Donkey serum in 0.3% Triton X-100) for 1 h at room temperature. After blocking, the brain sections were directly transferred to the primary antibody solution and incubated at 4°C overnight. On the second day, the brain samples were rinsed thrice in washing buffer with 5 min each time before being transferred to a secondary antibody. Incubation was then performed at room temperature for 2 h, followed by a washing step, as described previously. The sections were then mounted on slides and air dried for 15 min. After the nuclei staining with DAPI, the slides were washed by rinsing in distilled water shortly. Finally, the slides were mounted with fluorescence mounting medium and covered with coverslips. As the cell samples were already on the coverslip, the mounting medium was dropped onto the slides, and the round coverslip with cells attached on it was covered on slides with the cell side facing the slide.

Immunofluorescence staining for human GBMs samples

Human paraffin-embedded samples were processed with the following steps for deparaffinization and antigen retrieval before staining: The tissue sections were immersed in Histol for 10 min at room temperature for deparaffinization. The samples were immersed in ethanol with decreasing gradients (100%, 96%, 70%, and 50%), each step for 30 s, fixed in 70% Aceton at −20°C for 10 min, washed with PBST (thrice with 5 min). The samples were then cooked at 100°C in Citrat Bufffer (1.8 mM Citric acid and 8.2 mM

CellPress
OPEN ACCESS

tri-Natriumcitrat-Dihydrat, adjusted to PH 6.0 with 2 mM NaOH) for 20 min, cooled for 20 min to room temperature, followed by washing thrice with PBST (5 min) each time and processed as described above.

Immunohistochemistry for mouse samples

Paraffin-embedded mouse xenograft samples were first subjected to deparaffinization and antigen retrieval. Endogenous peroxidase activity was blocked with 3% hydrogen peroxide for 15 min at room temperature. The slides were then rinsed and blocked with 10% Donkey serum in PBS for 30 min the primary antibody was incubated for 1 h at room temperature, followed by 30 min incubation with a biotinylated secondary antibody. Finally, the sections were labeled with avidin-biotin-peroxidase for 30 min. The washing steps (thrice, 5 min each time) were applied between each antibody incubation. Signal visualization was achieved by incubation in 3,3′-diaminobenzidine (DAB) solution until the desired stain intensity developed. The samples were rinsed with tap water to prevent further signal development.

Microscopy

A Zeiss Axioskop-2 light microscope was used to perform imaging for H&E staining and immunohistochemistry. Evaluation of HUS1 fluorescence staining from immunodepletion experiments was performed using an Axio Observer A1 inverse fluorescence microscope. All other fluorescence staining was imaged using Leica confocal laser microscope SP8 confocal. A 20× objective with glycerol immersion was used for most quantifications. A 40× objective with glycerol immersion was used for higher magnification. All formats were set to 1024 × 1024 with a scan speed of 200–400 Hz. All channels were imaged separately to avoid crosstalk. Navigator was used when the overview was required. All images were adjusted according to the negative controls. Zoom-in was used in the region of interest. Tile scan was performed for the area that required more information from different layers of the sample to better visualize and capture the structure. Confocal images were later processed with LAS X (Leica) for further adjustment and export.

Tumor volume quantification

Mouse brains were cut horizontally for tumor volume quantification. Sections with tumor were collected every 0.4 mm in the dorsoventral axis (z axis), mounted, and stained with H&E. Later, the tumor area (A) in each section was measured using Axiovision Rel. 4.9 software. The tumor volume(V) was calculated as $V = ((Z\_top - Z\_bottom) \times (A\_top + \cdots + A\_bottom)) \div n$, where Z is the section coordinate relative to the bregma in the atlas and n is the number of sections with tumors. HUS1 staining was performed on slides attached with cells. Three images were obtained with a 40× objective using confocal microscopy for each cell line. Subsequently, the images were imported into ImageJ software for intensity quantification. Images were first converted to an 8-bit format, then the stained area was selected in ‘‘Image-Adjust-Threshold’’. The mean intensity value was obtained by selecting ‘‘Analyze-Measure’’.

Quantification of tumor vasculature and pericye coverage

Mouse sections stained with CD31 and PDGRB were photographed to quantify vessel length and density as well as pericyte coverage within the tumor area. For each mouse, three or four sections with good quality containing a tumor were prepared, and nine 40× magnification images per section were taken using a TCS SP8 microscope. Vessel length density was analyzed using AngioTool 0.6 software. Pericyte coverage was obtained by the ratio of PDGFRB+/CD31+ costained area of all $CD31^+$ array by ImageJ.

QUANTIFICATION AND STATISTICAL ANALYSIS

No statistical methods were used to predetermine sample sizes, but our sample sizes are similar to those previously reported[18,20]; experimental groups were not blinded. In all Figures, the data presented are representative of at least 3 independent experiments. Data-distribution was presented by mean - values and standard deviation of the mean; numbers of independent experiments or individual animals was indicated in the figures, legends or in the manuscript text. Student’s t, one-way/two-way ANOVA with Tukey post-hoc test or ANOVA with Bonferroni correction were used as indicated; in survival experiments, Kaplan–Meier curves were used and Log rank (Mantel-Cox) test was applied to determine statistical significance; primary endpoint was development of neurological symptoms clearly indicative of hGSC. p values are indicated as $^*p < 0.05$, $^{**}p < 0.01$, $^{***}p < 0.001$, and $^{****}p < 0.0001$ in all results. All statistical analyses were conducted using Graph Pad Prism 5.

Cell Reports Medicine, Volume *5*

Supplemental information

Myeloid cells coordinately induce glioma cell-intrinsic and cell-extrinsic pathways for chemoresistance via GP130 signaling

Jiying Cheng, Min Li, Edyta Motta, Deivi Barci, Wangyang Song, Ding Zhou, Gen Li, Sihan Zhu, Anru Yang, Brian D. Vaillant, Axel Imhof, Ignasi Forné, Sabine Spiegl-Kreinecker, Nu Zhang, Hiroshi Katayama, Krishna P.L. Bhat, Charlotte Flüh, Roland E. Kälin, and Rainer Glass

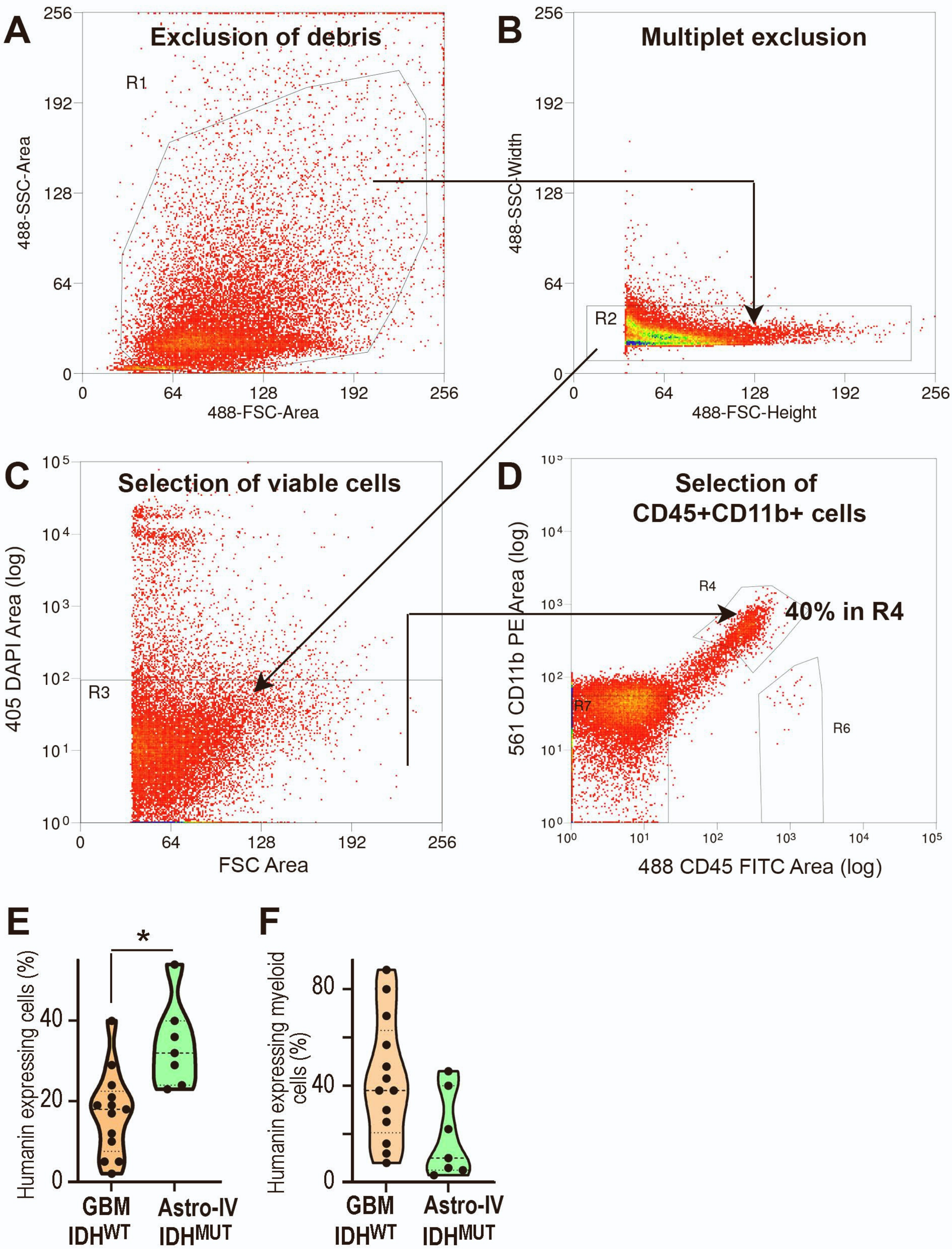
A
Exclusion of debris
R1
488-SSC-Area
488-FSC-Area
B
Multiplet exclusion
R2
488-SSC-Width
488-FSC-Height
C
Selection of viable cells
R3
405 DAPI Area (log)
FSC Area
D
Selection of CD45+CD11b+ cells
R4
40% in R4
R6
R7
561 CD11b PE Area (log)
488 CD45 FITC Area (log)
E
*
Humanin expressing cells (%)
GBM IDHWT
Astro-IV IDHMUT
F
Humanin expressing myeloid cells (%)
GBM IDHWT
Astro-IV IDHMUT

Figure S1: Flow cytometric gating strategy for purification of GBM associated myeloid cells (GAM). Related to Figure 1. Brain specimen (from GBM resection or epilepsy surgery) were dispersed into single cell suspensions, which were immunostained (for CD11b, CD45) and analyzed by flow cytometry. The strategy for the acquisition of FACS data is outlined: Debris was excluded **(A)** and analysis was restricted to single **(B)**, viable cells **(C)** with sound immunofluorescence signals (**D**; as compared to negative controls); the plot provides representative data of 13 independent FACS experiments and shows that viable myeloid cells (R4 gate) were readily purified from human specimen. **(E)** Quantification of Humanin-positive cells in IDH^{MUT} vs. IDH^{WT} tumors. **(F)** Preponderance of Humanin in GAMs of IDH^{WT} tumors is subject to inter-patient heterogeneity. Statistical significance is shown by ttest (*p < 0.05) in (**E**).

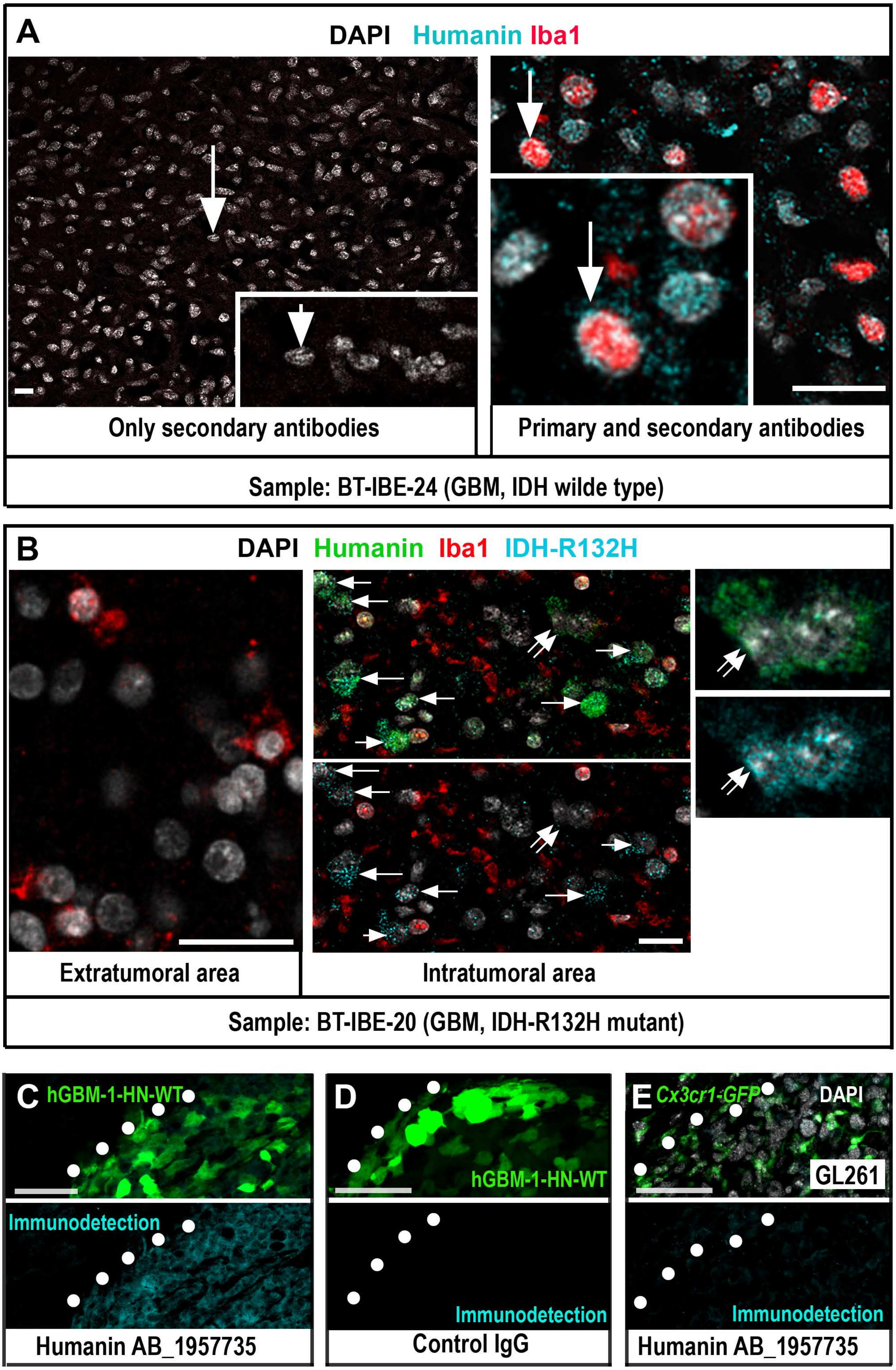
A
DAPI Humanin Iba1
Only secondary antibodies
Primary and secondary antibodies
Sample: BT-IBE-24 (GBM, IDH wilde type)
B
DAPI Humanin Iba1 IDH-R132H
Extratumoral area
Intratumoral area
Sample: BT-IBE-20 (GBM, IDH-R132H mutant)
C hGBM-1-HN-WT
Immunodetection
Humanin AB_1957735
D
hGBM-1-HN-WT
Immunodetection
Control IgG
E Cx3cr1-GFP
DAPI
GL261
Immunodetection
Humanin AB_1957735

Figure S2: Humanin is specifically detected in human GBM or astrocytoma cells and in GBM associated myeloid cells (GAM). Related to Figure 1. Samples from GBM patients **(A-B)** or mouse glioma models **(C-E)** were immunostained with or without Humanin antibody and the signal was analyzed by confocal microscopy. (**A)** A GBM specimen underwent the immunolabeling procedure (plus DAPI staining) with or without application of (primary) antibodies for Humanin and the myeloid marker Iba1. Incubation with seconday antibodies alone resulted in absence of immunostaining (while the DNA intercalating dye DAPI still labeled nuclei; arrow). Sequential application of primary and secondary antibodies resulted in immunolabeling for Iba1 or Humanin; partly cells were co-stained for both markers (arrow); the insets show enlargements of the cells marked by an arrow. (**B)** In IDH1-mutant (IDH1^{R132H}), grade-IV astrocytomas extra- and intra-tumoral areas were identified by IDH^{R132H} immunostaining. Colabeling for Humanin Iba1 and IDH^{R132H} was performed. To improve visibility of markers the central panel gives two views on the same samples: The upper part depicts labeling for all three markers; the lower part hides the Humanin channel. The arrows indicate IDH^{R132H}-positive GBM cells expressing Humanin (a magnification of the tumor cell marked by the double arrow is shown on the right). (**C, D**) Immunodetection for Humanin in an orthotopic mouse xenograft model with hGBM-1-HN-WT cells (expressing GFP). (C) Immunolabeling for Humanin (by the Humanin specific antibody AB_1957735) is confined to the tumor area in (dotted line) and was absent (D), in the same model, when using a non-immune IgG (control IgG, of the same isotype as the Humanin directed antibody). **(E)** Incubating samples from orthotopic murine gliomas (GL261) in *Cx3CR1-GFP* transgenic mice with AB_1957735 resulted in absence of immunolabeling. Scales bars indicate 30 µm (**A, B**), and 50 µm (**C-E**).

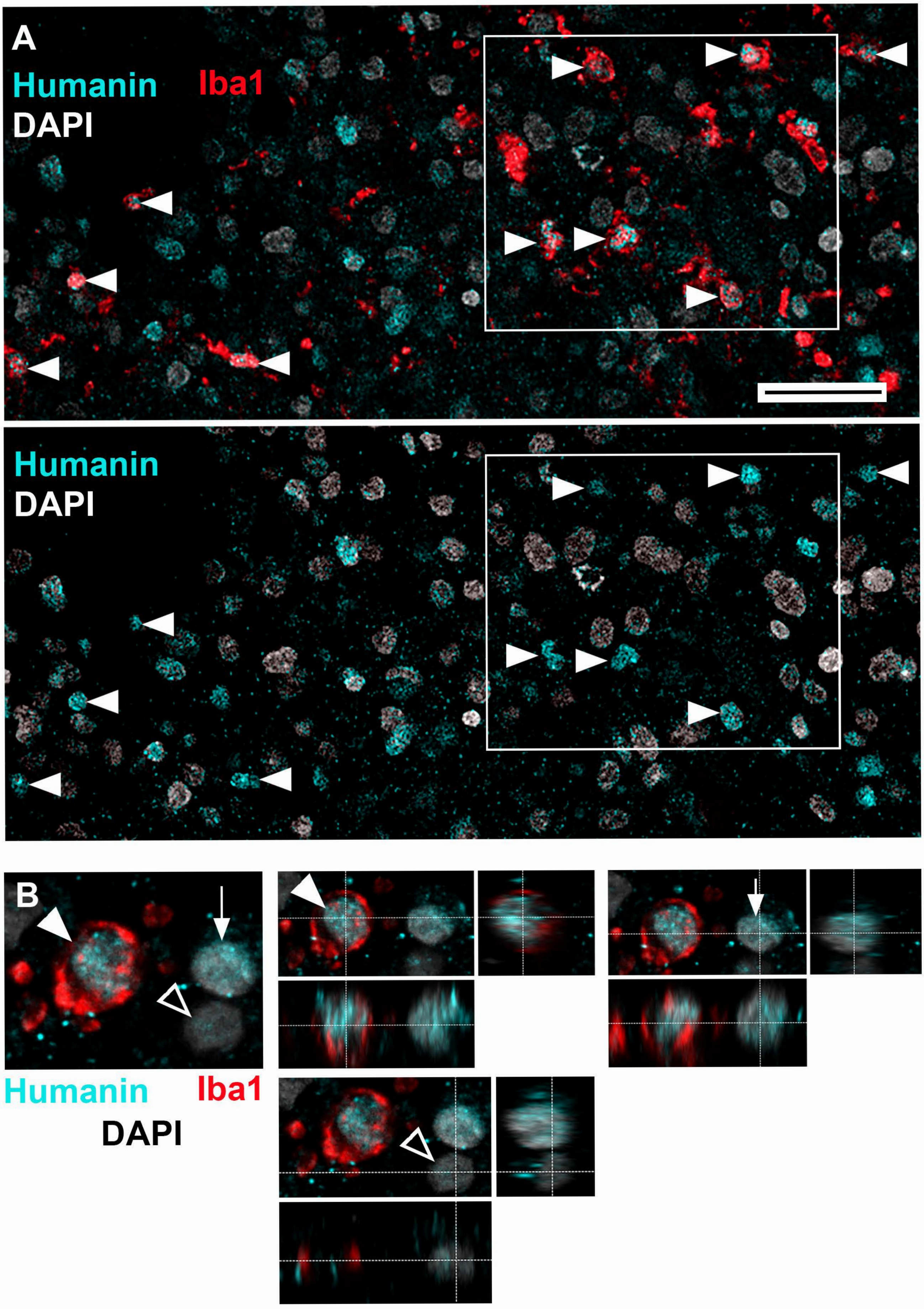
A
Humanin
Iba1
DAPI
Humanin
DAPI
B
Humanin
Iba1
DAPI

Figure S3: Humanin is broadly expressed in GBM associated myeloid cells (GAM) of human GBM specimen. Related to Figure 1. (**A)** Confocal micrograph from a patient specimen (nuclei in gray; size is representative for a quarter counting frame in our immunoquantification procedure, relating to Figure S2) co-immunostained for Humanin (cyan) and Iba1 (red). Cells double-positive for Humanin and Iba1 are marked by arrowheads; the insets correspond with Figure 1C. (**B)** This single optical section shows a representative example of an individual GAM (white arrowhead) co-stained for Humanin (cyan) and Iba1 (red) and an intra-tumoral cell (arrow) positive for Humanin only; furthermore, a cell (nucleus in grey) that is negative for Humanin or Iba1 (black arrowhead) is presented. Confocal cross hair inspection of optical Z-stacks confirmed Humanin expression in Iba1-positive GAM (arrowhead), Humanin single positive cells (arrow) or absence of Humanin/Iba1 in the cell marked with a black arrowhead .Scales bars indicate 50 µm (**A**), or 10 µm (**B**).

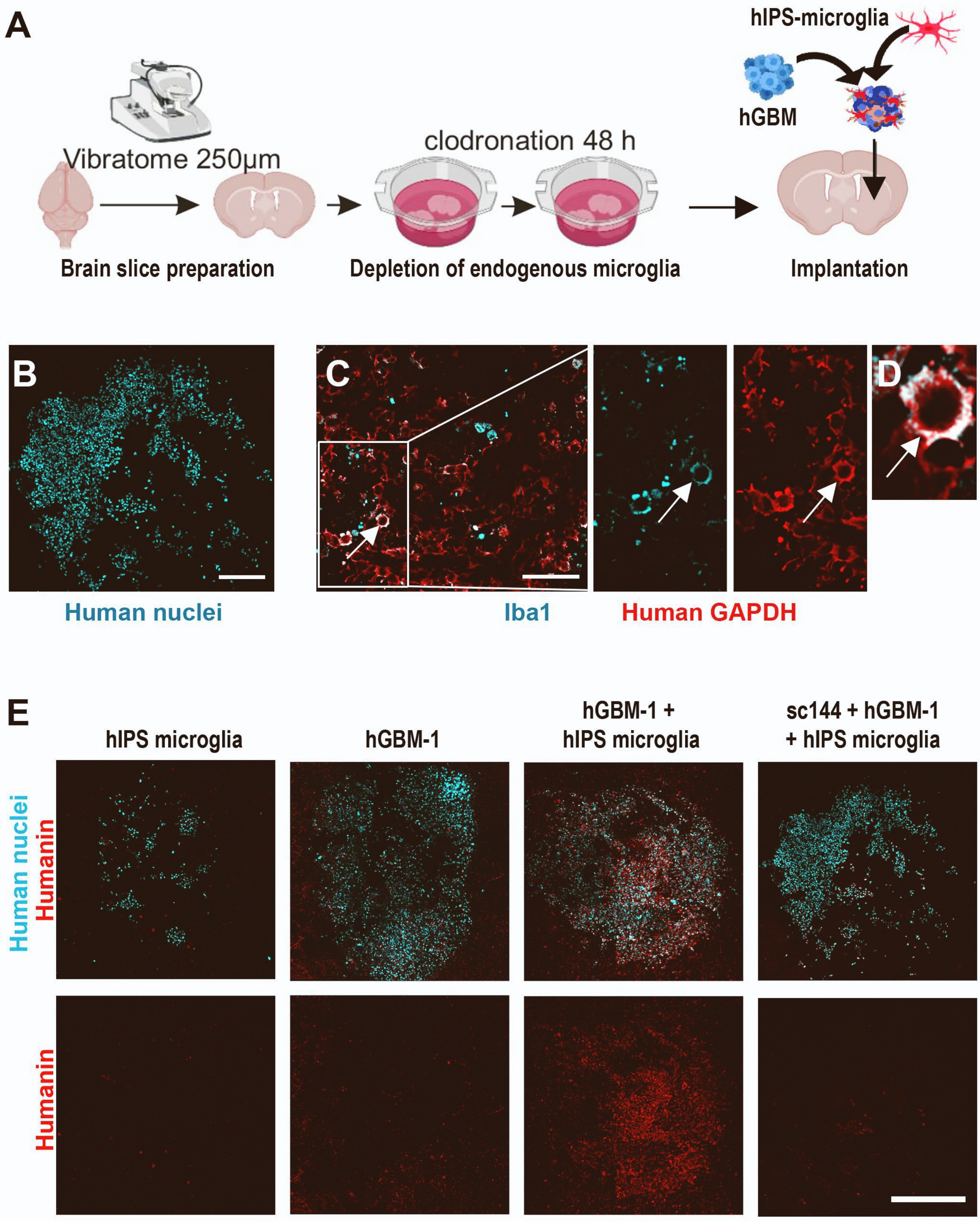
A
hIPS-microglia
hGBM
Vibratome 250μm
clodronation 48 h
Brain slice preparation
Depletion of endogenous microglia
Implantation
B
C
D
Human nuclei
Iba1
Human GAPDH
E
hIPS microglia
hGBM-1
hGBM-1 +
hIPS microglia
sc144 + hGBM-1
+ hIPS microglia
Human nuclei
Humanin
Humanin

Figure S4: The humanized brain slice model for GBM shows GP130 dependent Humanin expression upon GAM and GBM cell interaction. Related to Figures 2 and 3. (A) Schematic summary for the procedure to establish the humanized microglia ex vivo model. Briefly, brains from P14 mice were cut into 250 µm slices using a vibratome and mounted on inserts containing tissue culture medium. Clodronate filled liposomes were added into the medium for 48 h to deplete intrinsic murine microglia. Subsequently liposomes were washed away with normal medium and slices were cultured for 5 - 7 days. Then hIPS-microglia and glioma cells were mixed (1:1), co-inoculated into the organotypic brain slices and cultured for 5 days. Subsequently slices were fixed and the tumor volume was quantified. (**B)** Human cells in the brain slice culture system were readily detectable by immunofluorescence for human nuclei (cyan) or human-specific GAPDH (red). (**C-D**) The macrophage marker Iba-1 (cyan) was used to label hIPS-microglia on the organotypic brain slices, and differentiate human microglia from hGBM cells (red). The highlighted area in (**C**) was magnified, single channel recordings were provided and a single macrophage (arrow) is shown at high resolution (**D**). (**E**) For the analysis of Humanin expression in organotypic slices immunofluorescence co-staining for Humanin (red) and human nuclei (cyan) was performed and confocal tile scans of the entire tumor area were obtained. Humanin expression was upregulated when hIPS-microglia were present (see quantitative results in main figure 2a). Additional treatment of organotypic brain slices with GP130 antagonist sc144 during tumor growth reduced Humanin expression. Scale bar is 250 µm in (**B**), 100 µm in (**C**) and 500 µm in (**E**).

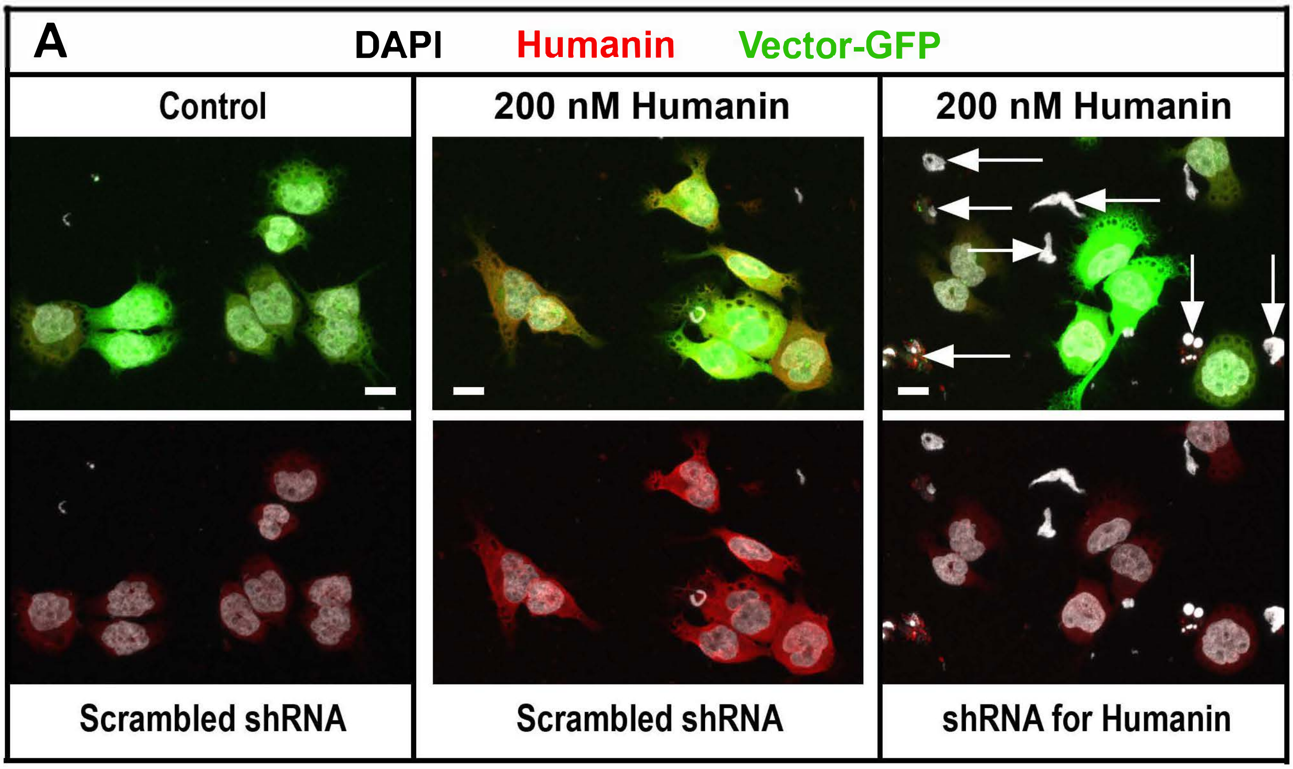
A
DAPI
Humanin
Vector-GFP
Control
200 nM Humanin
200 nM Humanin
Scrambled shRNA
Scrambled shRNA
shRNA for Humanin

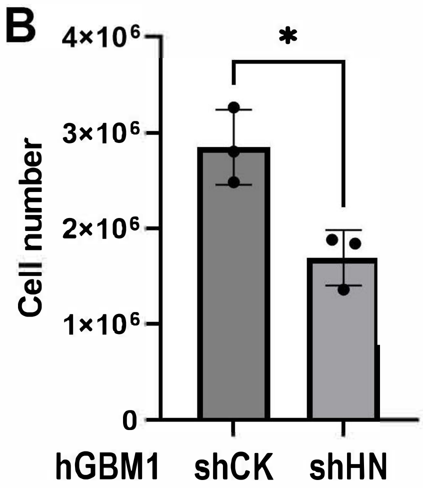
B
Cell number
4×10⁶
3×10⁶
2×10⁶
1×10⁶
0
*
hGBM1
shCK
shHN

Figure S5: Expression of a Humanin knockdown constructs deteriorates GBM cell viability. Related to Figures 2 and 3. To investigate the usability of a Humanin knockdown vector we transduced GBM cells with lentiviral particles encoding a Humanin shRNA (hGBM-1_shHN) or a scrambeled shRNA control (hGBM-1_shCK) and analyzed by immunofluorescence against Humanin or tested for cell viability by cell counting. (**A**) Upon stimulation with exogenous 200 nM HN peptide, endogenous HN expression levels (red) increased in hGBM1 cells (DAPI labelled nuclei in grey) transduced with scrambled shRNA (positively labelled in green by Vector-GFP expression). In contrast, when hGBM-1 cells were treated with specific shRNA for Humanin endogenous Humanin expression levels after HN peptide application were reduced. (**B**) Quantification of cell numbers 5 days after shRNA transduction shows a significant decrease upon knockdown of endogenous Humanin (hGBM-1_shCK) as compared to scrambled shRNA treatment (of hGBM-1_shHN). Statistical significance is shown by ttest (*$p < 0.05$); scale bar is 10 µm.

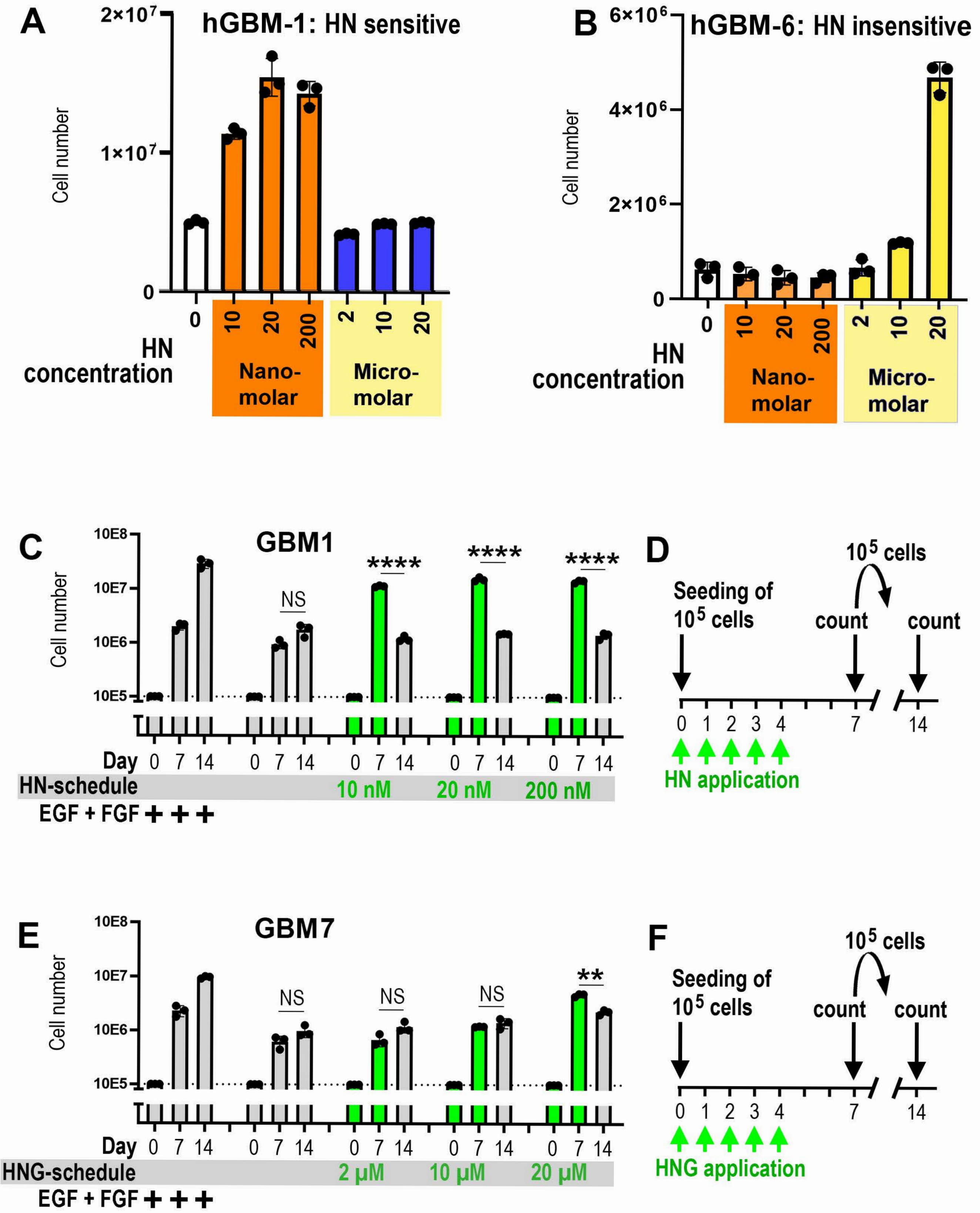

A
hGBM-1: HN sensitive
Cell number
2×10^7
1×10^7
0
HN concentration
0
10
20
200
2
10
20
Nano-molar
Micro-molar
B
hGBM-6: HN insensitive
Cell number
6×10^6
4×10^6
2×10^6
0
HN concentration
0
10
20
200
2
10
20
Nano-molar
Micro-molar
C
GBM1
Cell number
10E8
10E7
10E6
10E5
NS
****
****
****
Day 0 7 14 0 7 14 0 7 14 0 7 14 0 7 14
HN-schedule
10 nM
20 nM
200 nM
EGF + FGF + + +
D
Seeding of 10^5 cells
10^5 cells
count
count
0 1 2 3 4 7 14
HN application
E
GBM7
Cell number
10E8
10E7
10E6
10E5
NS
NS
NS
**
Day 0 7 14 0 7 14 0 7 14 0 7 14 0 7 14
HNG-schedule
2 µM
10 µM
20 µM
EGF + FGF + + +
F
Seeding of 10^5 cells
10^5 cells
count
count
0 1 2 3 4 7 14
HNG application

Figure S6: Humanin is a potent inducer of chemoresistance in Humanin insensitive GBM. Related to Figures 2 and 3. (**A, B**) hGBM cells were treated with nanomolar to micromolar concentrations of Humanin (HN) peptide daily and cells were counted after 14 days. HN-sensitive hGBM-1 cells showed increased expansion after treatment with nanomolar (but not micromolar) HN concentrations (as compared to Humanin-free controls, "0"). In contrast, HN-insensitive hGBM-6 cells showed increased expansion only after addition of 20 µM HN. (**C-F**) hGBM (10^5) cells were seeded, maintained in medium with EGF/FGF (controls) or in growth factor free medium (Ctrl.); cells under growth factor free conditions were stimulated with different concentrations of HN or Humanin-G (HNG) and counted 7 days later, then the same cells were used to repeat the procedure without addition of HN or HNG; note that 20 nM HN is sufficient to promote expansion of Humanin sensitive hGBM1, whereas 20 µM HNG were required to support the growth of the Humanin insensitive hGBM7 cells; in both cases the pharmacological effects were transient (ceased when HN/HNG were omitted after re-plating); statistical significance was assessed by One-Way-ANOVA: $^{**}p < 0.01$, $^{****}p < 0.0001$.

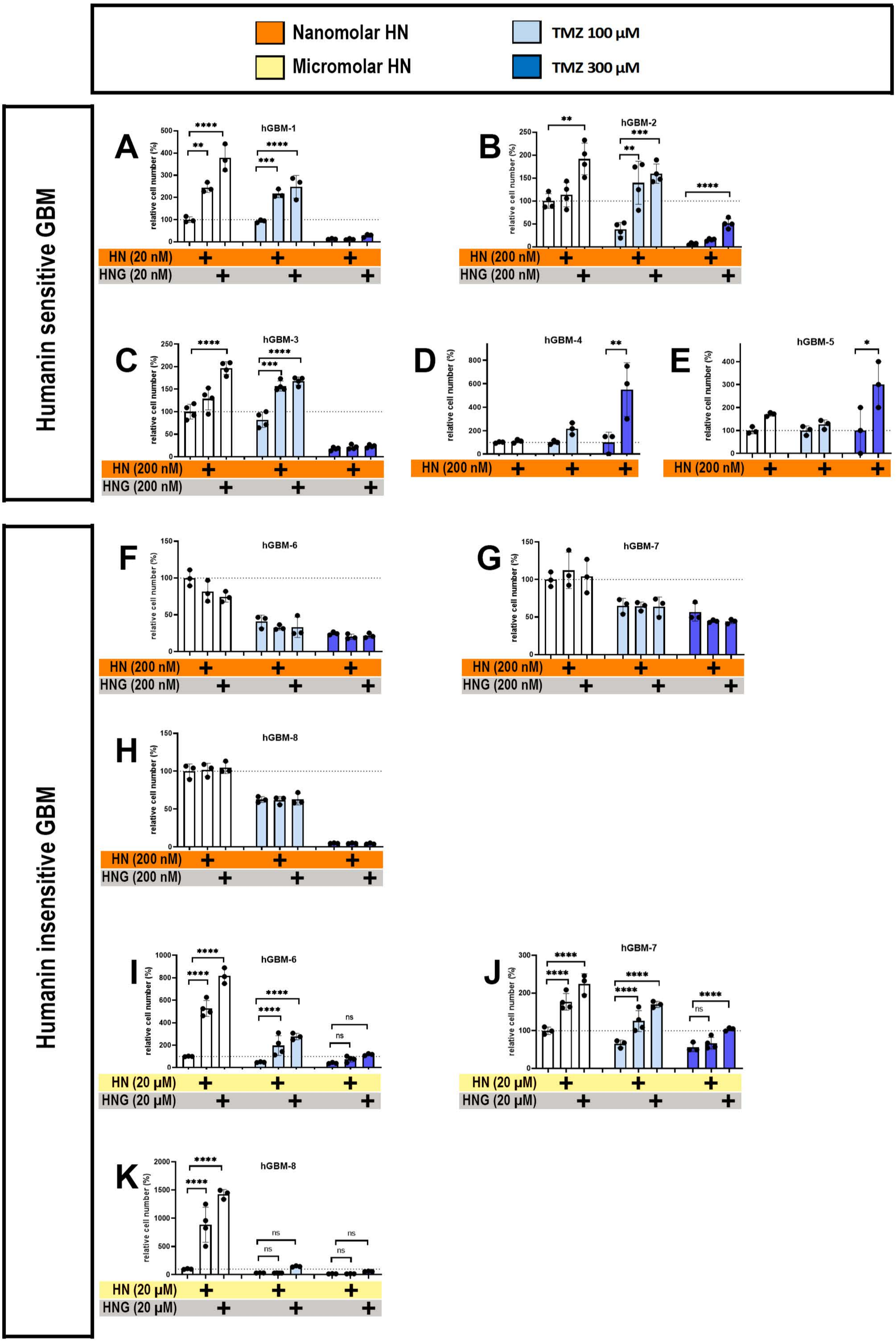

Nanomolar HN
Micromolar HN
TMZ 100 µM
TMZ 300 µM
Humanin sensitive GBM
Humanin insensitive GBM
A
hGBM-1
B
hGBM-2
C
hGBM-3
D
hGBM-4
E
hGBM-5
F
hGBM-6
G
hGBM-7
H
hGBM-8
I
hGBM-6
J
hGBM-7
K
hGBM-8
relative cell number (%)
HN (20 nM)
HNG (20 nM)
HN (200 nM)
HNG (200 nM)
HN (20 µM)
HNG (20 µM)
ns

Figure S7: Humanin promotes chemoresistance at nanomolar concentrations in Humanin-senstive GBM. Related to Figures 2 and 3. To compare cell expanssion and chemoresistance induced by Humanin, hGBM cells were exposed to different concentrations of the chemotherapeutic temozolomide (TMZ) and partially supplemented with nanomolar (**A-H**) or micromolar (**I-K**) concentration of Humanin (HN) or the pharmacologically potent Humanin mutant HNG. Cell numbers were quantified after 14 days of treatment; and were normalized to untreated control samples. (**A-E**) HN-sensitive hGBM1 - hGBM5 resisted treatment with 100 µM (A-C) or 300 µM (D, E) TMZ when nanomolar amounts of HN (or HNG) were coapplied. (**F-H**) HN-insensitive hGBM6 - hGBM8 were not resistant to TMZ when nanomolar HN (or HNG) concentrations were applied. (**I, J**), Chemoresistance of Humanin insensitive hGBM6 or hGBM7 was observed after addition of micromolar concentrations of HN (or HNG). (**K**) In hGBM-8 even high micromolar concentrations of HN or HNG did not induce TMZ resistance; statistical significance was assessed by One-Way-ANOVA: $^{**}p < 0.01$, $^{***}p < 0.001$, $^{****}p < 0.0001$.

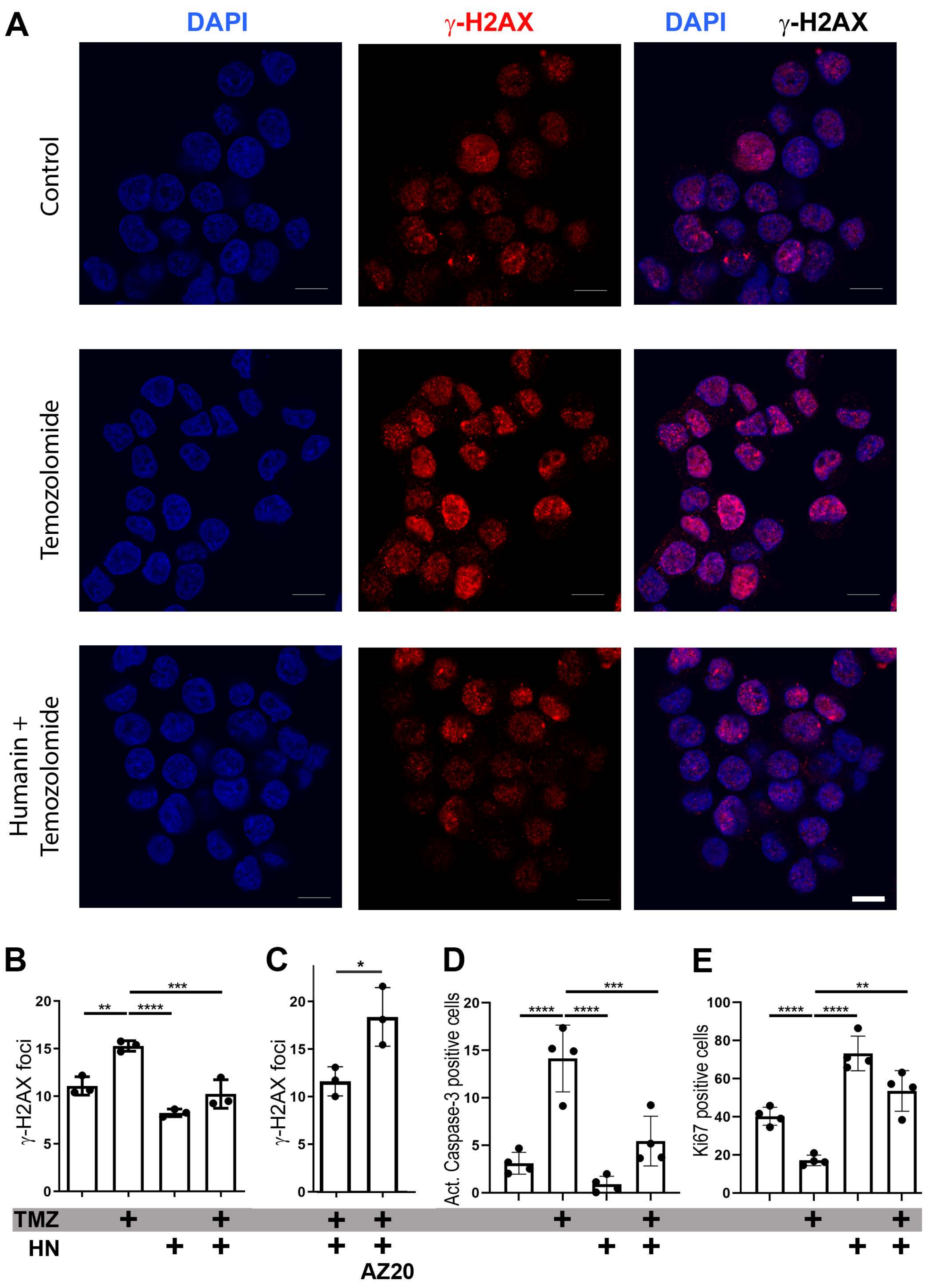

A
DAPI
γ-H2AX
DAPI γ-H2AX
Control
Temozolomide
Humanin + Temozolomide
B
C
D
E
γ-H2AX foci
Act. Caspase-3 positive cells
Ki67 positive cells
TMZ
HN
AZ20

Figure S8: Humanin promotes chemoresistance through DNA damage repair. Related to Figures 2 and 3. (**A, B**) GBM cells were left untreated (control) or were treated with Humanin (HN, 100 nM) or TMZ (100 µM) alone or in combination. Cells were stained for γH2AX as marker for DNA double-strand breaks (red) and with DAPI for nuclear labeling (grey). **(A)** TMZ treatment increased nuclear labeling for γH2AX (as expected) and this was blunted by coapplication with HN. (**B**) Quantification of the immunofluorescence labeling shown in (A). (**C**) Addition of the ATR inhibitor AZ20 to TMZ and HN treated cells attenuated DNA repair (increased nuclear γH2AX foci). (**D**) Immunostaining for activated-Caspase-3 was performed and apoptotic cells were counted. TMZ increased cell apoptosis significantly, while co-treament with HN led to near complete protection from TMZ-induced apoptosis. (**E**) Counting of Ki67-positive cell showed a significant reduction in cell cycle entry in TMZ treated GBM, compared to untreated controls. HN blunted the anti-proliferative effect of TMZ. Statistical significance was assessed by One-Way-ANOVA: $*p < 0.05$, $**p < 0.01$, $***p < 0.001$, $****p < 0.0001$; scale bar is 10 µm.

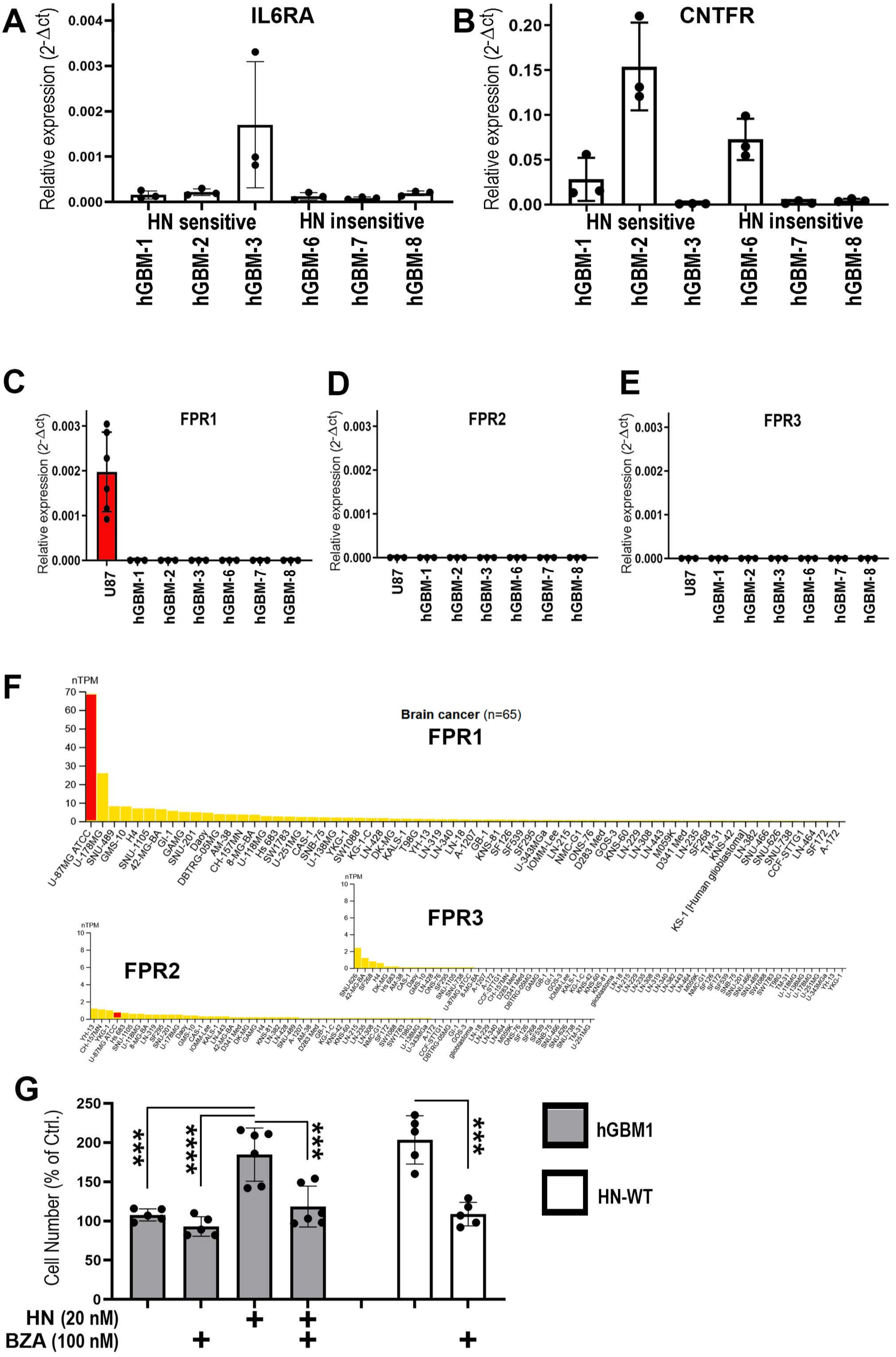
A
IL6RA
Relative expression (2^-Δct)
HN sensitive
HN insensitive
hGBM-1
hGBM-2
hGBM-3
hGBM-6
hGBM-7
hGBM-8
B
CNTFR
C
FPR1
U87
D
FPR2
E
FPR3
F
Brain cancer (n=65)
nTPM
G
Cell Number (% of Ctrl.)
hGBM1
HN-WT
HN (20 nM)
BZA (100 nM)

Figure S9: Expression and effect of GP130 in GBM. Related to Figure 3. (A-B) Quantitative RT-PCR of Humanin receptor subunits was performed in Humanin sensitive and insensitive GBM; note that a component of interleukin receptor (Il6RA) was detectable in Humanin sensitive GBM; CNTFR expression levels partly were low in both Humanin sensitive and insensitive hGBM, whereas other putative human receptors (FPR1, FPR2 or FPR3) were not detected in any hGBM (**C-E**). U87 cells (in C; red bar) served as a positive control for FPR1 detection in agreement with data from the Human Protein Atlas (HPR); HPR-derived FPR expression profiles from GBM cell lines are presented in (**F**); congruent with our qPCR data FPR1 (but not FPR2 or FPR3) is detectable in U87 cells (red bar). (**G**) The GBM cell proliferative effect of Humanin is conferred through GP130. hGBM-1, were stimulated by the addition of HN peptide (20 nM) or by HN overexpression (in HN-WT transfected hGBM-1) and part of the cells were challenged with the GP130 inhibitor bazedoxifene-A (BZA; 100 nM), which consistently abrogated the protumorigenic effect of HN as assessed by cell counting. Statistical significance is shown by One-Way-ANOVA in (***$p < 0.001$; ****$p < 0.0001$).

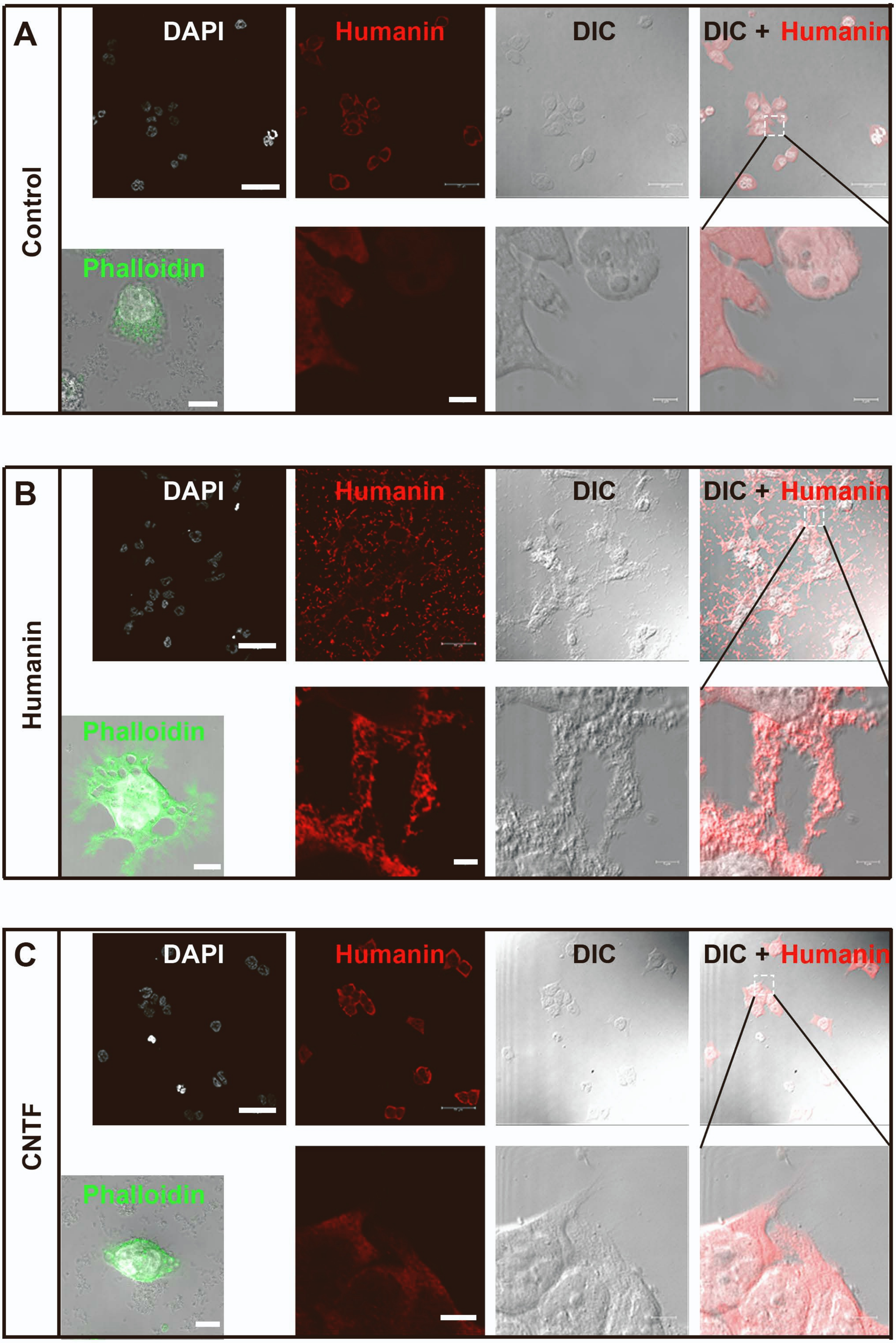

A
Control
DAPI
Humanin
DIC
DIC + Humanin
Phalloidin
B
Humanin
DAPI
Humanin
DIC
DIC + Humanin
Phalloidin
C
CNTF
DAPI
Humanin
DIC
DIC + Humanin
Phalloidin

Figure S10: Exogenous Humanin peptide induces Humanin expression in GBM. Related to Figure 3. GBM cells were treated with GP130 ligands for 20 hours (or were left untreated; control), the cytoskeleton was stained with Phalloidin (green) and nuclei with DAPI (grey) before fixation and immunostaining against Humanin (HN). (**A**-**C**) Confocal single channel recordings for DAPI (nuclei, grey), Humanin (red), F-Actin (green) and Differential Interference Contrast (DIC) are shown next overlay images; image parts are shown at higher magnification. (**A**) Untreated control cells manifest with weak Humanin signal and rounded cell appearance. (**B**) Cells treated with 200 nM HN peptide show a strong increase in intracellular Humanin expression instead, and a strong morphological change is observed (by DIC). In the overlay (DIC + Humanin) the HN-treated cells show large cell protrusions (held by Phalloidin-positive F-Actin filaments) that are full of endogenous Humanin. The GP130 ligand CNTF (**C**) ,100 ng/ml, neither changes Humanin expression levels nor cell morphology in the concentrations applied compared to untreated control (A). Scale bars are 10 µm (overview) or 1 µm (magnified).

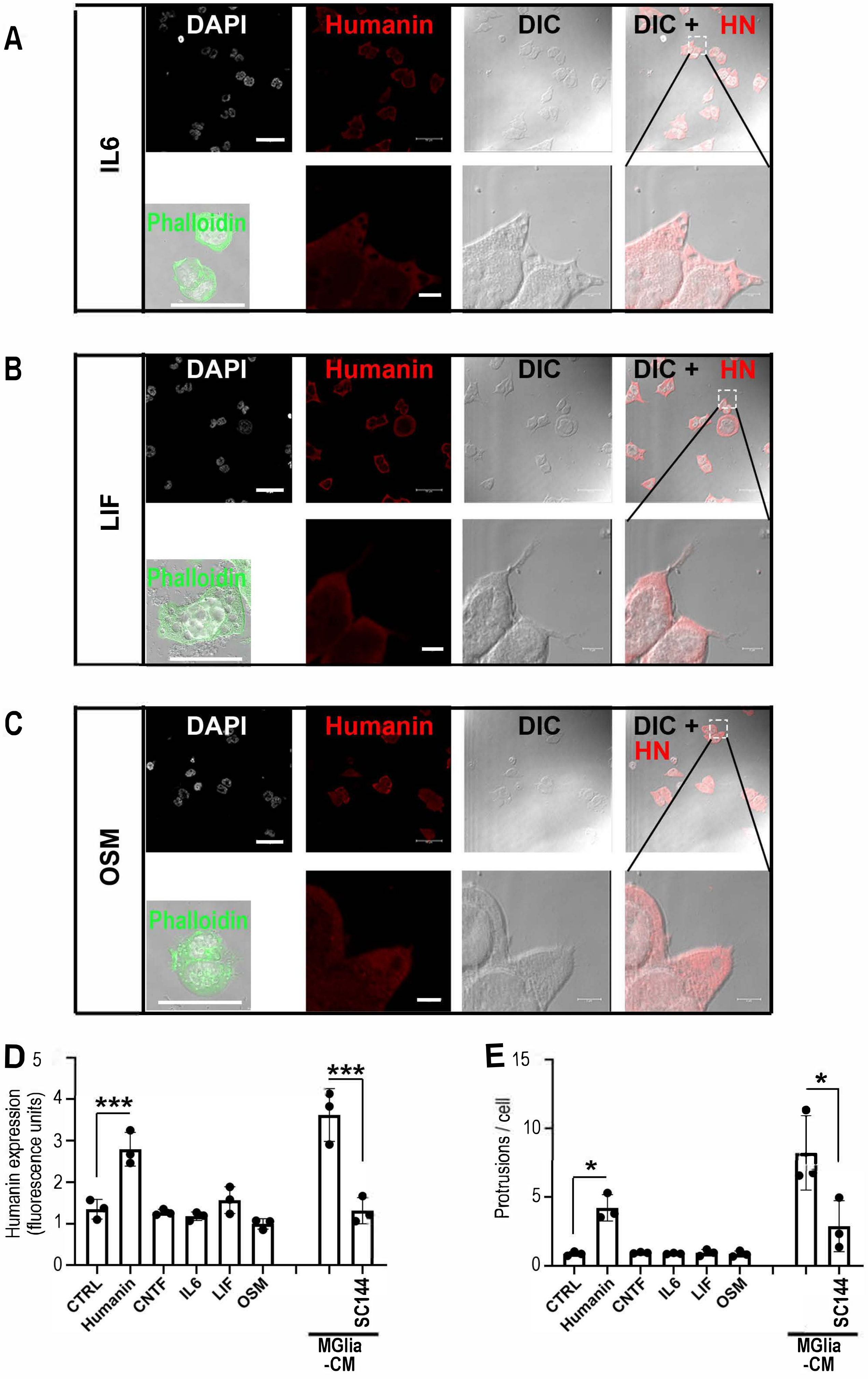

A
IL6
DAPI
Humanin
DIC
DIC + HN
Phalloidin
B
LIF
DAPI
Humanin
DIC
DIC + HN
Phalloidin
C
OSM
DAPI
Humanin
DIC
DIC + HN
Phalloidin
D
Humanin expression (fluorescence units)
***
***
CTRL
Humanin
CNTF
IL6
LIF
OSM
SC144
MGlia -CM
E
Protrusions / cell
*
*
CTRL
Humanin
CNTF
IL6
LIF
OSM
SC144
MGlia -CM

Figure S11: Exogenous Humanin peptide induces Humanin expression in GBM. Related to Figure 3. GBM cells were treated with GP130 ligands for 20 hours (or were left untreated; control), the cytoskeleton was stained with Phalloidin (green) and nuclei with DAPI (grey) before fixation and immunostaining against Humanin (HN). Activation of GP130 with 100 ng/ml IL6 (**A**), 10 ng/ml LIF (**B**), or 100 ng/ml OSM (**C**) neither changes Humanin expression levels nor cell morphology in the concentrations applied compared to untreated control (Fig. S10, A). Scale bar are 10 µm (overview) or 1 µm (magnified). (**D**) The integrated fluorescence values of Humanin staining were quantified showing a significant increase only in Humanin treated GBM (and not with other GP130 ligands) compared to negative control. Note that the incubation of hGBM cells with microglia-conditioned medium (MGlia-CM) induces a comparable significant increase in Humanin expression which was fully blocked by GP130 inhibitor sc144. (**E**) Protrusions per cell were counted and show a significantly higher number in Humanin-treated cells compared to controls or the other classical GP130 ligands. Again, this effect was copied by MGlia-CM addition and fully blockable by sc144. Scale bars are 10 µm (overview) or 1 µm (magnified). Experiments were conducted in triplicates and statistical significance was assessed by One-Way-ANOVA: *$p < 0.05$, ****$p < 0.0001$.

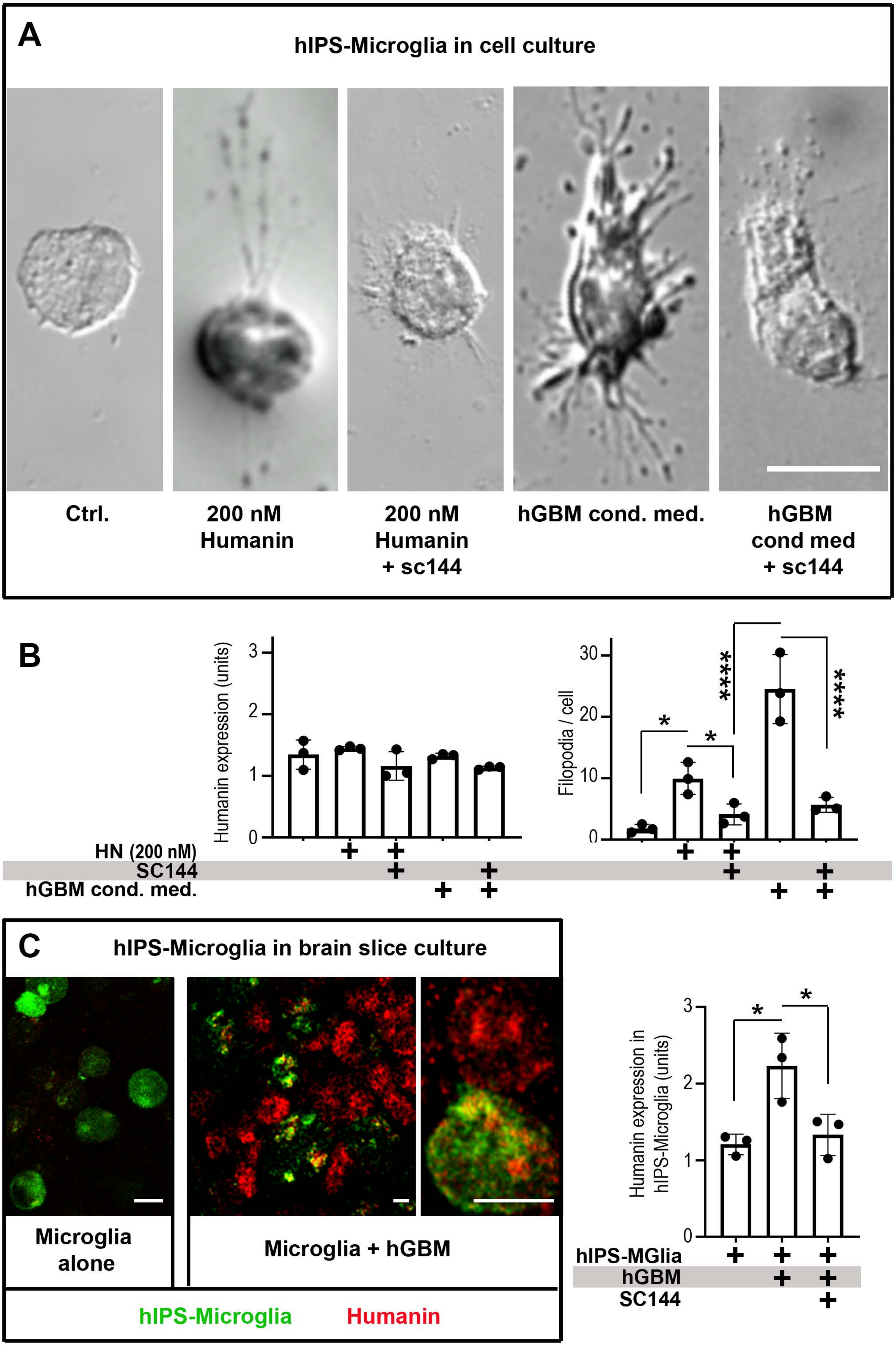

A
hIPS-Microglia in cell culture
Ctrl.
200 nM Humanin
200 nM Humanin + sc144
hGBM cond. med.
hGBM cond med + sc144
B
Humanin expression (units)
Filopodia / cell
****
*
HN (200 nM)
SC144
hGBM cond. med.
C
hIPS-Microglia in brain slice culture
Microglia alone
Microglia + hGBM
hIPS-Microglia
Humanin
Humanin expression in hIPS-Microglia (units)
hIPS-MGlia
hGBM
SC144

Figure S12: GBM cell interaction induces Humanin expression in hIPS-microglia in a GP130 dependent manner. Related to Figure 3. (A, B) Human microglia-like cells from induced pluripotent stem cells (hiPS-microglia) were left untreated or were treated with Humanin (200 nM), hGBM conditioned medium alone or in combination with GP130 inhibitor sc144. (**A**) DIC-micrograph of representative cells are shown in high magnifications. Upon Humanin treatment (but not under control conditions) hIPS-mcroglia extend protrusions (note that Humanin treatment increases cell volume and thereby changes opacity in DIC imaging). Application of hGBM- conditioned medium induces a strong morphological change in microglia showing many protrusions, which is blunted by coapplication of sc144. (**B**) Humanin expression does not change in treated versus control cells; the number of filopodia observed per cell (as compared to controls) significantly increases upon Humanin treatment or when hGBM-conditioned medium is applied; both effects are attenuated by sc144. (**C**) In brain slice cultures Humanin expression was analyzed in slices containing Microglia alone or upon interaction of Microglia (GFP+) with hGBM cells. Humanin immunostaining (in red) is increased in slices containing both hIPS-microglia and GBM cells (as compared to slices containing solely microglia). Microglial immunolabeling for Humain was quantified under the indicated experimental conditions. Scale bar are 10 µm. Experiments were conducted in triplicates and statistical significance was assessed by One-Way-ANOVA: *$p < 0.05$, ****$p < 0.0001$.

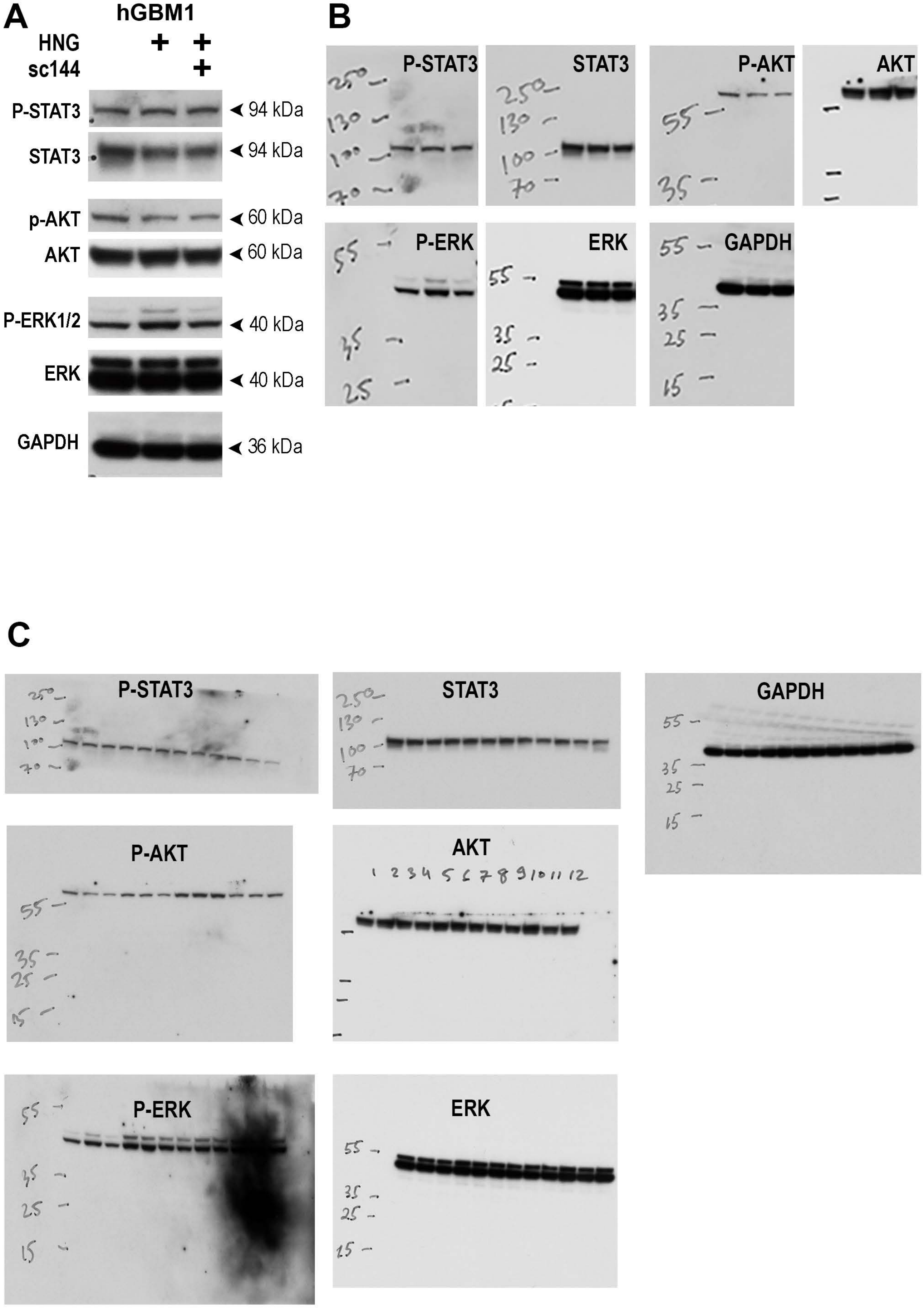
A
hGBM1
HNG
sc144
P-STAT3
STAT3
p-AKT
AKT
P-ERK1/2
ERK
GAPDH
94 kDa
94 kDa
60 kDa
60 kDa
40 kDa
40 kDa
36 kDa
B
P-STAT3
STAT3
P-AKT
AKT
P-ERK
ERK
GAPDH
C
P-STAT3
STAT3
GAPDH
P-AKT
AKT
P-ERK
ERK

Figure S13: HNG stimulates ERK-signaling through GP130 activation. Related to Figures 3 and 4. (A) hGBM1 cells were stimulated with HNG (20 nM for 16h) or were left untreated, cell pellets were harvested and used for Western blotting; note that HNG strongly induced ERK1/2 activation (phosphorylation of ERK1/2), while STAT3 was already active under control conditions and not further activated by HNG. (**B-C**) Western blotting membranes showing the selected lanes (**B**) and the complete membranes (**C**) corresponding with the cropped bands shown in (**A**) are presented.

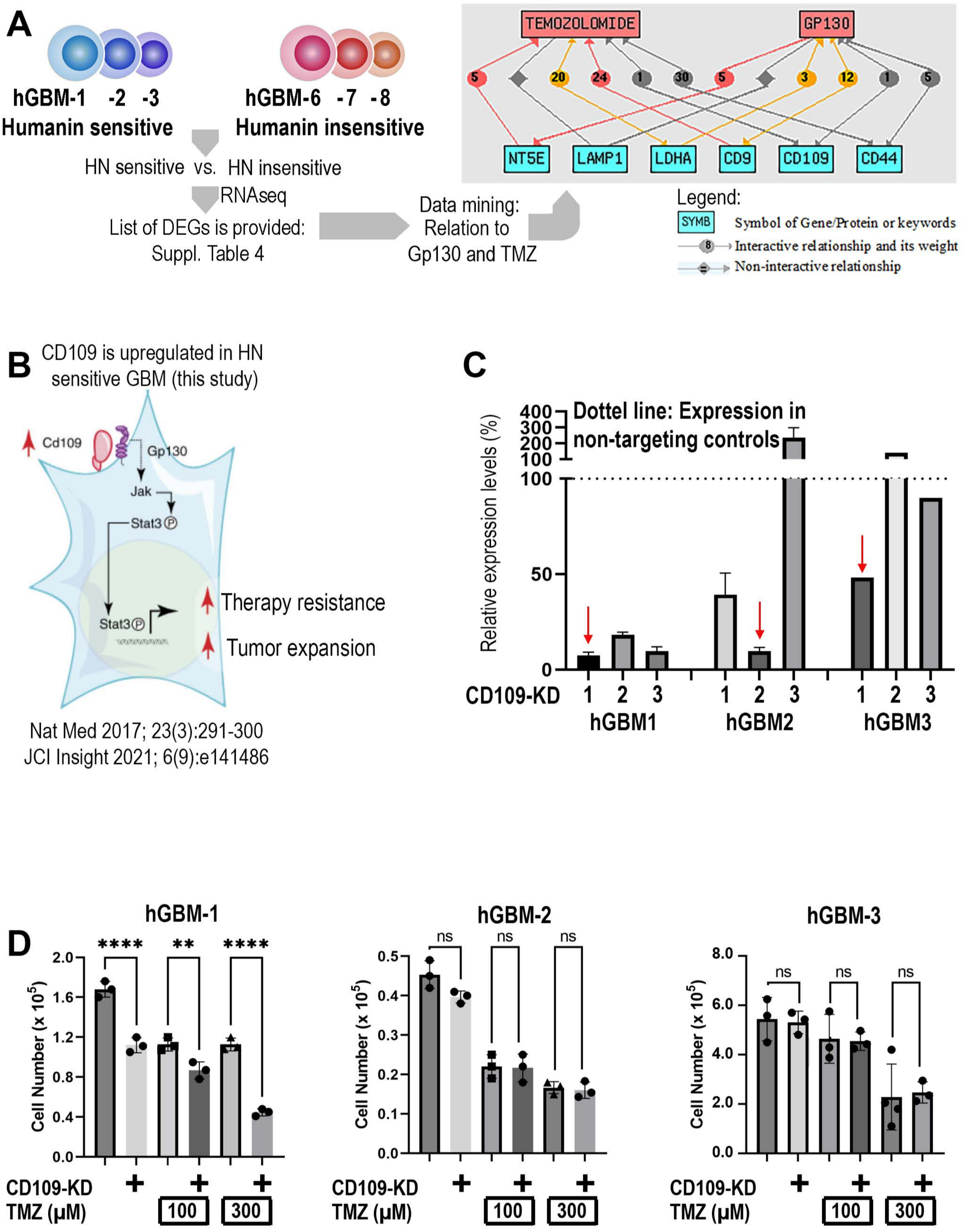
A
hGBM-1 -2 -3
Humanin sensitive
hGBM-6 -7 -8
Humanin insensitive
HN sensitive vs. HN insensitive
RNAseq
List of DEGs is provided:
Suppl. Table 4
Data mining:
Relation to
Gp130 and TMZ
TEMOZOLOMIDE
GP130
NT5E
LAMP1
LDHA
CD9
CD109
CD44
Legend:
SYMB
Symbol of Gene/Protein or keywords
Interactive relationship and its weight
Non-interactive relationship
B
CD109 is upregulated in HN sensitive GBM (this study)
Cd109
Gp130
Jak
Stat3
Therapy resistance
Tumor expansion
Nat Med 2017; 23(3):291-300
JCI Insight 2021; 6(9):e141486
C
Dottel line: Expression in non-targeting controls
Relative expression levels (%)
CD109-KD
hGBM1
hGBM2
hGBM3
D
hGBM-1
hGBM-2
hGBM-3
Cell Number (x 10^5)
CD109-KD
TMZ (μM)
100
300
ns

Figure S14: Humanin sensitive GBM are characterized by a set of genetic markers. Related to Figure 5. (**A**) Humanin sensitive (hGBM-1, -2 and -3) as well as Humanin insensitive (hGBM-6, -7 and -8) cells were grown under control conditions (72h in EGF-, bFGF-free medium), underwent transcriptomics and differentially expressed genes (DEGs) were analyzed by bioinformatics. We hypothesized that DEGs enriched in Humanin sensitive hGBM should have a role in GP130 signaling and chemoresistance (to TMZ). Hence, we explored if this gene-set (genes upregulated in Humanin sensitive hGBM) had known relations with GP130 signaling and TMZ efficacy (using bioinformatics mining by http://www.chilibot.net/; as described in *Neuro Oncol 2021. 2;23(11): 1898-1910*). This showed that Humanin sensitive GBM are characterized by high expression levels of a range of molecules (NT5E, LAMP1, LDHA, CD9, CD109, CD44) relating to both TMZ-function and GP130 signaling. (**B**) CD109 is an interaction partner for GP130, which induces STAT3-activation (phosphorylation) independently from other ligands. This has pathological relevance for melanoma and GBM (see references). (**C**) Knockdown of CD109 (CD109-KD) in Humanin sensitive GBM (hGBM-1, -2 or-3) was quantified by qPCR (versus non-targeting controls; C) and hGBMs with CD109-KD were selected (arrow) for experiments in (D). (**D**) Controls and CD109-KD cells were maintained under control conditions or exposed to TMZ (100 or 300 µM), cell numbers were counted after 5 days. This showed that CD109-KD affected TMZ-resistance in hGBM1, but not in hGBM2. Hence, Humanin sensitive hGBM express a range of molecules relating to TMZ resistance and GP130 modulation. These molecules serve as markers to identify Humanin sensitive GBM, but do not always play a pivotal functional role, whereas Humanin signaling strongly and consistently promoted chemoresistance in this subset of GBM.

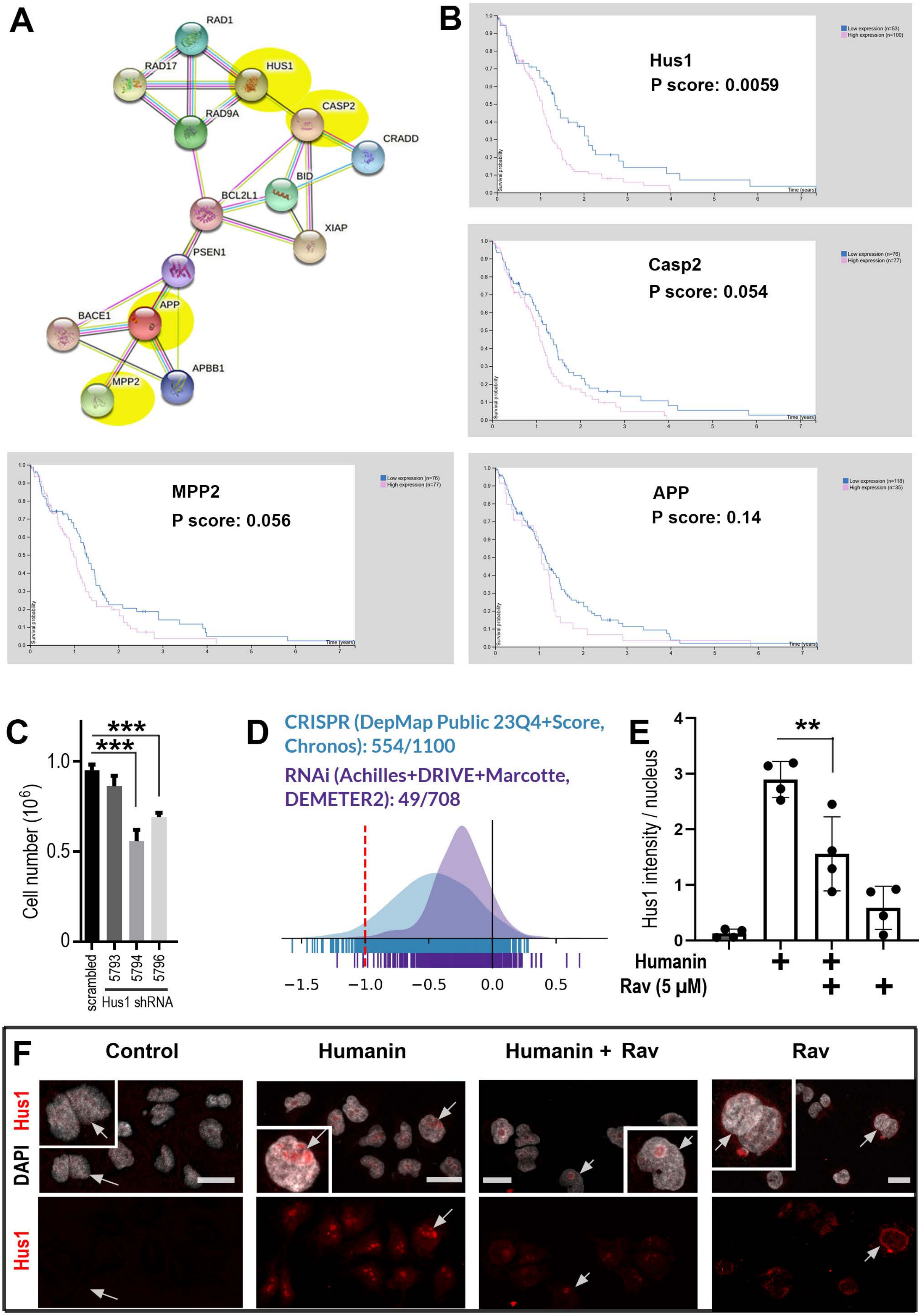

A
RAD1
RAD17
HUS1
RAD9A
CASP2
CRADD
BID
BCL2L1
XIAP
PSEN1
APP
BACE1
APBB1
MPP2
B
Hus1
P score: 0.0059
Casp2
P score: 0.054
MPP2
P score: 0.056
APP
P score: 0.14
C
Cell number (10^6)
scrambled
5793
5794
5796
Hus1 shRNA
D
CRISPR (DepMap Public 23Q4+Score, Chronos): 554/1100
RNAi (Achilles+DRIVE+Marcotte, DEMETER2): 49/708
E
Hus1 intensity / nucleus
Humanin
Rav (5 μM)
F
Control
Humanin
Humanin + Rav
Rav
Hus1
DAPI
Hus1

Figure S15: Hus1 is a pathologically relevant, Humanin induced gene. Related to Figure 5. (**A**) hGBM-1, -2 and -3 cells were stimulated with HN or vehicle (Ctrl.), underwent transcriptomics and differentially expressed genes (DEGs) were analyzed by bioinformatics; note that that HN induced e.g. nuclear signaling pathways providing 12 consistent DEGs (Suppl. Table 5), of which several components (highlighted in yellow) assembled in a network (https://string-db.org/). (**B**) From the gene list obtained in (A) the DNA clamp component HUS1 showed strong association with survival in human GBMs (https://www.proteinatlas.org/), in contrast to other genes from the same cluster (marked in yellow in A). (**C**) hGBM-1 cells were treated by three different shRNAs for Hus1 and viability was assessed by cell counting. Cell viability decreased significantly compared to scrambled control shRNA treated cells. (**D**) Interrogation of the DepMap database suggests that HUS1 knockdown/knockout compromises cell viability. (**E-F**) hGBM-1 cells were stimulated with HN (200 nM) alone or in presence with ERK-inhibitor Ravoxertinib (RAV; 5 µM), with RAV alone or were left untreated. Cells were analyzed by immunofluorescence for HUS1 (red) using confocal microscopy. Cell nuclei are labelled by DAPI (grey). The fluorescent signal intensity was quantified by ImageJ. The increase in HUS1 expression observed after HN treatment is significantly reduced by RAV. Note that HUS1 shows perinuclear localization upon RAV treatment. The insets depict close-up views of cells marked by an arrow. Scale bar is 10 µm in. Statistical significance was assessed by One-Way-ANOVA: $^{**}p < 0.01$, $^{***}p < 0.001$, $^{****}p < 0.0001$.

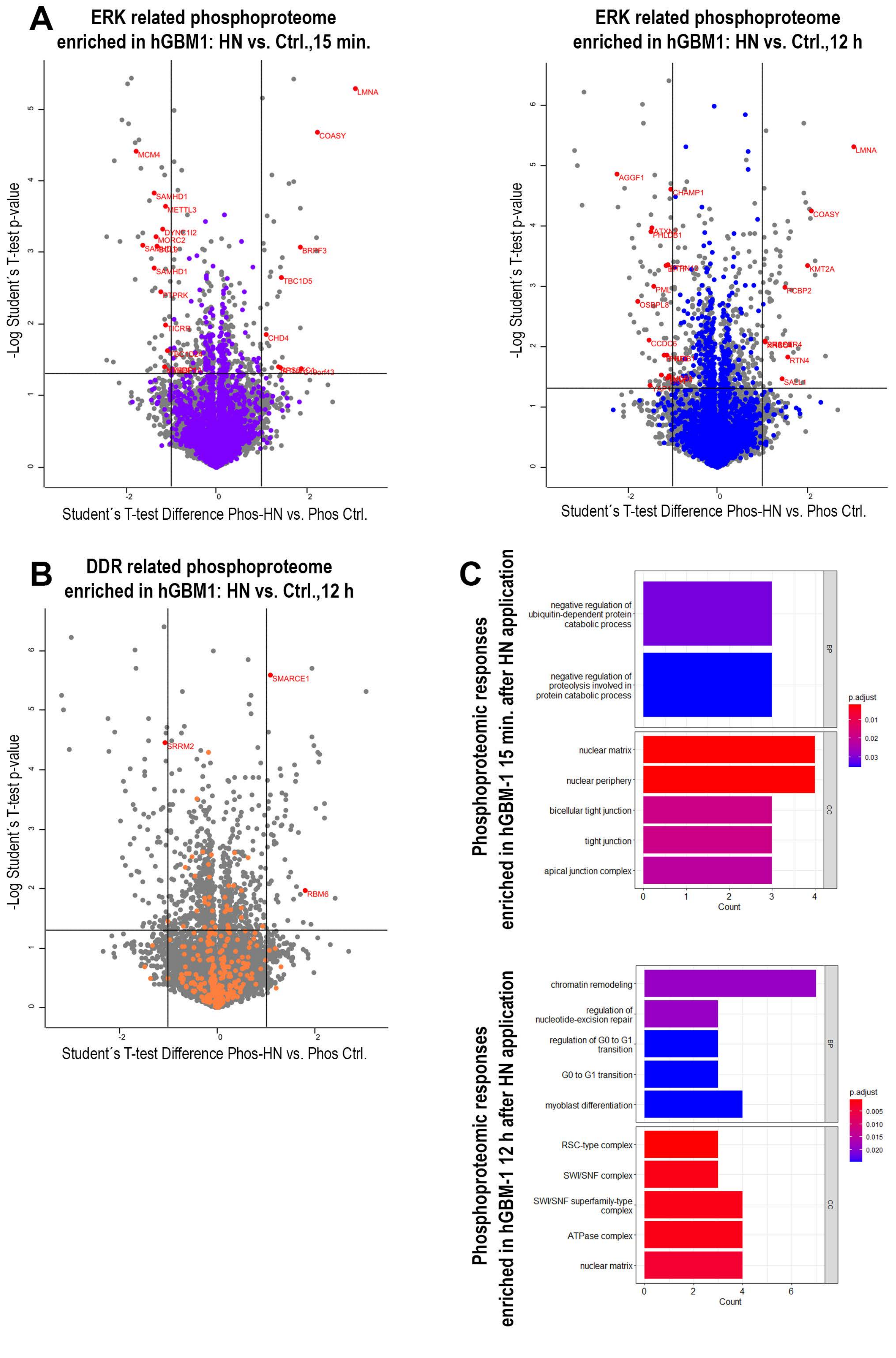
A
ERK related phosphoproteome enriched in hGBM1: HN vs. Ctrl.,15 min.
LMNA
COASY
MCM4
SAMHD1
METTL3
BRPF3
TBC1D5
PTPRK
CHD4
-Log Student´s T-test p-value
Student´s T-test Difference Phos-HN vs. Phos Ctrl.
ERK related phosphoproteome enriched in hGBM1: HN vs. Ctrl.,12 h
LMNA
AGGF1
CHAMP1
COASY
PHLDB1
KMT2A
PML
PCBP2
OSBPL8
RTN4
-Log Student´s T-test p-value
Student´s T-test Difference Phos-HN vs. Phos Ctrl.
B
DDR related phosphoproteome enriched in hGBM1: HN vs. Ctrl.,12 h
SMARCE1
SRRM2
RBM6
-Log Student´s T-test p-value
Student´s T-test Difference Phos-HN vs. Phos Ctrl.
C
Phosphoproteomic responses enriched in hGBM-1 15 min. after HN application
negative regulation of ubiquitin-dependent protein catabolic process
negative regulation of proteolysis involved in protein catabolic process
nuclear matrix
nuclear periphery
bicellular tight junction
tight junction
apical junction complex
BP
CC
p.adjust
Count
Phosphoproteomic responses enriched in hGBM-1 12 h after HN application
chromatin remodeling
regulation of nucleotide-excision repair
regulation of G0 to G1 transition
G0 to G1 transition
myoblast differentiation
RSC-type complex
SWI/SNF complex
SWI/SNF superfamily-type complex
ATPase complex
nuclear matrix
BP
CC
p.adjust
Count

Figure S16: Phosphoproteome analysis confirms activation of ERK and DDR pathways in GBM cells downstream of Humanin-GP130 signaling. Related to Figure 5. To determine the signaling cues downstream of Humanin-induced GP130 activation, we investigated global protein phosphorylation by mass spectrometry. (**A, B**) Volcano plot with phosphopeptides quantified by label-free phosphoproteome analysis. Phosphopeptides are plotted according to p-value and difference caused by treatment of hGBM-1 cells with HN (200 nM) for 15 min or 12 hours in comparison to untreated cells (n=3 independent experiments). Phosphopeptides that change significantly in abundance (ttest p-value < 0,05; |ttest Difference|> 1) and contain phosphosites (blue) matching ERK (A-B) or ATM/ATR consensus motifs for DNA damage response (DDR) are shown as red dots; significantly changed phosphopeptides without consensus motifs are shown in black. (**C**) Phosphoproteomic responses enriched in hGBM-1 cells were obtained by Gene Ontology (GO) enrichment analysis showing involved biological processes (BP) and cellular components (CC). HN treatment protects hGBM-1 cells from proteolysis and ubiquitin-dependent degradation at short incubation time (15 min) while it is involved in chromatin remodeling and cell cycle control.

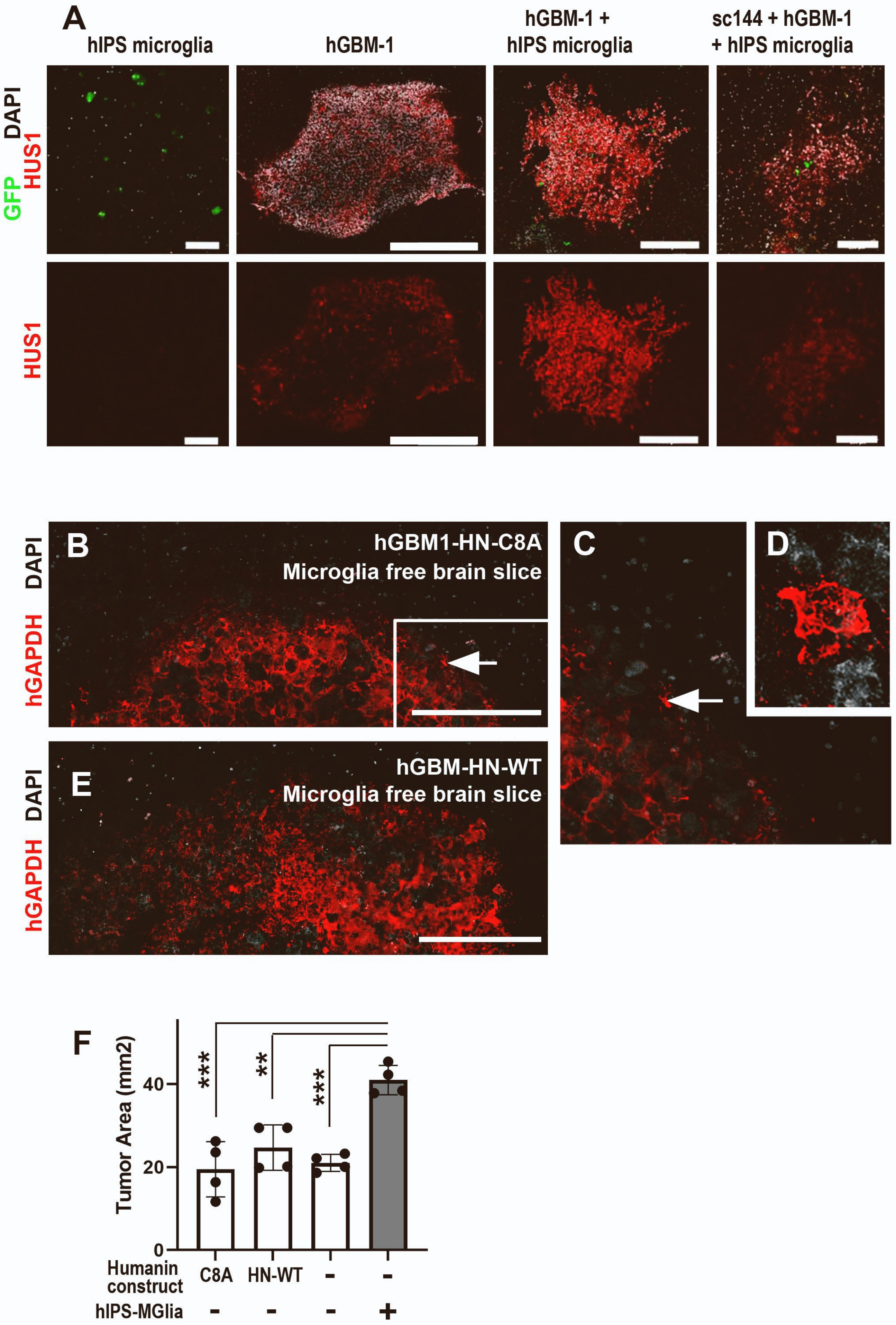
A
hIPS microglia
hGBM-1
hGBM-1 +
hIPS microglia
sc144 + hGBM-1
+ hIPS microglia
GFP HUS1 DAPI
HUS1
B
hGBM1-HN-C8A
Microglia free brain slice
DAPI
hGAPDH
C
D
E
hGBM-HN-WT
Microglia free brain slice
DAPI
hGAPDH
F
Tumor Area (mm2)
40
20
0
***
**
***
Humanin construct
C8A
HN-WT
-
-
hIPS-MGlia
-
-
-
+

Figure S17: Interaction of GBM with GAM induces Hus1 expression in a GP130 dependent manner. Related to Figures 5 and 6. (A) Confocal scanning in organ cultures under different conditions (see top row in figure) shows immunofluorescence for HUS1 (red); note that hIPS-microglia express GFP. HUS1 is undetectable in slices containing hIPS-microglia alone, detectable at lower level in slices containing hGBM alone and most prominent when hIPS-microglia are co-inoculated with hGBM; in slices containing both hGBM and hIPS-microglia HUS1 expression is blunted by sc144. (**B-F**) hGBM1-HN-C8A or hGBM-HN-WT cells were implanted in microglia depleted brain slices and analyzed by immunofluorescence against human-specific GAPDH (hGAPDH in red). (**B**) In hGBM1-HN-C8A brain slice cultures single invasive cells were scarce (see inset marked by arrow and magnifications of it in **C** and **D**) and this did not change with hGBM-HN-WT tumors (**E**). (**F**) Invasive hGAPDH-positive GBM cells and the main tumor mass was quantified in brain slices with or without coculture of hIPS-microglia (GFP+ cells were excluded when measuring invasiveness). No difference was observed when measuring the overall invasive tumor area upon implantation of hGBM1-HN-C8A, hGBM-HN-WT or parental hGBM-1 cells in microglia-depleted slices. However, when co-implanted with hIPS-microglia the tumor area of hGBM-1 cells was significantly increased (n = 4 organotypic brain slices per experimental group). Scale bars in (**A**) hIPS-microglia, hGBM-1 and hGBM1+ hIPS-microglia samples represent 500 µm, scale bars in hGBM-1+hIPS-MG+SC144 are 250 µm; scales are 500 µm in (**B** and **E**), 10 µm in (**D**). Statistical significance in (**F**) was assessed by One-Way-ANOVA: $^{**}p < 0.01$, $^{***}p < 0.001$.

A

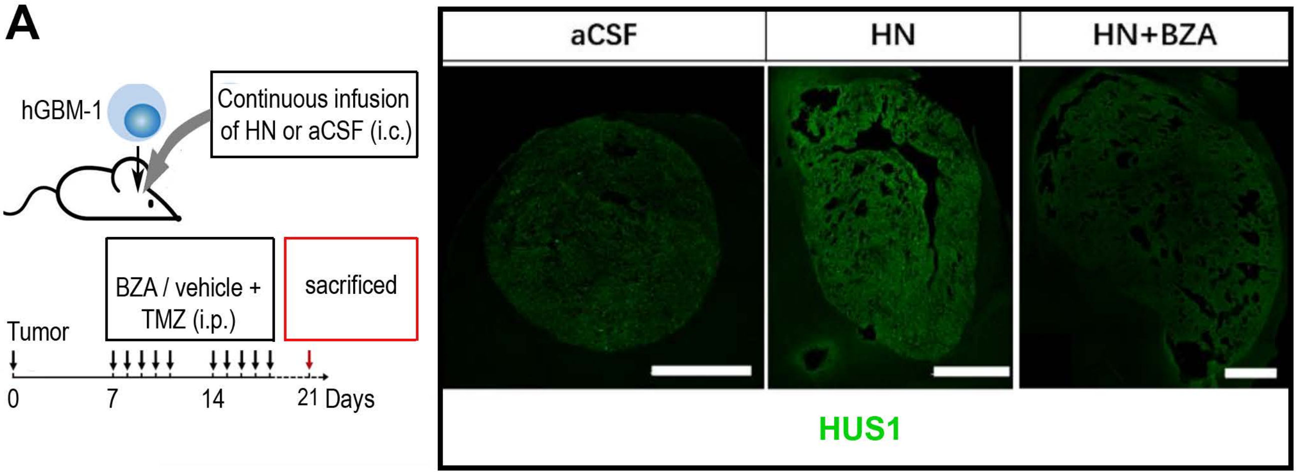
hGBM-1
Continuous infusion of HN or aCSF (i.c.)
BZA / vehicle + TMZ (i.p.)
sacrificed
Tumor
0
7
14
21 Days
aCSF
HN
HN+BZA
HUS1

Figure S18: Humanin-GP130 signaling induces HUS1 expression in GBM. Related to Figure 6. Orthotopic GBM were continuously (throughout the entire experimental time course) infused with Humanin or artificial cerebrospinal fluid (aCSF, as a control); 7 days after GBM inoculation (when tumor take was verified) mice were i.p. injected with TMZ and co-treated either with BZA or vehicle (as summarized in the schematic); mouse brains were immunofluorescently labeled for HUS1 after 21 days; note that intratumoral infusion of Humanin but not aCSF infusion promoted HUS1 expression and that the Humanin induced effect was blunted by cotreatment with BZA. Scale bars are 1 mm.

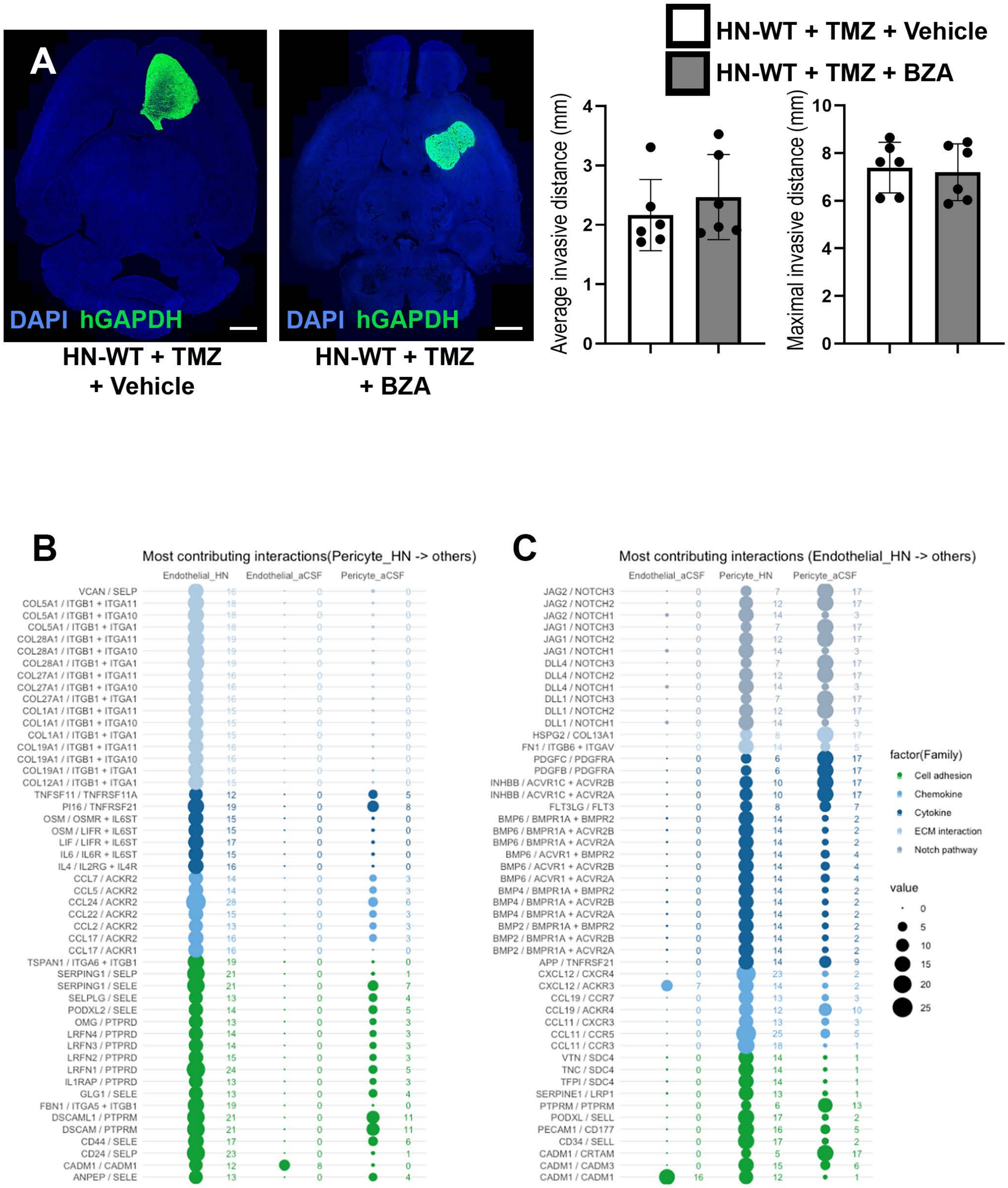
A
DAPI hGAPDH
HN-WT + TMZ + Vehicle
HN-WT + TMZ + BZA
Average invasive distance (mm)
Maximal invasive distance (mm)
B
Most contributing interactions(Pericyte_HN -> others)
Endothelial_HN
Endothelial_aCSF
Pericyte_aCSF
VCAN / SELP
COL5A1 / ITGB1 + ITGA11
COL5A1 / ITGB1 + ITGA10
COL5A1 / ITGB1 + ITGA1
COL28A1 / ITGB1 + ITGA11
COL28A1 / ITGB1 + ITGA10
COL28A1 / ITGB1 + ITGA1
COL27A1 / ITGB1 + ITGA11
COL27A1 / ITGB1 + ITGA10
COL27A1 / ITGB1 + ITGA1
COL1A1 / ITGB1 + ITGA11
COL1A1 / ITGB1 + ITGA10
COL1A1 / ITGB1 + ITGA1
COL19A1 / ITGB1 + ITGA11
COL19A1 / ITGB1 + ITGA10
COL19A1 / ITGB1 + ITGA1
COL12A1 / ITGB1 + ITGA1
TNFSF11 / TNFRSF11A
PI16 / TNFRSF21
OSM / OSMR + IL6ST
OSM / LIFR + IL6ST
LIF / LIFR + IL6ST
IL6 / IL6R + IL6ST
IL4 / IL2RG + IL4R
CCL7 / ACKR2
CCL5 / ACKR2
CCL24 / ACKR2
CCL22 / ACKR2
CCL2 / ACKR2
CCL17 / ACKR2
CCL17 / ACKR1
TSPAN1 / ITGA6 + ITGB1
SERPING1 / SELP
SERPING1 / SELE
SELPLG / SELE
PODXL2 / SELE
OMG / PTPRD
LRFN4 / PTPRD
LRFN3 / PTPRD
LRFN2 / PTPRD
LRFN1 / PTPRD
IL1RAP / PTPRD
GLG1 / SELE
FBN1 / ITGA5 + ITGB1
DSCAML1 / PTPRM
DSCAM / PTPRM
CD44 / SELE
CD24 / SELP
CADM1 / CADM1
ANPEP / SELE
C
Most contributing interactions (Endothelial_HN -> others)
Endothelial_aCSF
Pericyte_HN
Pericyte_aCSF
JAG2 / NOTCH3
JAG2 / NOTCH2
JAG2 / NOTCH1
JAG1 / NOTCH3
JAG1 / NOTCH2
JAG1 / NOTCH1
DLL4 / NOTCH3
DLL4 / NOTCH2
DLL4 / NOTCH1
DLL1 / NOTCH3
DLL1 / NOTCH2
DLL1 / NOTCH1
HSPG2 / COL13A1
FN1 / ITGB6 + ITGAV
PDGFC / PDGFRA
PDGFB / PDGFRA
INHBB / ACVR1C + ACVR2B
INHBB / ACVR1C + ACVR2A
FLT3LG / FLT3
BMP6 / BMPR1A + BMPR2
BMP6 / BMPR1A + ACVR2B
BMP6 / BMPR1A + ACVR2A
BMP6 / ACVR1 + BMPR2
BMP6 / ACVR1 + ACVR2B
BMP6 / ACVR1 + ACVR2A
BMP4 / BMPR1A + BMPR2
BMP4 / BMPR1A + ACVR2B
BMP4 / BMPR1A + ACVR2A
BMP2 / BMPR1A + BMPR2
BMP2 / BMPR1A + ACVR2B
BMP2 / BMPR1A + ACVR2A
APP / TNFRSF21
CXCL12 / CXCR4
CXCL12 / ACKR3
CCL19 / CCR7
CCL19 / ACKR4
CCL11 / CXCR3
CCL11 / CCR5
CCL11 / CCR3
VTN / SDC4
TNC / SDC4
TFPI / SDC4
SERPINE1 / LRP1
PTPRM / PTPRM
PODXL / SELL
PECAM1 / CD177
CD34 / SELL
CADM1 / CRTAM
CADM1 / CADM3
CADM1 / CADM1
factor(Family)
Cell adhesion
Chemokine
Cytokine
ECM interaction
Notch pathway
value

Figure S19: HN-induced GP130 signaling does not affect in vivo GBM cell invasion but alters crosstalk of pericyte with endothelial cells. Related to Figure 7. Mice with established, orthotopic HN-WT tumors received TMZ (50 mg/kg) and were either co-treated with BZA or vehicle; after three weeks of tumor growth samples were collected. (**A**) Brain sections were immunostained with an antibody specifically detecting human hGAPDH (highlighting xenografted human GBM). Immunofluorescence pictures of horizontal brain sections are shown with DAPI-positive nuclei (blue). Brain sections were inspected for single invasive tumor cells and the average and the maximal invasive distance was quantified. No significant difference was obtained by students ttest between the experimental groups (n = 6 animals per group). (**B, C**) Mouse GBM were intracerebrally treated with Humanin peptide or vehicle (aCSF). To investigate Humanin-dependent cell-cell communication in the brain tumor vasculature, the cell type specific transcriptome was obtained after FACS sorting of tumor-derived pericytes or endothelial cells. Cell-cell communication of purified cells was analyzed from transcriptomic data using ICELLNET and the murine CellPhoneDB as a reference (data are from n=4 mice for Pericyte_HN and n=3 mice for Percyte_aCSF; n=2 mice for Endothelial_HN or Endothelial_aCSF). Interestingly, upon Humanin treatment pericyte communicate by upregulating several ligands for the GP130 receptor on endothelial cells. Also, many extracellular matrix and cell adhesion interactions between the two cell types get induced. (**B**) HN-treated pericytes (Pericyte_HN) show most interactions with HN-treated endothelial cells (Endothelial_HN), while change of interactions with themselves (Percyte_aCSF) or with endothelial cells (Endothelial_aCSF) without HN-treatment are minimal. (**C**) HN-treated endothelial cells communicate with HN-treated pericytes through BMP, NOTCH and PDGFR -pathways. Plotted bubble bubble sizes indicate communication scores, while bubble color represents the classification (family) of different ligand-receptor pairs. Dotplots displaying the top 50 statistically significant interactions are shown. Scale bars in (A) represent 1.3 mm.

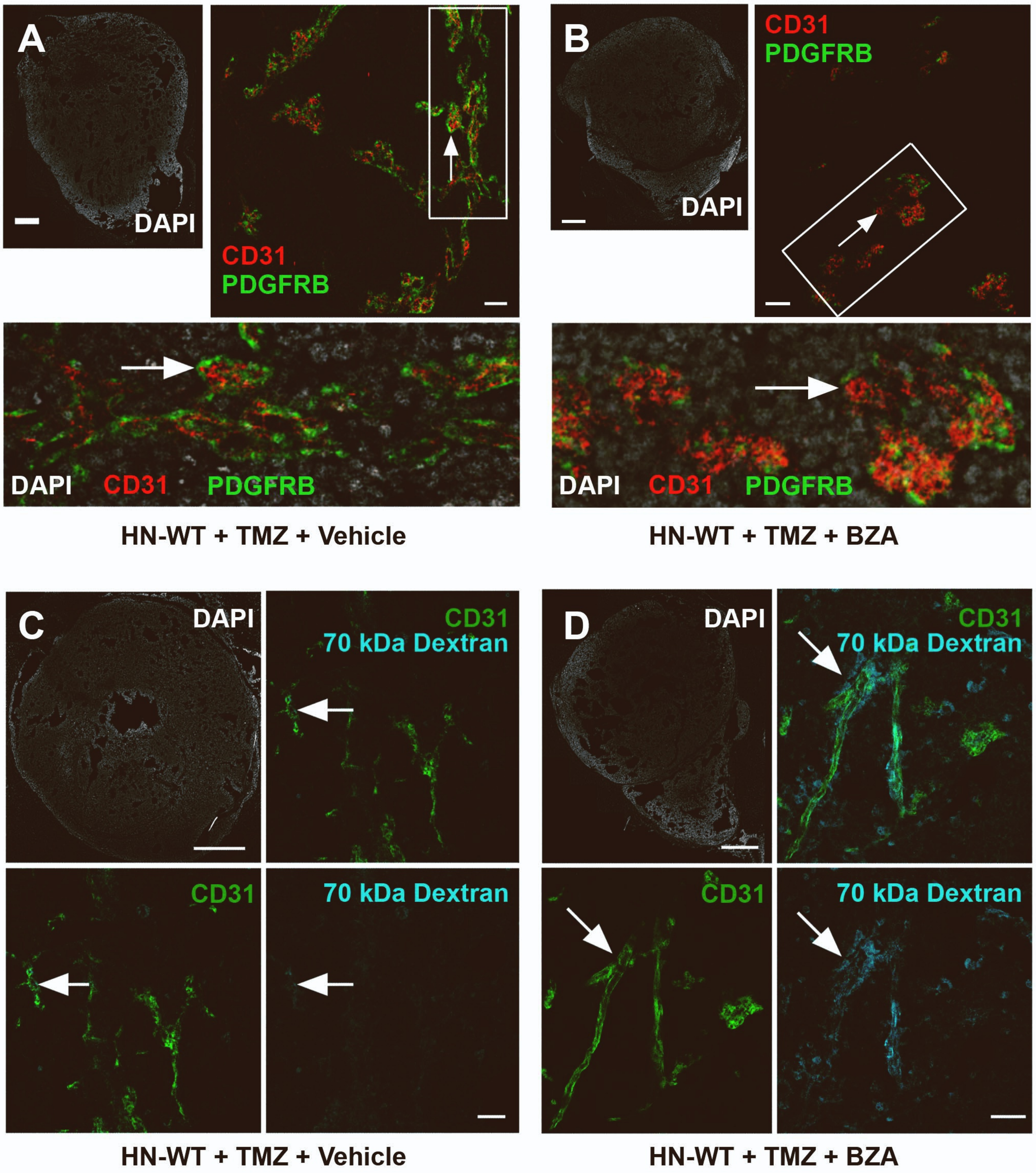
A
DAPI
CD31
PDGFRB
DAPI CD31 PDGFRB
HN-WT + TMZ + Vehicle
B
DAPI
CD31
PDGFRB
DAPI CD31 PDGFRB
HN-WT + TMZ + BZA
C
DAPI
CD31
70 kDa Dextran
CD31
70 kDa Dextran
HN-WT + TMZ + Vehicle
D
DAPI
CD31
70 kDa Dextran
CD31
70 kDa Dextran
HN-WT + TMZ + BZA

Figure S20: HN-induced GP130 signaling in GBM cell affects pericyte coverage and blood-tumor barrier formation. Related to Figure 7. (**A, B**) The tumor vasculature of orthotopically implanted human GBM cells was investigated by immunofluorescence staining against vascular endothelial (CD31) and mural (PDGRB) cells. Blockage of GP130 by BZA in mice (implanted with HN-WT cells and TMZ treated) reduced the coverage (arrows) of endothelia cells (CD31) by pericytes (PDGFRB), this was not observed in vehicle treated controls. **(D-E)** Intravenous administration of 70kDa Dextran (blue) in advanced GBM was used to assess the blood tumor barrier. In BZA-treated tumors fluorescently-labelled 70kDa dextran (blue) can be observed in the tumor parenchyma next to the CD31+vessel area (arrows), this was much less apparent in vehicle-treated mice. Scale bars in the overviews (in A, B) are 500 µm; and 20 µm in all other micrographs.
